# Rehabilitation delivery models to foster healthy ageing—a scoping review

**DOI:** 10.3389/fresc.2024.1307536

**Published:** 2024-04-05

**Authors:** Vanessa Seijas, Roxanne Maritz, Patricia Fernandes, Renaldo M. Bernard, Luz Helena Lugo, Jerome Bickenbach, Carla Sabariego

**Affiliations:** ^1^ Faculty of Health Sciences and Medicine, University of Lucerne, Lucerne, Switzerland; ^2^ Center for Rehabilitation in Global Health Systems (WHO Collaborating Center), Faculty of Health Sciences and Medicine, University of Lucerne, Lucerne, Switzerland; ^3^ Swiss Paraplegic Research, Nottwil, Switzerland; ^4^ Department of Clinical Medicine, Federal University of Parana, Parana, Brazil; ^5^ Rehabilitation in Health Research Group, University of Antioquia, Medellin, Colombia

**Keywords:** healthy ageing, rehabilitation, delivery of health care, delivery models, older adults, aged

## Abstract

**Introduction:**

Rehabilitation is essential to foster healthy ageing. Older adults have unique rehabilitation needs due to a higher prevalence of non-communicable diseases, higher susceptibility to infectious diseases, injuries, and mental health conditions. However, there is limited understanding of how rehabilitation is delivered to older adults. To address this gap, we conducted a scoping review to describe rehabilitation delivery models used to optimise older adults' functioning/functional ability and foster healthy ageing.

**Methods:**

We searched Medline and Embase (January 2015 to May 2022) for primary studies published in English describing approaches to provide rehabilitation to older adults. Three authors screened records for eligibility and extracted data independently and in duplicate. Data synthesis included descriptive quantitative analysis of study and rehabilitation provision characteristics, and qualitative analysis to identify rehabilitation delivery models.

**Results:**

Out of 6,933 identified records, 585 articles were assessed for eligibility, and 283 studies with 69,257 participants were included. We identified six rehabilitation delivery models: outpatient (24%), telerehabilitation (22%), home (18.5%), community (16.3%), inpatient (14.6%), and eldercare (4.7%). These models often involved multidisciplinary teams (31.5%) and follow integrated care principles (30.4%). Most studies used a disease-centred approach (59.0%), while studies addressing multimorbidity (6.0%) and prevalent health problems of older adults, such as pain, low hearing, and vision, or incontinence were scarce. The most frequently provided interventions were therapeutic exercises (54.1%), self-management education (40.1%), and assessment of person-centred goals (40%). Other interventions, such as assistive technology (8.1%) and environmental adaptations (7.4%) were infrequent.

**Conclusions:**

Focusing on primary studies, this scoping review provides an overview of rehabilitation delivery models that are used to foster healthy ageing and highlights research gaps that require further attention, including a lack of systematic assessment of functioning/functional ability, a predominance of disease-centred rehabilitation, and a scarcity of programmes addressing prevalent issues like pain, hearing/vision loss, fall prevention, incontinence, and sexual dysfunctions. Our research can facilitate evidence-based decision-making and inspire further research and innovation in rehabilitation and healthy ageing. Limitations of our study include reliance on published research to infer practice and not assessing model effectiveness. Future research in the field is needed to expand and validate our findings.

## 1 Introduction

Worldwide rehabilitation needs are increasing due to, among others, the global demographic shift towards rapid population ageing (1). The landmark World Report on Ageing and Health (WRAH) showed that the proportion and the absolute number of older adults are increasing exponentially. By 2050, people older than 60 will exceed 30% of the population in many high- and middle-income countries (2), and globally, this proportion will rise from 14% in 2022 to 21.4% in 2050 (3). Moreover, the population ageing rate is now greater. For example, France had 150 years to adapt from 10% to 20% in the proportion of the population older than 60; however, countries like Brazil and India will only have 20 years to adapt (2).

In 2019, 1 in 3 persons (2.41 billion) had health conditions that would benefit from rehabilitation during their disease. Musculoskeletal and neurological conditions, sensory impairments, and chronic respiratory diseases are the most significant contributors to the needs of people older than 65 (1). However, rehabilitation needs remain largely unmet across all age groups. In low- and middle-income countries (LMICs), up to 50% of people do not receive the rehabilitation they need (4). Recognising the urgency of strengthening the integration of rehabilitation into health systems, and, inter alia, that the need for rehabilitation is increasing due to the global demographic shift towards a rapidly ageing population, the World Health Assembly (WHA) adopted a landmark resolution on “Strengthening Rehabilitation in Health Systems” in May 2023. The resolution states that the expansion of rehabilitation to all levels of health care, is essential to ensure the availability and affordability of quality and timely rehabilitation services for all (5).

Ageing populations have unique rehabilitation needs related to a higher prevalence of noncommunicable diseases (NCDs), the increased susceptibility to severe consequences from infectious diseases like coronavirus disease (COVID-19), injuries and an increasing incidence of mental health conditions. In 2019, NCDs accounted for 73.6% of deaths worldwide, an increase of 12.8% compared to 2000 (6). Similarly, NCDs are the most important causes of disability-adjusted life years (DALYs) in people older than 50 (7). The COVID-19 pandemic stressed the vulnerability of the ageing population. In the United States (US), individuals aged 85 years and above had an average death rate that was 350 times greater and an average hospitalization rate that was 15 times greater than individuals aged 18 to 29 years (8). Similarly, injuries cause high mortality, morbidity and disability in the ageing population (9–11), and 2022 reports suggest that 20% of older adults meet the criteria for a mental health diagnosis (12). Recognising the growing and unmet needs of the ageing population, the United Nations (UN) and the World Health Organization (WHO) have declared 2021–2030 the “Decade of Healthy Ageing (hereafter referred to as the Decade)” (13). Furthermore, the WHA has recognised the role of rehabilitation in the effective implementation of the Global Strategy and Action Plan on Ageing and Health 2016–2020 (5, 14).

Healthy ageing has been defined as “the process of developing and maintaining the functional ability that enables well-being in older age” (15), and have 2 components: intrinsic capacity—understood as all the mental and physical capabilities of an individual—as well as the functional ability—the outcome of the interaction between Intrinsic capacity (IC) and the built, social, attitudinal and political context of a person (15). Functioning—as introduced by WHO in the International Classification of functioning Disability and Health (ICF)—is the outcome of complex interactions between the health state of an individual and the physical, interpersonal, and social environment (16, 17). From a conceptual perspective, “functional ability” and “functioning” are equivalent concepts and can be used interchangeably. Optimizing “functional ability” or “functioning” is rehabilitation's main objective (18).

Rehabilitation—“a set of interventions designed to optimise functioning and reduce disability in individuals with health conditions in interaction with their environment” (19)—is, for three main reasons, an essential health strategy for addressing the most important challenges raised in the Decade (20, 21). Firstly, rehabilitation aims to improve functioning, which is critical for achieving the Decade's goals of maintaining the ability to perform daily activities, preserving social participation, and meaningful life roles as people age (22). Secondly, rehabilitation considers the individual and contextual factors that affect people's functioning, including the physical, social, and attitudinal environment they live in, as well as their personal characteristics (16). This approach aligns with the Decade's goal of building supportive environments. Thirdly, rehabilitation places the individual at the centre of the care process, considering their unique needs, preferences, and goals (23), in line with the Decade's objective of providing integrated and person-centred care that addresses older adults' physical, mental, and social needs (22). Evidence shows that when integrated into health systems, rehabilitation effectively reduces morbidity and mortality (24, 25), improves functioning/functional ability (26–30), and prolongs independent living (25, 31). Therefore, strengthening rehabilitation services for older adults should be a key priority for policymakers and stakeholders involved in achieving the Decade's goals.

Although rehabilitation's importance for healthy ageing is recognised in the WHO baseline report (22) and now in the WHA assembly resolution (5), it has not yet lived up to its potential in the Decade's agenda. A possible reason might be the lack of concrete guidance for health professionals and policymakers. Open questions that represent a gap in current literature include: which rehabilitation services are the most relevant to ageing populations, how should they be delivered or who could benefit from them? Understanding how rehabilitation is offered to older adults is an essential starting point toward more responsive rehabilitation services.

The objective of this scoping review was to provide a systematic overview of rehabilitation delivery models used to optimise the intrinsic capacity and functioning/functional ability of older adults. The following research question, using the PICO framework, was formulated: What is known from the scientific literature about how rehabilitation is delivered to optimise the intrinsic capacity and functioning/functional ability of older adults? The review will provide rehabilitation stakeholders and policymakers seeking to increase the responsiveness of health systems to ageing populations' growing rehabilitation needs with the information needed to (re)design rehabilitation provision to foster healthy ageing.

## 2 Methods

### 2.1 Study design

We conducted a scoping review to provide a systematic overview of rehabilitation delivery models used to optimise the ageing population's intrinsic capacity and functioning/functional ability. A scoping review was carried out because it is the most appropriate method for examining emerging areas of knowledge, clarifying key concepts and identifying research gaps (32, 33). We followed state-of-the-art methods (33), and used the Preferred Reporting Items for Systematic Reviews and Meta-Analyses extension for Scoping Reviews (PRISMA-ScR) (34). Checklist and study protocol are available in Supplementary files 1 and 2, respectively.

### 2.2 Eligibility criteria

• **Population:** A “study population's mean age higher than 50” was used to identify the “ageing population.” This decision was based on evidence showing that countries with similar levels of age-related burden experience different onsets of ageing, with the lowest starting around 50 years (35). If the full-text paper did not report the mean population age, we included studies targeting diseases with incidence rates among the adult population increasing quadratically with age (35). We used a list of diseases from selected clusters defined elsewhere (36).
• **Intervention:** We included studies describing or testing approaches to provide rehabilitation. They must have had a sufficiently detailed description of the rehabilitation interventions. We excluded papers focusing on describing needs, functional patterns, disability, risk factors, on testing the effect of a single intervention (e.g., botulinum toxin for spasticity), and studies without rehabilitation interventions.
• **Outcome:** Studies aiming to enhance intrinsic capacity, functioning/functional ability [e.g., independence in activities of daily living (ADL), mobility or muscle strength, or social participation] were included. Studies focusing on morbidity, mortality, disease control or intervention adherence were excluded.
• **Publication Type:** Reports resulting from primary research, excluding case reports and case series, were included. Books, reviews, position papers, guidelines, or research protocols were excluded.
• **Setting:** We did not limit study eligibility to any geographical location or level of care.
• **Language and publication date:** We searched for papers published in English after the launch of the WRAH in 2015 (15), and up to May 2022.

### 2.3 Information sources and search strategy

MEDLINE and EMBASE were systematically searched. We considered evidence about the optimal database combination for conducting scoping reviews for this selection (37). Four authors (VS, RM, CS and RB) developed the structured search strategy, which included natural language and Medical Subject Headings, grouped into three concepts, (1) rehabilitation, intrinsic capacity, functioning/functional ability, (2) models of care or health care approaches, and (3) ageing population.

The final search strategy for both databases, including terms and filters applied can be found in additional file 3. The search results were exported into EndNote, where duplicates were removed. We complemented the search by scanning reference lists of systematic reviews identified during the title and abstract screening process. We anticipated an extensive body of evidence to fulfil our study's objective in indexed databases; consequently, we did not search for grey literature.

### 2.4 Study selection process

Two researchers (RM, VS) and a student assistant independently screened abstracts using Rayyan; 50% of the records were double-screened (38). To ensure consistency in the decision process, we held training sessions and team meetings to clarify eligibility criteria and discuss open issues; three training rounds were required to reach at least 90% of agreement. Subsequently, three researchers (RM, PF and VS) and a student assistant assessed independently and in duplicate the full text of retained records for eligibility. Consensus and discussion with a third team member were used to solve disagreements on study selection and data extraction.

### 2.5 Quality assessment of studies

We did not appraise methodological quality or risk of bias, in line with scoping reviews' methodology (33, 39), and with our goal of identifying and describing rehabilitation delivery models rather than assessing if the interventions or strategies used were effective.

### 2.6 Data extraction process

We used four conceptual frameworks (40–43), described in the data synthesis section below, and input from rehabilitation and health systems research experts to develop the data-charting form, which included information regarding studies' characteristics, target population, rehabilitation service delivery and rehabilitation interventions (see additional file 2). The data extraction process began only after a high agreement (>90%) was reached during the training sessions. Three researchers (RM, PF and VS) and a student assistant extracted data independently and in duplicate.

### 2.7 Data synthesis

The synthesis included descriptive quantitative analysis (e.g., frequencies) of study characteristics, rehabilitation interventions, and rehabilitation services provision, and qualitative analysis to identify rehabilitation delivery models.

To achieve a standard categorisation of rehabilitation interventions, we used the International Classification of Health Interventions (ICHI) (40), and the WHO packages of Interventions for rehabilitation (PIR) (41) included in the Universal Health Coverage Compendium (UHC). The term “rehabilitation intervention” corresponds to the “action” level in the ICHI and UHC taxonomies. Based on these frameworks, we predefined six rehabilitation intervention categories: assessment, pharmacological agents, restorative and compensatory approaches, provision of assistive technology (AT), environmental adaptations (EAs), and education and advice. Finally, we used the International Classification of Service Organization in Rehabilitation (ICSO-R 2.0) (42), the Effective Practice and Organisation of Care taxonomy of Health Systems Interventions (43) and WHO's definition of “model of care” (44) to identify and describe rehabilitation delivery models. The International Classification of Functioning Disability and Health (ICF)' linking rules (45) were used to categorise study outcomes.

We classified the level of care into primary health care (PHC), specialised care, or a combination of the two. The level of care was classified as PHC if articles self-identified as PHC, if rehabilitation interventions were provided exclusively by PHC workers, including nurses or general practitioners in a traditional PHC setting (home or community), or if the interventions provided did not require complex equipment or specialised training. Otherwise, studies were classified as specialised care, including secondary or tertiary level. Those that looked at both settings, for example, services starting at a university hospital but continued with a community exercise programme, were classified as a combination of both levels. Co-authors were regularly consulted to validate the categorisation of data.

### 2.8 Patient and public involvement

We did not include older adults, patients or patients' representatives in the scoping reviews' methodological design, conduct, reporting, or dissemination plan.

## 3 Results

### 3.1 Study selection and characteristics

**Figure 1:**
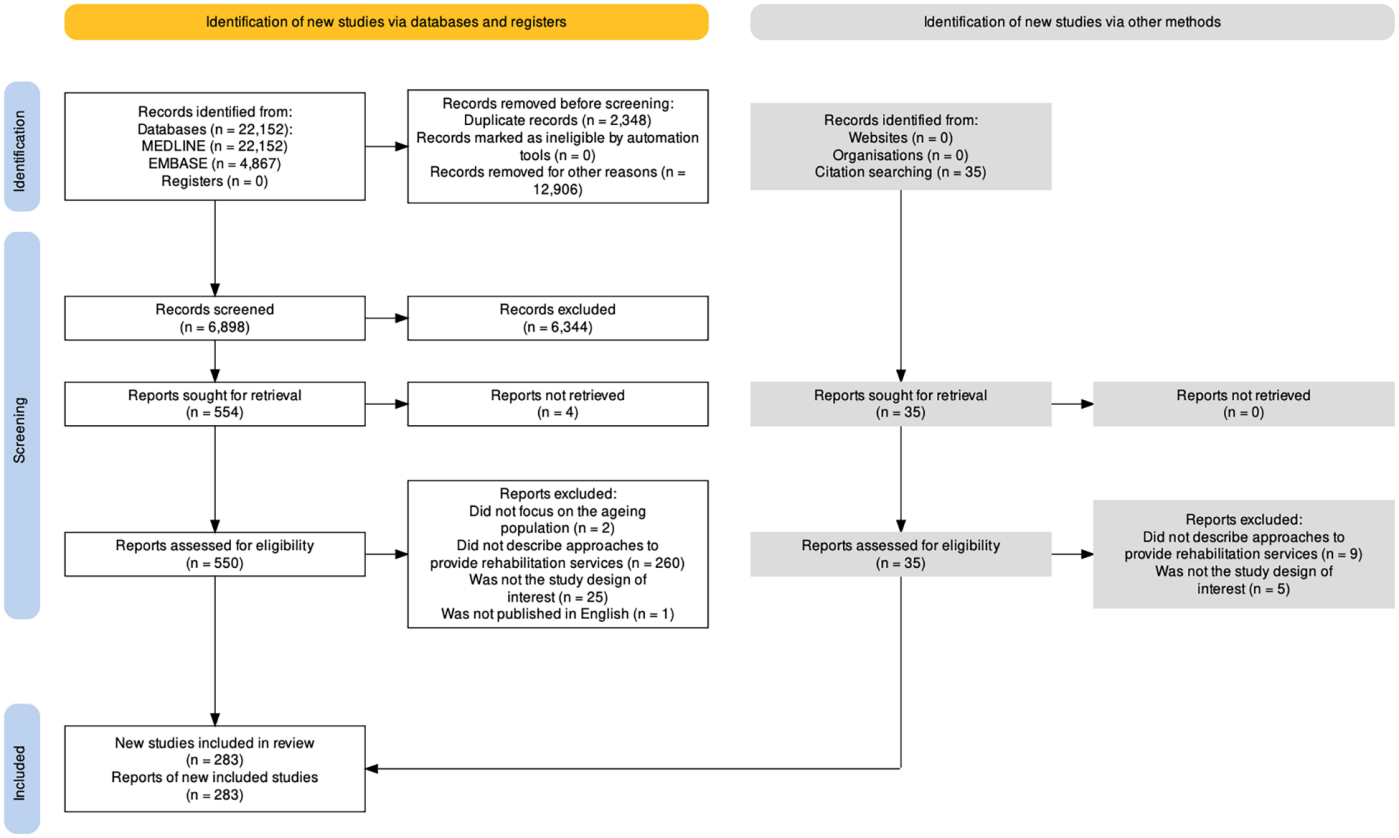
Preferred reporting items for systematic reviews and meta-analyses extension for scoping reviews diagram. (46, 47). Results of the search of May 31, 2022. *Excluded by random sampling of 65%.

Figure 1 shows the PRISMA flow diagram, and Table 1 the most important characteristics of the 283 included studies (see more detail in additional files 4 and 5). Half of the studies were published in 6 countries: the USA, the Netherlands, Australia, China, the UK, and Korea. More than 85% of studies originated from high-income countries (HICs), 12% from upper-middle-income countries, 2% from lower-middle-income countries (LMICs), and only one in a low-income country (LICs), Uganda. Only 40 studies (14.1%) explicitly stated to foster healthy ageing, and 29 were published in 2021. We identified 1,250 study outcome measures but selected and classified 769 relevant to our study's objective. We report in Table 2 the most frequent ones by categories and frequency of use. The most frequently reported outcome type was health-related quality of life (HRQOL). Other outcome types were infrequent and not included in the table; for example, only 6.4% of the included studies measured the risk of falls, 4.6% well-being and 4.2% participation.

**Table 1:** Most important characteristics of the included studies.

Category	Details	*N* (%)*
Year of publication
	2015	37 (13.1)
	2016	42 (14.8)
	2017	23 (8.1)
	2018	47 (16.6)
	2019	17 (6.0)
	2020	28 (9.9)
	2021	67 (23.7)
	2022	22 (7.7)
Country
	United States of America	39 (13.3)
	The Netherlands	30 (10.2)
	Australia	28 (9.6)
	China	24 (8.2)
	United Kingdom	16 (5.5)
	Korea	14 (4.8)
	Spain	13 (4.4)
	Denmark	11 (3.8)
	Sweden	11 (3.8)
	Canada	10 (3.4)
	Italy	10 (3.4)
	Taiwan	10 (3.4)
	Germany	8 (2.7)
	Japan	8 (2.7)
	Norway	8 (2.7)
	Belgium	6 (2.1)
	Turkey	5 (1.7)
	Others**	34 (15.4)
	Total countries	293 (100)
Study design
	Intervention study	230 (81.3)
	Observational study	36 (12.7)
	Descriptive study	17 (6.0)
Age related inclusion criteria
	Not reported	97 (34.3)
	Older than 65	62 (21.9)
	Older than 18	38 (13.4)
	Another criterion***	26 (9.2)
	Older than 60	23 (8.1)
	Older than 75	8 (2.8)
	Older than 70	7 (2.5)
	Older than 50	5 (1.7)
	Ceiling age at 85	6 (2.1)
	Ceiling age at 80	5 (1.7)
	Ceiling age at 70	3 (1.1)
	Ceiling age at 75	2(0.7)
	Ceiling age at 90	1(0.4)

* Represents the proportion of included studies.

** Finland, New Zealand, Portugal Chile, France, Mexico, Brazil, Greece, Iran, Singapore, Austria, Bangladesh, Croatia, Cyprus, India, Ireland, Israel, Malaysia, Nepal, Philippines, Poland, Switzerland, Uganda.

*** Refers to infrequent criteria like “between 40 and 67 years" (48) or population-specific criteria. For example, “older 50 or 55 years” for Aboriginal and Torres Strait Islander people (49, 50) or “older than 60 years” in the Netherlands but “older than 80 years” in Switzerland (51); or comorbidities-adjusted age inclusion related criteria like “older than 65 years” in general but between 50 and 64 for people with a chronic health condition (52).

**Table 2:** Most frequent outcome measurements.

Outcome category	Outcome measurement	*N* (%)
**Health-related quality of life (HRQOL)**		**131** **(46.3)**
	European Quality of Life scale (EQ-5D-5L)	45 (16)
	Short Form Health Survey (SF-36 or SF-12)	43 (15.2)
	World Health Organization Quality of Life questionnaire (WHOQOL)	12 (4.2)
	Minnesota Living with Heart Failure Questionnaire (MLWHFQ)	6 (2.1)
**Independence in activities of daily living**		**108** **(38.2)**
	Barthel Index (BI)	47 (16.6)
	Katz Activities of Daily Living Index	17 (6)
	Lawton and Brody Instrumental Activities of Daily Living Scale (IADL)	17 (6)
	Canadian Occupational Performance Measure (COPM)	7 (2.5)
**Movement functions (b750-b789** ^a^ **)**		**85** **(30)**
	Timed Up and Go (TUG)	37 (13.1)
	Short Physical Performance Battery (SPPB)	23 (8.1)
	30 Second Sit to Stand Test (30CST)	12 (4.2)
	Berg Balance Scale (BBS)	12 (4.2)
**Functioning/functional ability**		**66** **(23.3)**
	Functional Independence Measure (FIM)	12 (4.2)
	Disabilities of the Arm, Shoulder and Hand (DASH)	6 (2.1)
	Fugl Meyer Assessment for the upper extremity (FMA-UE)	6 (2.1)
	World Health Organization Disability Assessment Schedule-WHODAS 2.0	4 (1.4)
**Exercise tolerance functions (b455** ^a^ **)**		**65** **(23)**
	6 Minute Walk Test (6MWT)	29 (10.2)
	Frequency of physical activity	12 (4.2)
	Oxygen uptake (VO2)	9 (3.2)
	2 Minute Walk Test (2MWT)	4 (1.4)
**Mental functions (b1** ^a^ **)**		**56** **(19.8)**
	Mini-Mental State Examination (MMSE)	27 (9.5)
	Montreal Cognitive Assessment (MoCA)	16 (5.7)
	Alzheimer’s Disease Assessment Scale (ADAS)	3 (1.1)
	Confusion Assessment Method (CAM)	3 (1.1)
**Emotional functions (b152** ^a^ **)**		**55** **(19.4)**
	Hospital Anxiety and Depression Scale (HADS)	22 (7.7)
	Geriatric Depression Scale (GDS)	19 (6.7)
	Patient Health Questionnaire (PHQ-9)	10 (3.5)
	Beck Depression Inventory (BDI)	3 (1.1)
**Self-care (d5** ^a^ **)**		**43** **(15.2)**
	General Self Efficacy Scale (GSES)	6 (2.1)
	Health Education Impact Questionnaire (heiQ)	5 (1.8)
	Stroke Self-Efficacy Questionnaire (SSEQ)	5 (1.8)
	Patient Activation Measurement (PAM)	3 (1.1)
**Muscle functions (b730–b749** ^a^ **)**		**36** **(12.7)**
	Handgrip strength	19 (6.7)
	30 Second Sit to Stand Test (30CST)	15 (5.3)
	Western Ontario and McMaster Universities Osteoarthritis Index (WOMAC)	6 (2.1)
	The Arm Curl	3 (1.1)
**Sensation of pain (b280** ^a^ **)**		**21** **(7.4)**
	Western Ontario and McMaster Universities Osteoarthritis Index (WOMAC)	6 (2.1)
	Visual Analogue Scale (VAS)	3 (1.1)
	Brief Pain Inventory (BPI)	2 (0.7)
	Knee Injury and Osteoarthritis Outcome	2 (0.7)

^a^ Terminology and code of the International Classification of Functioning Disability and Health (ICF).

### 3.2 Rehabilitation programmes’ beneficiaries

Table 3 shows the most important characteristics of rehabilitation beneficiaries, who were on average 70.6 years old. Six studies included people with a mean age higher than 85, two from Japan (53, 54). Women were the predominant population in 43.1% of the studies, and eight studies only included women. More than half of the studies selected the participants based on the presence of a single health condition (59%). Neurological conditions were most frequent. People with a decline in functioning/functional ability, which included non-specified declines in functioning/functional ability, fragility, or sarcopenia, accounted for 35% of the participants. Other populations like people with pain, low vision and hearing, and urinary dysfunctions were uncommonly addressed. Four papers were about COVID-19 rehabilitation (55–58).

**Table 3:** Most important characteristics of rehabilitation programmes’ beneficiaries.

Characteristics	Details	*N* (%)*
Participants’ sex predominance
	Female predominance	122 (43.1)
	Balanced	90 (31.8)
	Male predominance	55 (19.4)
	Not reported	16 (5.7)
Target population
	People with a single health condition	167 (59.0)
	People with a decline in functioning/functional ability**	99 (34.9)
	People with more than two health conditions	17 (6.0)
Health condition area
	Neurological	59 (33.9)
	Cardiovascular	39 (22.4)
	Musculoskeletal	34 (19.5)
	Respiratory	16 (9.2)
	Cancer	7 (4.0)
	Metabolic	6 (3.5)
	Communicable diseases	5 (2.9)
	Pain	3 (1.7)
	Autoimmune diseases	2 (1.2)
	Sensory	2 (1.2)
	Urological	1 (0.6)
Health condition
	Stroke	41 (23.3)
	Chronic obstructive pulmonary disease	16 (9.1)
	Hip fracture or post hip arthroplasty	16 (9.1)
	Heart failure	13 (7.4)
	Coronary heart disease	12 (6.8)
	Cardiovascular disease not specified	11 (6.3)
	Cognitive impairment	11 (6.3)
	Osteoarthritis	10 (5.7)
	Breast cancer	4 (2.3)
	COVID-19	4 (2.3)
	Diabetes	4 (2.3)
	Others***	34 (19.4)

* Represents the proportion of included studies.

** Parkinson’s disease, Multiple Sclerosis, other neurological diseases not specified, Peripheral Artery Disease, chronic pain, Complex Regional Pain Syndrome, degenerative scoliosis, low back pain, rotator cuff tendinopathy, lower limb amputation, distal radius fracture, other fractures or injuries not specified, prostate cancer, lung cancer, lymphoma, other cancers not specified, sarcopenia, chronic kidney disease, Diffuse Cutaneous Systemic Sclerosis, inflammatory arthritis, vestibular dysfunction, vision impairment and, Human Immunodeficiency Virus. COVID-19: Coronavirus disease 2019.

*** Include those with a unspecified decline in functioning/functional ability, fragility, or sarcopenia.

### 3.3 Rehabilitation service delivery

Table 4 shows the most important service delivery characteristics. Rehabilitation was provided across levels of care through six “Mode of service delivery”: outpatient (30.7%), telerehabilitation (28.3%), home (23.7%), community (20.9%), inpatient (18.7%), and eldercare (6%). “Mode service delivery”, is defined by the ICSO-R 2.0 as “*the way services are delivered to the users. Inclusions: Inpatients, outpatients, day hospital/day service, home and community, tele-rehabilitation, or any other setting for service delivery*” (42). Most studies employed one (75.2%) or two (21.2%) delivery modes to provide rehabilitation services, with one study using four (59). These “*Modes of service delivery*” emerged during data synthesis as natural categories for organising and describing rehabilitation models. We will use the term “*Rehabilitation delivery models*” hereafter. For more information about the models, see below Section 3.5.

**Table 4:** Service delivery characteristics and rehabilitation interventions.

Characteristics	Details	*N* (%)*
Level of care	Specialised health care	133 (47.0)
	Primary health care	97 (34.3)
	Multiple levels of care	53 (18.7)
Service provider	Health workers	252 (93.7)
	Informal caregivers and family	18 (6.7)
	Peers and volunteers	10 (3.7)
	Not reported	12 (4.6)
Health workers
	Physical therapist	130 (45.9)
	Nurse	112 (39.6)
	Occupational therapist	77 (27.2)
	Other physicians**	40 (14.1)
	Psychologist	31 (11)
	Social worker	31 (11)
	Dietician	28 (9.9)
	General practitioner	27 (9.5)
	Others***	27 (9.5)
	Geriatrician	21 (7.4)
	Rehabilitation physicians	15 (5.3)
	Speech and language therapist	12 (4.2)
	Exercise professionals	10 (3.5)
	Community workers	9 (3.2)

* Represents the proportion of included studies.

** Neurologists, psychiatrists, cardiologists, neurosurgeons, radiologists, orthopaedic surgeons, and other physicians not specified.

*** Pharmacists, podiatrists, dance instructors, rehabilitation scientists, optometrists, home care assistants, music therapists, therapist assistants or students, gerontologists, researchers, health and welfare officers and multidisciplinary teams, mental health workers or therapists not specified.

Health workers provided rehabilitation interventions in 93.7% of the studies. Excluding prosthetists and orthotists, all traditional rehabilitation workers were somewhat represented. The most frequent providers were physical therapists, present in 45.9% of studies. Multidisciplinary teams provided rehabilitation in 31.5% of studies. Role or task shifting or sharing was described in 13.4% of the studies, mainly by physical or occupational therapists towards nurses (60, 61), community workers (62) or informal caregivers (63). More than 30% of papers provided rehabilitation following integrated care principles (64), 35.7% assessed person-centred goals, and 47% provided specialised care.

### 3.4 Rehabilitation interventions

We identified 1,361 rehabilitation interventions (Table 5). Although we predefined six categories, during the data analysis another category of rehabilitation interventions emerged: “coordination and management of the rehabilitation process”. This category accounted for 20% of all interventions provided and was used in more than half of the studies. Restorative and compensatory approaches were the most frequently used interventions. In contrast, interventions such as provision and training in the use of AT (8.1%), EAs (7.4%), and pharmacological agents (3.2%) were rarely reported.

**Table 5:** Rehabilitation interventions.

Intervention category	Intervention for rehabilitation^a^	*N* (%)
**Assessments**		**172** **(60.8)**
	Person-centred goals	113 (39.9)
	Functioning/functional ability (overall)	67 (23.7)
	Environment	25 (8.8)
	Health status	20 (7.1)
	Fall risk	19 (6.7)
	Emotional functions	18 (6.4)
	Comprehensive geriatric assessment	18 (6.4)
	Medication used	15 (5.3)
	Cognitive functions	9 (3.2)
	Exercise capacity	5 (1.8)
	Nutritional status	5 (1.8)
	Independence on activities of daily living	4 (1.4)
	Family and caregivers support network	3 (1.1)
**Restorative and compensatory approaches**		**240** **(84.8)**
	Therapeutic exercise	153 (54.1)
	Multicomponent care or rehabilitation program not specified	54 (19.1)
	ADL training	40 (14.1)
	Motivational interventions	28 (9.9)
	Psychological interventions not specified	28 (9.9)
	Cognitive rehabilitation	25 (8.8)
	Occupational therapy not specified	22 (7.8)
	Behavioural interventions	20 (7.1)
	Therapeutic recreation	11 (3.9)
	Physical therapy not specified	9 (3.2)
	Problem solving skills training	8 (2.8)
	Speech and language therapy not specified	7 (2.5)
	Management of incontinence	4 (1.4)
	Manual therapy	4 (1.4)
	Social skills training	3 (1.1)
	Thermal modalities	3 (1.1)
	Others^b^	5 (2)
**Education and counselling**		**156** **(55.1)**
	Education and skills training for selfcare and self-management	116 (41.0)
	Education and skills training for caregivers	40 (14.1)
	Education and counselling for physical activity and therapeutic exercise	30 (10.6)
	Education and counselling about healthy diet and nutritional requirements	25 (8.8)
	Education and counselling to modify harmful lifestyle behaviours	22 (7.8)
	Education and counselling for weight management	3 (1.1)
**Coordination and management of the rehabilitation process**		**155** **(54.8)**
	Follow up visits	72 (25.4)
	Case management	45 (15.4)
	Health status monitoring	44 (15.5)
	Rehabilitation process coordination and management	38 (13.4)
	Discharge planning	35 (12.4)
	Monitoring of functional ability	35 (12.4)
	Home visits	11 (3.9)
**Provision and training in the use of assistive technology**		**23** **(8.1)**
**Environmental adaptations**		**21** **(7.4)**
**Pharmacological agents**		**9** **(3.2)**

^a^ Rehabilitation intervention corresponds to the “action” level in the Universal Health Compendium taxonomy of interventions.

^b^ Electromyography-triggered neuromuscular stimulation, low vision rehabilitation, music therapy, swallowing therapy and wound care. ADL, activities of daily living.

We found that therapeutic exercises (54.1%), education and skills training for self-care and self-management (41%), and assessment of person-centred goals and priorities (40%) were the most frequently utilised rehabilitation interventions and were commonly prescribed together. Examples of therapeutic exercises included muscle strengthening and balance training, functional exercises such as training in ADLs, aerobic exercise, and cognitive training. Furthermore, social care and support interventions such as assistance in ADLs and emotional support were provided in 16% of the studies in addition to rehabilitation.

We also sought to determine whether the rehabilitation programmes included some evaluation of functioning/functional ability or intrinsic capacity. To that end, we grouped the following assessments: Assessment of overall functioning/functional ability, health status, emotional functions, cognitive functions, exercise capacity, independence in activities of daily living and the Comprehensive Geriatric Assessment. Overall, 36.8% of studies used one of these measurements.

### 3.5 Rehabilitation delivery models

**Figure 2:**
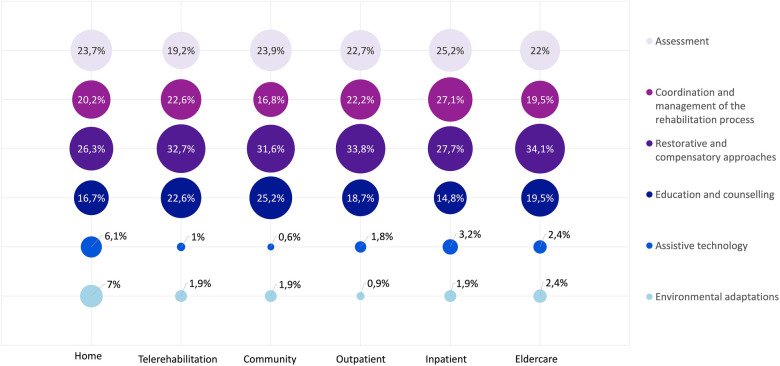
Type of rehabilitation intervention category by delivery model. Percentages represent the proportion of interventions by category over the number of interventions per model.

We use the term “Rehabilitation delivery model” to describe the approach by which rehabilitation services were provided to the person. As explained before, the “*Modes of service delivery”* of the ICSO-R 2.0 classification emerged as the natural categories for organising and describing rehabilitation delivery models during data synthesis. Below we present a description of each model, based on a quantitative analysis of key characteristics. More information can be found in Table 6 and Figure 2.

**Table 6:** Models for rehabilitation service delivery.

Mode	Studies using this model, *N* (%)	Most frequent countries (%)	Mean age (SD)	Studies' sex predominance (%)^a^	Most frequent health condition areas (%)	Most frequent health conditions (%)	Multimorbidity, *N* (%)^b^	Decline in functional ability, *N* (%)^c^
Outpatient	87 (30.7)	Netherlands (16.3) USA (9.8) Australia (8.7) China (6.5)	69.2 (9.3)	Females (39.1) Balanced (33.3) Males (24.1)	Neurological (33.9) Cardiovascular (20.3) Musculoskeletal (20.3)	Stroke (19.7) Coronary heart disease (9.8) Cognitive impairment (8.2) COPD (6.6)	6 (6.9)	25 (28.7)
Telerehabilitation	80 (28.3)	USA (20) Australia (9.4) China (7.1) Italy (7.1)	66.6 (8.4)	Balanced (33.8) Females (32.5) Males (32.5)	Cardiovascular (37.5) Neurological (23.4) Musculoskeletal (15.6)	Heart failure (16.7) COPD (10.6) Cardiovascular disease not specified (9.1) Coronary heart disease (9.1)	1 (1.3)	16 (20.0)
Home	67 (23.7)	Australia (11.9) China (11.9) Denmark (6) Finland (6)	74.4 (7.8)	Females (56.7) Balanced (22.4) Males (17.9)	Musculoskeletal (38.5) Neurological (38.5) Cardiovascular (12.8)	Stroke (35.9) Hip fracture or post hip arthroplasty (25.6) Osteoarthritis (12.8) Coronary heart disease (10.3)	4 (6.0)	26 (38.8)
Community	59 (20.9)	USA (12.7) China (11.1) UK (11.1) Australia (9.5)	69.6 (8.8)	Females (54.2) Males (8.5)	Neurological (32.1) Balanced (23.7) Respiratory (14.3)	Stroke (28.6) COPD (14.3) Cardiovascular (21.4) Hip fracture or post hip arthroplasty (10.7) Chronic pain (7.1)	5 (8.5)	27 (45.8)
Inpatient	53 (18.7)	USA (18.9) China (17) Netherlands (13.2) Australia (7.5)	74.5 (8.4)	Balanced (39.6) Females (35.9) Males (18.9)	Neurological (50) Musculoskeletal (30.6) Cardiovascular (11.1)	Stroke (44.4) Hip fracture or post hip arthroplasty (22.2) COVID-19 (5.6) Cognitive impairment (5.6)	2 (3.8)	17 (32.1)
Eldercare	17 (6.0)	Japan (17.6) USA (17.6) Australia (11.8) Korea (11.8)	79.8 (5.6)	Females (70.6) Males (29.4)	Neurological (66.7) Metabolic (16.7) Respiratory (16.7)	Cognitive impairment (50) COPD (16.7) Sarcopenia (16.7) Stroke (16.7)	0 (0)	11 (64.7)

Mode	Level of care (%)	Health workers (%)	Multidisciplinary, *N* (%)^f^	Task shifting, *N* (%)^g^	Integrated care, *N* (%)	Most frequent rehabilitation interventions (%)
Outpatient	Primary health care (18.4) Specialized health care (51.7) Levels of care combination (29.9)	Nurse (20.7) Physical therapist (19.7) Occupational therapist (10.8) Psychologist (6.9)	32 (36.8)	9 (10.3)	34 (39.1)	Therapeutic exercise (11.3) Education for selfcare (8.6) Assessment person-centred goals (8.4) Follow up (6.7) Assessment functional ability (4.9) Case management (3.9) Multicomponent rehabilitation (3.2)
Telerehabilitation	Primary health care (17.5) Specialized health care (72.5) Levels of care combination (10)	Nurse (22.7) Physical therapist (19.5) Occupational therapist (9.4) Other physicians (9.4)^d^	11 (13.8)	10 (12.5)	6 (7.5)	Therapeutic exercise (15.2) Education for selfcare (11.2) Monitoring of health status (7.6) Assessment person-centred goals (7.3) Follow up (7) Monitoring of functional ability or intrinsic capacity (3.9) Motivational interventions (3.2)
Home	Primary health care (35.8) Specialized health care (31.3) Levels of care combination (32.8)	Physical therapist (26.2) Nurse (14) Occupational therapist (16.5) Other physicians (6.1)^d^	27 (40)	11 (16.4)	30 (44.8)	Therapeutic exercise (9.3) Assessment person-centred goals (9.1) Follow up (6.2) Assessment functional ability (5.7) Education for selfcare (5.7) Multicomponent rehabilitation (3.9) Training on activities of daily living (3.2)
Community	Primary health care (81.4) Specialized health care (8.5) Levels of care combination (10.2)	Physical therapist (20.4) Nurse (15) Occupational therapist (9.7) General practitioner (7.1)	15 (25.4)	9 (15.3)	16 (27.1)	Therapeutic exercise (12.8) Education for selfcare (12.5) Assessment person-centred goals (8.4) Follow up (5.9) Assessment functional ability (5.1) Case management (3.3) Education for self-directed therapeutic exercise (3.3)
Inpatient	Specialized health care (47.2) Levels of care combination (52.8)	Nurse (20.8) Physical therapist (19.5) Occupational therapist (15.7) Other physicians (8.2)^d^	28 (52.8)	8 (15.1)	39 (73.6)	Discharge planning (10) Assessment person-centred goals (8) Multicomponent rehabilitation (7.7) Assessment functional ability (6.4) Follow up (6.4) Follow up (6.4) Therapeutic exercise (3.9) Rehabilitation planning and coordination (3.2)
Eldercare	Primary health care (35.3) Specialized health care (47.1) Levels of care combination (17.7)	Physical therapist (21.6) Nurse (18.9) Occupational therapist (10.8) Other (10.8)^e^	3 (0.83)	3 (17.6)	6 (35.3)	Therapeutic exercise (15.9) Assessment person-centred goals (11.1) Education for caregivers (7.9) Cognitive rehabilitation (6.3) Assessment functional ability (4.8) Case management (4.8) Multicomponent care or rehabilitation (4.8)

^a^ Females: Females accounted for more than 60% of the study participants. Males: Males accounted for more than 60% of the study participants. Balanced: Neither females nor males represented more than 60% of the study population.

^b^ Studies in which the target population was people with multimorbidity.

^c^ Studies in which the target population was people with a decline in functional ability, functioning, fragility, or sarcopenia. COPD, chronic obstructive pulmonary disease; COVID-19, coronavirus disease 2019; USA, United States of America; UK, United Kingdom.

^d^ Other physicians included, neurologists, psychiatrists, cardiologists, neurosurgeons, radiologists, orthopaedic surgeons, and other physicians not specified.

^e^ Other health workers included, pharmacists, podiatrists, dance instructors, rehabilitation scientists, optometrists, home care assistants, music therapists, therapist assistants or students, gerontologists, researchers, health and welfare officers and multidisciplinary teams, mental health workers or therapists not specified.

^f^ Multidisciplinary team defined as more than 3 health workers.

^g^ Included role or task shifting or sharing.

#### 3.5.1 Outpatient rehabilitation

In this model, the person attended a healthcare facility to receive rehabilitation and came back to their place of origin. More than half of the studies, 51.7%, took place at specialised levels of care and only 18.4% at PHC. Outpatient rehabilitation focused on people with a mean age of 69.2, women (39.1%) and neurological conditions (33.9%), out of which stroke was the most frequent (19.7%). Besides the inpatient model, this was the only other model in which cognitive impairment was a frequent target (8.2% of the studies) and the only one in which psychologists were one of the most frequent rehabilitation providers. Multidisciplinary teams and integrated care principles were used in almost 40% of studies. Only 4 out of the 225 provided interventions included AT (65–68). An exemplary study tested the effectiveness of a self-management intervention aimed at proactive coping for stroke patients and partners (69). Another evaluated the experiences of community-dwelling older people with dementia participating in a person-centred multidimensional, interdisciplinary rehabilitation programme (65).

#### 3.5.2 Telerehabilitation

Rehabilitation was provided using telecommunication technologies, either synchronously, e.g., live consultation, or asynchronously, e.g., access to information and courses with subsequent feedback and follow-up. Telerehabilitation was also used to monitor participants’ general health status, body functions, activities, or participation. For example, some studies used automatic call systems for the early detection of complications. Others used wearable devices to monitor physiological variables, changes in walking distance, or self-care behaviours. Although considered an outreach health strategy, sometimes classified as PHC, 72.5% of the services provided were specialised. It focused on a younger population (mean age 66.6), both men and women, with cardiorespiratory health conditions, including heart failure, COPD, and coronary heart disease. It was the model with the lowest frequency of papers targeting decline in functioning/functional ability and using integrated care principles. An exemplary study used telerehabilitation to ensure the continuity of care at home for patients with chronic neurological diseases (70), and another used mHealth interventions to improve heart failure self-care and support informal caregivers (71).

#### 3.5.3 Home-based rehabilitation

Rehabilitation was provided at the person's home through PHC (35.8%), specialised care (31.3%) or a combination of both (32.8%). Specialised care at home was characterised by multidisciplinary teams and specialised health care professionals' home visits. It focused on women with a mean age comparable to the inpatient model, 74.4 years. It was the only model where musculoskeletal was the most frequent health condition area, with almost 40% of studies targeting hip fractures, post-hip arthroplasty, or osteoarthritis. Nearly 40% of studies addressed older adults with a decline in functioning/functional ability. It was the second model with the highest frequency of multidisciplinary rehabilitation (40%) and the use of integrated care principles (44.8%). The highest representation of physical (26.2%) and occupational therapists (16.5%) was observed here. It was the only one where training on ADLs was a frequently provided rehabilitation intervention and the one with the highest provision of AT and EAs. Exemplary studies assessed the use of rehabilitation to age at home (59) and a home-based rehabilitation inpatient bed-substitution model (72).

#### 3.5.4 Rehabilitation in the community

Rehabilitation was provided in a community setting, for example, a community centre or a public recreational area. Most of the services offered were not specialised (81.4%), and it was the only model in which general practitioners were frequent rehabilitation providers. It focused on women (54.2%), with a mean age of 69.6, persons with neurological conditions (32.1%), and a general decline in functioning/functional ability (45.8%). We found five papers using the community model to improve functioning in the ageing population with multimorbidity (73–76). Task shifting was used in 15% of studies. It was the only model in which education for self-directed therapeutic exercise was a frequently provided intervention. One paper included the provision of AT (77), and three EA (59, 78, 79). An exemplary study provided rehabilitation interventions in the community to prevent fragility and foster healthy ageing in Japan (80), and two provided rehabilitation after stroke (28, 81).

#### 3.5.5 Inpatient rehabilitation

Rehabilitation was provided during an inpatient episode of care through either specialised (47.2%) or a combination of specialised and PHC (52.8%) care. Inpatient rehabilitation was often followed by outpatient (17 studies) or home-based rehabilitation (14 studies). Studies using this model focussed equally on men and women with a mean age of 74.5. It was the most frequently used model for people with stroke (44.4%), and was characterised by multidisciplinary care (52.8%), task shifting (15%), and the use of integrated care principles (73.6%). It was the only model in which therapeutic exercise was not the most frequently provided intervention. Instead, discharge planning (10%), assessment of person-centred goals (8%) and multicomponent rehabilitation (7.7%) played a prominent role. Discharge planning frequently included social workers coordinating discharge while considering the person's functioning/functional ability, for example, towards a skilled nursing facility, assisted living communities, or the person's home (82). It was the only model where “coordination and management of the rehabilitation process” was one of the most frequent interventions, including eight studies where rehabilitation physicians played a coordinating role (83–90). The provision of AT (five studies) and EAs (three studies) was scarce. An exemplary study tested a novel interdisciplinary rehabilitation programme for hospitalised patients with COVID-19 (57), and another examined the effect of interdisciplinary and comprehensive rehabilitation on physical functioning trajectories after hospitalisation (91).

#### 3.5.6 Eldercare-based rehabilitation

Rehabilitation was provided at eldercare facilities in 4.7% of the studies. Rehabilitation workers were part of the facilities personnel or commuted to deliver the services. Specialised services were frequent (47.1%), mostly due to the provision of rehabilitation by specialised healthcare workers, namely geriatricians. Eldercare-based rehabilitation focused on an older population (mean age 79.8), women (70%) and people with an overall decline in functioning/functional ability (64.7%). It was the only model where cognitive impairments were the most frequent intervention target (50%) and the only one in which cognitive rehabilitation was one of the most frequently provided interventions (6.3%). We observed the highest frequency of task-shifting or sharing to provide rehabilitation interventions (17.6%) in this model. Like the community model, only one study included providing AT and EAs. An exemplary study tested a national programme to stimulate self-organising capacity to develop integrated care to improve geriatric rehabilitation service delivery (92). Another assessed the effects of group-based motor and cognitive-combined intervention on social activity and quality of life (53).

## 4 Discussion

Focusing on primary studies, this scoping review provides an overview of rehabilitation delivery models that are used to foster healthy ageing. The six emerging models are outpatient, telerehabilitation, home, community, inpatient, and eldercare. Rehabilitation interventions to improve intrinsic and functional capacity, the two components of healthy ageing, were provided in all models. These models were used across levels of care (PHC, specialised care or a combination of both) mainly using a disease-centred perspective and frequently focusing on one neurological, musculoskeletal, or cardiovascular condition, with stroke targeted in almost a quarter of all included studies. Multimorbidity and prevalent health problems of the ageing population, like pain, low hearing, and vision, fall prevention, incontinence, or sexual dysfunctions, were infrequent rehabilitation targets. Multidisciplinary teams and principles of integrated care were frequently used to provide rehabilitation. The most common rehabilitation interventions included assessing person-centred goals and delivering therapeutic exercises as well as education and advice on self-care. Assessing overall functioning/functional ability, or intrinsic capacity is the starting point of any rehabilitation process (93), however, only one-third of papers reported its assessment. Similarly, the provision of AT (e.g., orthosis or prosthesis) and EA (e.g., home adaptations for wheelchair accessibility) was rare, although this is one of the key determinants of functioning/functional ability of the ageing population (94, 95), and a core rehabilitation intervention.

WHO Members States and key stakeholders have agreed in the *WHO Rehabilitation 2030 call for action* that defining essential rehabilitation interventions will facilitate the integration of rehabilitation in health systems (96). To answer this call, WHO developed Packages of Interventions for Rehabilitation (PIR), which provide a prioritised set of evidence-based interventions for rehabilitation (97). Inspired by WHÓs work, we identified and classified rehabilitation interventions offered to older adults in line with the PIR. Although we found a broad range of interventions, our results do not fully align with the PIRs (97) and other documents mapping the needs of older adults (22, 98). For example, in the PIR, a prominent role is given to interventions targeting communication, speech, language, sensory, bowel, bladder or sexual functions as well as frequent ageing-related issues like pain or falls and providing pharmacological agents, ATs, and EAs. Conversely, in our review, studies rarely addressed these issues. Although research in AT seems to be a neglected issue in the general (99) and in the older adult population (100), the lack of provision of standard rehabilitation interventions seems to be a characteristic of rehabilitation programmes offered to older adults. To the best of our knowledge, there is only one comprehensive clinical practice guideline addressing the rehabilitation needs of older adults (101). Other guidelines are discipline-based, only addressing occupational or physical therapy and do not describe services' provision organisation or characteristics (102, 103), or include general recommendations for rehabilitation but do not address models for service provision (104). A better match between the rehabilitation needs of older adults and rehabilitation interventions is very much needed and should be defined in specific clinical practice guidelines in line with the Decade's agenda.

Rehabilitation interventions are provided to improve, maintain or slow down the decline in functioning/functional ability and intrinsic capacity by addressing specific impairments in body functions, like muscle weakness or pain, limitations in activities, like walking around, or restrictions in participation, like joining family celebrations, as well as environmental barriers, regardless of the health condition causing them (17). This person- and not disease-centred perspective makes rehabilitation suitable to address complex limitations in functioning, like the ones arising from multimorbidity (105, 106). Multimorbidity, the co-occurrence of two or more chronic conditions (107), is present in around two-thirds of adults older than 60 (108). In contrast, almost 60% of included studies had a single disease-centred perspective, and only 6% addressed multimorbidity. This finding is in line with the challenges faced by WHO during the development of the PIRs: WHO had to follow a disease-centred approach because almost all scientific evidence in rehabilitation was linked to single diseases (109). Perhaps, as a result, a PIR for ageing is not being developed yet, although this would considerably foster the access of the ageing population to rehabilitation, especially in LMICs. Ensuring that rehabilitation remains person-centred is essential for the ageing population, given the prevalence of or age-related impairments, and necessary to contribute to the healthy ageing agenda.

Primary healthcare (PHC) plays a critical role in establishing a sustainable health system that offers universal health coverage (110). Our review suggests, at least from a research perspective, that integrating rehabilitation services into PHC is a feasible approach for providing rehabilitation to the ageing population. Rehabilitation services have been traditionally centred on specialised care, and there are ongoing challenges to its delivery through PHC (111). This is in line with our results showing that almost 50% of rehabilitation services delivered were specialised. However, WHO has called to realign PHC, stressing that “most rehabilitation services can be provided outside hospital settings, in communities or at home (64).” Indeed, our review shows that more than 90% of rehabilitation in the community and almost 80% of home-based is delivered through PHC alone or in combination with specialised care. Models for the provision of rehabilitation in PHC have already been described for the general population, including “clinic, outreach, self-management, community-based, shared care, and case management” (112). A similar in-depth understanding of how rehabilitation is currently delivered through PHC for ageing populations is still needed to guide the efforts required to strengthen PHC to provide rehabilitation.

This review reveals a surprisingly high involvement of health workers not traditionally considered rehabilitation providers. Usual rehabilitation providers are physical, occupational, and speech and language therapists; prosthetists and orthotists, psychologists and physical and rehabilitation medicine doctors (19, 111). However, in this review, nurses, general practitioners, specialised physicians including geriatricians, neurologists and cardiologists, dieticians, social and community workers, and exercise experts were the rehabilitation providers in half of the identified papers, while some traditional rehabilitation workers were relatively scarce. This can be explained by the large number of studies delivered through PHC, where rehabilitation professionals are generally unavailable and where task-shifting plays a crucial role in increasing access to health services (111), especially in low-resource settings (113). Indeed, we frequently observed task-shifting from physical or occupational therapists to nurses. Our findings point toward a new understanding of who is the rehabilitation workforce, especially if the calls for strengthening provision through PHC are followed (22, 96). Perhaps to meet the rehabilitation needs of an ageing population, the rehabilitation workforce shouldn't always be limited to traditional professional groups. However, as pointed out by WHO's rehabilitation competency framework (114), training health workers becomes critical to ensure properly provided interventions and should focus on strengthening the health workforce's rehabilitation competencies.

Rehabilitation's role in the UN and the Decade (13), needs to be strengthened and better specified. Albeit mentioned several times in the WRAH, the role of rehabilitation in the healthy ageing agenda has not been concretely defined yet (22). The results of our review align with a recently created evidence and gap map for health and social support for the ageing population (115), showing that rehabilitation is already one of the most frequent interventions needed by older adults (115). Our detailed description of six rehabilitation delivery models shows that rehabilitation is indeed provided to the ageing population across levels of care, through specialised and PHC, by multidisciplinary teams, following principles of integrated care and assessing person-centred goals. However, our work also points out shortcomings like a frequent disease-centred perspective, the rare focus on multimorbidity and age-related impairments, the seldom provision of AT and EAs and the inconsistent assessment of intrinsic capacity and functioning/functional ability. Our findings can be used by policymakers and key stakeholders to improve the responsiveness of health systems to the needs of ageing populations. However, a more prominent and better-defined role for rehabilitation in the key technical documents and reports of the Decade of Healthy Ageing is needed to provide essential guidance to rehabilitation stakeholders. Greater commitment from policymakers and key stakeholders is needed to unlock the considerable potential of rehabilitation to contribute to the achievement of the goals of the Decade of Healthy Ageing.

### 4.1 Strengths and limitations

We believe this is the first review providing an overview of rehabilitation delivery models used to optimise older adults' intrinsic capacity and functioning/functional ability. We followed standard methods for designing, conducting, and reporting scoping reviews, and included many papers with different study designs and settings. We used five conceptual frameworks to conduct an innovative synthesise, including the UHC and the PIR.

Our review has limitations. First, results are based only on published primary research. Future research conducted in the field including key rehabilitation stakeholders is needed to expand and confirm the validity of our results. Second, we have excluded papers reporting single interventions because rehabilitation is defined as a “set of interventions” (19) and because we did not focus on the interventions' effectiveness. Third, we conducted an extensive search but screened a random sample of 35% of hits. This might have left some relevant papers out. Nevertheless, we consider that the included 283 studies were sufficient to achieve the goal of the review and that further studies would not have significantly changed the identified models. Finally, most studies were conducted in HICs or LMICs, and results cannot be generalised to low-income settings.

## 5 Conclusions

Our study provides a comprehensive overview of six rehabilitation delivery models that can be used to (re)design rehabilitation services to improve the responsiveness of health systems to the needs of older adults. We also identified key gaps in rehabilitation provision, such as the unsystematic assessment of functioning/functional ability or the lack of provision and training in the use of AT and EA. Rehabilitation can make a meaningful contribution to achieving healthy ageing, but a more prominent and better-defined role for rehabilitation in the key technical documents and reports of the United Nations Decade of Healthy Ageing is urgently needed.
