# Supplementary material for: Rehabilitation delivery models to foster healthy ageing—a scoping review
Source: Front Rehabil Sci. 2024 Apr 5;5:1307536. doi: 10.3389/fresc.2024.1307536 (PMC11041397; doi:10.3389/fresc.2024.1307536)
Supplement: Supplementary file 1 [file Datasheet1.pdf]

Supplementary Material

Supplementary Material 1

Preferred Reporting Items for Systematic reviews and Meta-Analyses extension for Scoping Reviews (PRISMA-ScR) Checklist

SECTION	ITEM	PRISMA-ScR CHECKLIST ITEM	REPORTED ON PAGE #
**TITLE**			
Title	1	Identify the report as a scoping review.	1
**ABSTRACT**			
Structured summary	2	Provide a structured summary that includes (as applicable): background, objectives, eligibility criteria, sources of evidence, charting methods, results, and conclusions that relate to the review questions and objectives.	1
**INTRODUCTION**			
Rationale	3	Describe the rationale for the review in the context of what is already known. Explain why the review questions/objectives lend themselves to a scoping review approach.	2 to 3
Objectives	4	Provide an explicit statement of the questions and objectives being addressed with reference to their key elements (e.g., population or participants, concepts, and context) or other relevant key elements used to conceptualize the review questions and/or objectives.	3
**METHODS**			
Protocol and registration	5	Indicate whether a review protocol exists; state if and where it can be accessed (e.g., a Web address); and if available, provide registration information, including the registration number.	3 and Supplementary Material 2
Eligibility criteria	6	Specify characteristics of the sources of evidence used as eligibility criteria (e.g., years considered, language, and publication status), and provide a rationale.	3
Information sources*	7	Describe all information sources in the search (e.g., databases with dates of coverage and contact with authors to identify additional sources), as well as the date the most recent search was executed.	3 and Supplementary Material 3 and PRISMA diagram (Figure 1)
Search	8	Present the full electronic search strategy for at least 1 database, including any limits used, such that it could be repeated.	3 and Supplementary Material 3
Selection of sources of evidence†	9	State the process for selecting sources of evidence (i.e., screening and eligibility) included in the scoping review.	3 to 4
Data charting process‡	10	Describe the methods of charting data from the included sources of evidence (e.g., calibrated forms or forms that have been tested by the team before their use, and whether data charting	4 and Supplementary Material 2

SECTION	ITEM	PRISMA-ScR CHECKLIST ITEM	REPORTED ON PAGE #
		was done independently or in duplicate) and any processes for obtaining and confirming data from investigators.	
Data items	11	List and define all variables for which data were sought and any assumptions and simplifications made.	4 and Supplementary Material 2
Critical appraisal of individual sources of evidence§	12	If done, provide a rationale for conducting a critical appraisal of included sources of evidence; describe the methods used and how this information was used in any data synthesis (if appropriate).	4
Synthesis of results	13	Describe the methods of handling and summarizing the data that were charted.	4 and Supplementary Material 2
**RESULTS**			
Selection of sources of evidence	14	Give numbers of sources of evidence screened, assessed for eligibility, and included in the review, with reasons for exclusions at each stage, ideally using a flow diagram.	4 and PRISMA diagram (Figure 1)
Characteristics of sources of evidence	15	For each source of evidence, present characteristics for which data were charted and provide the citations.	4, Table 1, and Supplementary Material 4
Critical appraisal within sources of evidence	16	If done, present data on critical appraisal of included sources of evidence (see item 12).	N/A
Results of individual sources of evidence	17	For each included source of evidence, present the relevant data that were charted that relate to the review questions and objectives.	4 to 12 and Supplementary Materials 4 and 5
Synthesis of results	18	Summarize and/or present the charting results as they relate to the review questions and objectives.	Tables 2 to 6
**DISCUSSION**			
Summary of evidence	19	Summarize the main results (including an overview of concepts, themes, and types of evidence available), link to the review questions and objectives, and consider the relevance to key groups.	12 to 13
Limitations	20	Discuss the limitations of the scoping review process.	14
Conclusions	21	Provide a general interpretation of the results with respect to the review questions and objectives, as well as potential implications and/or next steps.	14
**FUNDING**			
Funding	22	Describe sources of funding for the included sources of evidence, as well as sources of funding for the scoping review. Describe the role of the funders of the scoping review.	14

JBI = Joanna Briggs Institute; PRISMA-ScR = Preferred Reporting Items for Systematic reviews and Meta-Analyses extension for Scoping Reviews.

* Where *sources of evidence* (see second footnote) are compiled from, such as bibliographic databases, social media platforms, and Web sites.

† A more inclusive/heterogeneous term used to account for the different types of evidence or data sources (e.g., quantitative and/or qualitative research, expert opinion, and policy documents) that may be eligible in a scoping review as opposed to only studies. This is not to be confused with *information sources* (see first footnote).
‡ The frameworks by Arksey and O'Malley (6) and Levac and colleagues (7) and the JBI guidance (4, 5) refer to the process of data extraction in a scoping review as data charting.
§ The process of systematically examining research evidence to assess its validity, results, and relevance before using it to inform a decision. This term is used for items 12 and 19 instead of "risk of bias" (which is more applicable to systematic reviews of interventions) to include and acknowledge the various sources of evidence that may be used in a scoping review (e.g., quantitative and/or qualitative research, expert opinion, and policy document).

From: Tricco AC, Lillie E, Zarin W, O'Brien KK, Colquhoun H, Levac D, et al. PRISMA Extension for Scoping Reviews (PRISMAScR): Checklist and Explanation. Ann Intern Med. 2018;169:467–473. doi: 10.7326/M18-0850.

Supplementary Material 2

Study Protocol

Rehabilitation delivery models for an ageing population: A scoping review protocol

Keywords: Healthy Aging (Mesh); Aged (Mesh); Rehabilitation (Mesh); Recovery of Function (Mesh); Models, Organizational(Mesh); Health Services for the Aged(Mesh).

Ethical approval: The study did not involve human subjects and thus did not require ethical approval.

Last update: 20 January 2022

Background

The astonishing success of preventive and curative health care services and the global socioeconomic development has led to worldwide population ageing associated with increasing life expectancy and declining fertility rates. However, at the same time, since non-communicable diseases (NCDs) disproportionately affect older people, the world is also facing an epidemiological transition to a higher incidence and prevalence of chronic NCDs[1-3]. A higher morbidity burden leads to a rapid increase in the number of people experiencing disability or declines in functioning, adding pressure to the health and social care systems[4]. These trends urge health policymakers to look for innovative approaches to achieve a not only longer but healthier and more meaningful life.

Healthy life in older age has been called by the World Health Organization in its World Report on Ageing and Health "Healthy Ageing" which is *"the process of developing and maintaining the functional ability that enables well-being in older age."* Functional ability is *"the health-related attributes that enable people to be and to do what they have reason to value, which is made up of the intrinsic capacity of the individual, relevant environmental characteristics, and the interactions between the individual and these characteristics*[2]". The concept of functioning rooted in the WHO International Classification of Functioning, Disability, and Health (ICF)[5] is, in the author's perspective, very similar. Functioning is the *"outcome of complex interactions between the health state of an individual – is determined by health conditions and body functions and structures – and the physical, interpersonal, and social environment*[6]".

Rehabilitation is an essential health service to improve functioning and hence the critical health strategy to achieve healthy ageing[7]. Rehabilitation has shown to be effective in improving functioning; reducing morbidity, including secondary complications, mortality, and health care use, including hospitalization episodes and length of stay; increasing individuals' participation in education, employment, and social life; and extending independent living. Rehabilitation interventions have also been shown to be cost-effective. However, besides its potential, rehabilitation is the health strategy that has received the least attention in the public's mind and among health policymakers. Rehabilitation is still not a priority of the healthy ageing research and policy agenda. There is a lack of knowledge about providing rehabilitation services to the ageing population. We lack a systematic understanding of how these services should be organized, who should benefit from them, what interventions should be provided, and by whom

and how to measure the outcomes of rehabilitation services aiming to foster healthy ageing at the individual and population level.

Aim

To provide an overview of rehabilitation models being used to improve intrinsic capacity or functional ability in the ageing population.

Structured Research Question

P Ageing population (older than 50 years)
I Rehabilitation models, strategies, and programs
C Not applicable
O Healthy ageing, functioning, functional ability, intrinsic capacity

Conceptual framework

Healthy Ageing

"*Is the process of developing and maintaining the functional ability that enables well-being in older age. Functional ability reflects a person's physical and mental capacities, the environments he or she inhabits and the ways in which people interact with their environment*"[8]

Ageing population

"*Is the shift in the distribution of a country's population toward older ages. An increase in the population's mean or median age, a decline in the fraction of the population composed of children, or a rise in the fraction of the population that is elderly*"[9]

Rehabilitation

"*A set of interventions designed to optimize functioning and reduce disability in individuals with health conditions in interaction with their environment*"[10]

Functioning

The concept of functioning is rooted in the WHO International Classification of Functioning, Disability, and Health (ICF)[5] and is the *"outcome of complex interactions between the health state of an individual – is determined by health conditions and body functions and structures – and the physical, interpersonal, and social environment*[6]".

International Classification of Functioning, Disability and Health (ICF)

« It is a classification of health and health-related domains. As the functioning and disability of an individual occurs in a context, ICF also includes a list of environmental factors. ICF is the WHO framework for measuring health and disability at both individual and population levels. It was officially endorsed by all 191 WHO Member States in the Fifty-fourth World Health Assembly on 22 May 2001(resolution WHA 54.21) as the international standard to describe and measure health and disability. ICF is based on the same foundation as ICD and ICHI and share the same set of extension codes that enable documentation at a higher level of detail"[11]

Model of care

"*Is defined as an evolving conception of how services should be delivered. The evolution of the model of care implies changes to services delivery processes in response, including in the design of care, organization of providers, management of services and continuous performance improvement*"[12]

Settings of care

"*Describe the varied types of arrangements for services delivery, organized further into different facilities, institutions and organizations that provide care. Settings include ambulatory, community, home, in-patient and residential services, whereas facilities refer to infrastructure, such as clinics, health centres, district hospitals, dispensaries or other entities, for example, mobile clinics and pharmacies*"[13]

Types of care

"*Refers to the varied aim of services, such as health protection, health promotion, disease prevention, diagnosis, treatment, management, long-term care, rehabilitation, and palliative care, with the specific population intervention and individual services delivered accordingly*"[13]

Care coordination

"*A proactive approach in bringing care professionals and providers together around the needs of service users to ensure that people receive integrated and person-focused care across various settings*"[14]

Integrated health services

"*The management and delivery of health services such that people receive a continuum of health promotion, disease prevention, diagnosis, treatment, disease-management,*

rehabilitation and palliative care services, through the different levels and sites of care within the health system, and according to their needs throughout the life course"[14]

Integrated health services delivery

"*Is defined as an approach to strengthen people- centred health systems through the promotion of the comprehensive delivery of quality services across the life-course, designed according to the multidimensional needs of the population and the individual and delivered by a coordinated multidisciplinary team of providers working across settings and levels of care. It should be effectively managed to ensure optimal outcomes and the appropriate use of resources based on the best available evidence, with feedback loops to continuously improve performance and to tackle upstream causes of ill health and to promote well-being through intersectoral and multisectoral actions*"[13]

People-centred care

"*An approach to care that consciously adopts individuals', carers', families' and communities' perspectives as participants in, and beneficiaries of, trusted health systems that respond to their needs and preferences in humane and holistic ways. People-centred care also requires that people have the education and support they need to make decisions and participate in their own care. It is organized around the health needs and expectations of people rather than diseases*"[14]

Person-centred care

"*Care approaches and practices that see the person as a whole with many levels of needs and goals, with these needs coming from their own personal social determinants of health*"[14]

Person-centredness

"*Is defined as the extent to which the delivery of services adopts a person-facing perspective, including selecting services according to an individual's needs and known risks, designing care to engage patient's in decision-making, organizing providers to realize their delivery, with management and improvement mechanisms in place towards optimal health outcomes*"[13]

People-centred health systems

"*are defined as the design of core health system functions that prioritize the needs of individuals, their families and communities, both as participants and beneficiaries for high-quality comprehensive and coordinated services delivered in an equitable manner and involving people as partners in decision-making*"[13]

Primary care
"*First-contact, accessible, continued, comprehensive and coordinated care to people and communities*"[14]
"*Describes a type of care and setting for health services delivery that supports first-contact, accessible, continued, comprehensive and coordinated care to individuals and communities*"[12]
"*Refers to the concept elaborated in the 1978 Declaration of Alma-Ata, which is based on the principles of equity, participation, intersectoral action, appropriate technology and a central role played by the health system*"[14]

Primary health care
"*Refers to the approach elaborated in the 1978 Declaration of Alma-Ata based on the principles of equity, participation, intersectoral action, appropriate technology and a central role played by the health system for the delivery of services that are made universally accessible to individuals and families in the community through their full participation and at a cost that the community and country can afford to maintain at every stage of their development in the spirit of self-reliance and self-determination*"[12]

Primary care facilities
"*Refer to ambulatory care facilities such as primary care centre, office of generalist health professional, ambulatory health care centre, family planning centre, home health care centre, nursing home, and polyclinic; other settings such as walk-in treatment centre, outpatient department of a district/ general hospital, ambulance, mobile clinic, laboratory, pharmacy, and palliative care establishment; and, rural-specific facilities such as rural physician ambulatory, feldscher assistance point, midwifery post and rural health house*"[12]

Long-term care
"*All activities undertaken by others to ensure that people with, or at risk of, a significant ongoing loss of capacity can maintain a level of functional ability consistent with their basic rights, fundamental freedoms and human dignity*"[15]

Residential long-term care facilities
"*Also known as high dependency care facilities, are establishments primarily engaged in providing inpatient nursing and rehabilitative services to individuals requiring nursing care*"[12]

E-health

"*Information and communication technologies that support the remote management of people and communities with a range of health care needs through supporting self-care and enabling electronic communications between health care professionals and patients*"[14]

M-health

"*The use of mobile technologies to support health information and medical practices, often incorporated into services such as health call centres or emergency number services*"[13]

Telehealth

"*The delivery of distance health services, such as remote clinical diagnosis and monitoring, as well as non-clinical functions, including prevention and promotion of health and curative services*"[13]

Health professionals

"*Health professionals (ISCO-08 22) are professionals who establish and undertake research and develop and apply scientific knowledge in a range of health and related fields including: medicine, complementary medicine, dentistry, optometry, environmental health and occupational health. Specific occupations within the classification of health professionals include: physicians, nursing and midwifery professionals; paramedics; opticians; dentists; speech therapists; dieticians; psychiatrists; and, other health professionals. The tasks undertaken by health professionals involve: conducting research and obtaining scientific knowledge through the study of human and animal disorders; diagnosing illnesses and ways of treating them; the planning, management and evaluation of the care of patients; advising on or dispensing and applying preventive and curative measures; promoting health; and, preparing scientific papers and reports*"[12]

Allied health professionals

"*Refers to a diverse group of health care professionals who provide necessary services to patients in addition to, or in place of, services provided by physicians, nurses and paramedical practitioners. Examples include medical technicians, speech therapists, physical therapists, etc*"[12]

Carers (family carers)

"*Refer to individuals who provide unpaid care for a member or members of their family, friends or community (5). They can be any relative (spouse, children, daughter- and son-in-law), friend or neighbour who provides a broad range of assistance with personal care or basic activities of daily living to people with functional limitations. They may provide regular, occasional or routine, 'hands-on' care or be involved in organizing care delivered by others, sometimes even at-distance. Carers can live with or separately from the person receiving care. Carers are in contrast with providers associated with a formal service system, whether paid or on a volunteer basis (formal caregiver)*"[12]

Self-management or self-care

"*The knowledge, skills and confidence to manage one's own health, to care for a specific condition or to recover from an episode of ill health*"[13]

Levels of care in rehabilitation

"*The degree of specialization of care provided by rehabilitation health professionals. Inclusions: Primary, secondary, and tertiary levels of specialization*"[16]

Integration of care in rehabilitation

"*The management of delivering rehabilitation services in conjunction with other health services so that people receive timely, comprehensive and well-coordinated care, according to their needs and across different levels (vertical integration) and along the continuum of care (horizontal integration). Inclusions: Continuum of care, admission and discharge planning, collaboration of health profes- sionals, shared electronic patients' records*"[16]

Patient-centredness in rehabilitation

"*Rehabilitation tailored on the person's needs and provided in partnership with them, their families and communities. Inclusions: Shared decision-making, individual re- habilitation plan, patient, family or other caregiver's education and empowerment, patient family or other caregiver's integration in the rehabilitation process, involvement of peer counsellors, involvement of patients as prosumers*"[16]

International Classification of Health intervention (ICHI)

"*Is a common tool for reporting and analysing health interventions for clinical and statistical purposes. The classification is built around three axes: Target (the entity on which the Action is carried out), Action (a deed done by an actor to a target) and Means (the processes and*

methods by which the Action is carried out). Extension codes are shared with ICD and ICF in the common foundation and allow users to describe additional detail about the intervention in addition to the relevant ICHI code. A simple, logical syntax links ICHI stem codes and extension codes, interventions performed together, and packages of interventions"[17]

Universal health coverage (UHC) compendium

« The UHC Compendium is a database of health services and intersectoral interventions designed to assist countries in making progress towards Universal Health Coverage (UHC). It provides a strategic way to organize and present information and creates a framework to think about health services and health interventions.[18]

Methodology

The PRISMA extension for scoping reviews (PRISM ScR) checklist will be used to guide the scoping review's methodology.[19]

Eligibility criteria:

Included publications must describe rehabilitation model targeted at the ageing population and must additionally feature the inclusion criteria documented in **Error! Reference source not found.**.

Table 1. Eligibility criteria

	Inclusion criteria	Exclusion criteria
**Population**	The research focus is the ageing population	Does not focus on people older than 50 years or, the paper does not describe the population age in the abstract and does not target a health condition prevalent in people older than 50 years.
**Scope**	Describes a model for providing rehabilitation to the ageing population	Describes needs, functional patterns, disability, risk factors, or protective factors of the ageing population
		Aims to describe or test the effectiveness of a single intervention (e.g., Botulinum toxin for spasticity, exercise for heart failure
		The approach does not include rehabilitation interventions

**Outcome**	Aims to improve the functioning of the ageing population.	Aims to only improve morbidity, mortality, disease control related outcomes (e.g., serum glucose level, blood pressure, medication use, health services use), interventions adherence, interventions' perceived quality and willingness to continue, enjoyment, participation, health service's use, caregivers burden, implementation barriers or health workers perceptions
**Study type**	Original research, including, intervention, observational or descriptive studies	Case report, case series, research protocol, letter to the editor, position paper, book or book chapter, narrative review, systematic review, meta-analysis, conference proceeding, grey literature, guideline or retraction letter
**Language**	Published in English	The full-text study's report is not available or is not available in English.
**Publication year**	From January 2015 to May 2022	

Information sources and search strategy

At least three authors will work together to build the systematic search strategy. We will conduct the search in indexed databases combining the following three main concepts:

1) Rehabilitation and functioning
2) Models of care or health care approaches
3) The ageing population

The search will include natural language and MeSH terms. We will restrict the search to English. We will not search grey literature, but we will revise the reference list of included studies. The final search strategy will be provided as supplementary material during the results dissemination strategy.

Study selection process

Study selection will be completed in two phases:

- Title and abstract screening: Search results will be imported into an online systematic review software called Rayyan[20]. The inclusion criteria will be integrated into the software as a questionnaire that will be developed a priori. To ensure reliability between reviewers, we will

conduct training exercises prior to the formal screening. Teams of two reviewers will use the above eligibility criteria to screen titles and abstracts.

- Full-text screening: Teams of two reviewers will use the same eligibility criteria to screen the full texts of studies in duplicate and independently for eligibility. Similarly, if the decision to exclude or include an article is conflictive, a team meeting will be held with a third team member to make the final decision.

Data extraction:

Data extraction of included papers will be conducted individually and in duplicate. The following information will be extracted using a standardize data extraction form.

→ **General characteristics of the study:** authors, country, publication year, study design, research outcome measures, and paper's conclusion.

→ **Characteristics of the studies' target population:** age-related inclusion criteria, mean age, the number of participants, participants' sex, target population, health condition.

→ **Characteristics of the interventions provided:** data about rehabilitation interventions provided in each study and information about time and intensity.

→ **Characteristics of the rehabilitation services or programs:** level of care, mode of service delivery, organisation of the service provision, including integrated, and information about the providers, including type, team organisation, and role or task shifting or sharing.

Table 2. Variables included in the data extraction form

**Variable**	**Description/instructions**
**PMID**	PMID number if available
**Title**	Copy and paste from the paper
**Publication year**	
**Journal**	
**Authors**	Enter all author's full name
**Abstract**	Copy and paste from the paper
**Country**	Add the country or countries in which the study took place. Use the country's English name, don't use abbreviations, and use capital letters.
**Study design**	Copy and paste the study design reported in the paper

**Target population**	Copy and paste the description from the paper
**Health condition**	Write the health condition's name. If patients with several single health conditions were included, add them separated by a comma without space. For example: diabetes,stroke,hip fracture.<br>If the target population was "patient with multimorbidity," just add multimorbidity. If it was fragility or patients with functional decline, enter not applicable.
**Age related-inclusion criteria**	Copy and paste the description from the paper
**Mean age**	Enter the participant's mean age, up to two decimal points. If the mean age was reported only by groups, sum, and divide by the number of groups. For example, if two groups were included, sum the two mean ages, and divide by two.
**Number of participants**	Enter the total number of participants, including intervention and control group
**Gender (%male)**	Enter the percentage of male participants. Only up to one decimal point
**Description of the intervention**	Copy and paste the entire description from the paper
**Time and intensity**	Enter information like, length of stay or treatment period, intermittent vs continuous treatments, duration of single treatments, number and duration of treatment sessions, and total duration of treatment, service hours.<br>If necessary: add Not reported/No applicable
**Paper's conclusion**	Copy and paste the conclusion from the abstract
**Paper's conclusion category**	Select one of the options: 1) Authors found the intervention effective. 2) Authors found the intervention not effective or not different than the usual care. 3) Can't be assessed
**Outcomes**	If there is more than one outcome, separate them with commas without spaces.<br>Ex: Independence in activities of daily living (Barthel index),Quality of life (SF-36)
**Providers**	Try to match your finding with any of the following:<br>Health care workers, allied health care workers, peers and volunteers, informal caregivers and family, the patient.<br>If there is more than one provider, separate them by commas with no

	spaces.<br>Ex: Health care workers, Community health workers
**Multidisciplinary rehabilitation team**	Enter "Yes" if the at least 3 service providers were involved in the provision of interventions.
**Health workers**	Try to match your finding with any of the following:<br>Nurse, general practitioner, speech and language therapist, physical therapist, occupational therapists<br>prosthetist & orthotists, PRM Physicians, geriatrician, social workers, psychologist, community-based rehabilitation workers. If necessary, enter additional professions for example: neurologist, geriatricians. If there is more than one provider, separate them by commas with no spaces.<br>Ex: Nurse,Social workers,psychologist
**Level of care revised**	Select one of the options: Primary health care, specialized health care or multiple levels of care.
**Mode of service delivery**	Try to match your finding with any of the following:<br>Outpatient, inpatient, home, eldercare institution, telerehabilitation, community<br>If there is more than one mode of service delivery, separate them by commas with no spaces.<br>Ex: outpatient,inpatient,telerehabilitation
**Aim healthy ageing?**	Enter "Yes" if the papers stated the aim of contributing to healthy ageing
**Integrated care?**	Enter "Yes" if the paper matched the following definition of Integrated care:<br>Also known as integrated health, coordinated care, comprehensive care, seamless care, or transmural care, is a worldwide trend in health care reforms and new organizational arrangements focusing on more coordinated and integrated forms of care provision.<br>or the WHO definition: "Integrated care is a concept bringing together inputs, delivery, management and organization of services related to diagnosis, treatment, care, rehabilitation and health promotion. Integration is a means to improve services in relation to access, quality, user satisfaction and efficiency[21].
**Role or task shifting?**	Enter "Yes" if the paper matched the following definition of role or task shifting: a process by which services that are typically delivered by a

	type of health worker are moved to other with less extensive qualifications or training.[22]

Note: Enter not reported or not applicable accordingly.

Data synthesis:

We will use a descriptive approach for the synthesis and presentation of information on the general characteristics of the studies. The synthesis will include quantitative, (e.g., frequency analysis) of study characteristics, rehabilitation interventions, and rehabilitation services provision (i.e., type of providers, level of care), and qualitative analysis to identify rehabilitation models.

References

1. Vos T, Lim SS, Abbafati C, et al. Global burden of 369 diseases and injuries in 204 countries and territories, 1990–2019: a systematic analysis for the Global Burden of Disease Study 2019. The Lancet 2020;396(10258):1204-22.
2. World Health Organization. World report on ageing and health: World Health Organization 2015.
3. The World Health Organization. World Health Statistics 2022: Monitoring health for the Sustainable Development Goals, 2022.
4. Cieza A, Causey K, Kamenov K, et al. Global estimates of the need for rehabilitation based on the Global Burden of Disease study 2019: a systematic analysis for the Global Burden of Disease Study 2019. The Lancet 2020;396(10267):2006-17. doi: 10.1016/s0140-6736(20)32340-0
5. World Health Organization. International Classification of Functioning, Disability and Health (ICF). Geneva, Switzerland, 2001.
6. Stucki G, Bickenbach J. 1.1 Basic Concepts, Definitions and Models. The Journal of the International Society of Physical and Rehabilitation Medicine 2019;2(5) doi: 10.4103/jisprm.jisprm_5_19
7. Stucki G, Bickenbach J, Gutenbrunner C, et al. Rehabilitation: The health strategy of the 21st century. Journal of rehabilitation medicine : official journal of the UEMS European Board of Physical and Rehabilitation Medicine 2018;50(4):309-16. doi: 10.2340/16501977-2200
8. World Health Organization. Decade of Healthy Ageing Baseline Report: World Health Organization 2020.

9. Weil DN. Population aging: National bureau of economic research Cambridge, Mass., USA, 2006.
10. World Healt Organization. Rehabilitation.
11. World Healt Organization. International Classification of Functioning, Disability and Health (ICF) 2001 [Available from: https://www.who.int/standards/classifications/international-classification-of-functioning-disability-and-health accessed 05.09.2022.
12. World Health Organization, Regional office for Europe. GLOSSARY OF TERMS: WHO European Primary Health Care Impact, Performance and Capacity Tool (PHC-IMPACT). https://www.euro.who.int/__data/assets/pdf_file/0006/421944/Glossary-web-171219.pdf.
13. World Health Organization, Regional office for Europe. Glosary of terms: The European Framework for Action on Integrated Health Services Delivery, 2016.
14. World Health Organization. WHO global strategy on integrated people-centred health services 2016-2026: Placing people and communities at the centre of health services, 2015.
15. Pot AM, Briggs AM, Beard JR, et al. Healthy Ageing and the need for a Long- term-care system: Global consultation on integrated care for older people (ICOPE)– the path to universal health coverage, 2017.
16. Gutenbrunner C, Nugraha B, Gimigliano F, et al. International Classification of Service Organization in Rehabilitation: An updated set of categories (ICSO-R 2.0). Journal of rehabilitation medicine : official journal of the UEMS European Board of Physical and Rehabilitation Medicine 2020;52(1):jrm00004. doi: 10.2340/16501977-2627 [published Online First: 20200102]
17. World Healt Organization. International Classification of Health Interventions (ICHI).
18. World Healt Organization. Universal Health Coverage Compendium
19. Tricco AC, Lillie E, Zarin W, et al. PRISMA extension for scoping reviews (PRISMA-ScR): checklist and explanation. Annals of internal medicine 2018;169(7):467-73.
20. Ouzzani M, Hammady H, Fedorowicz Z, et al. Rayyan—a web and mobile app for systematic reviews. Systematic reviews 2016;5(1):1-10.
21. Gröne O, Garcia-Barbero M. Integrated care: a position paper of the WHO European Office for Integrated Health Care Services. International journal of integrated care 2001;1
22. Organization WH. The world health report 2006: working together for health: World Health Organization 2006.

Supplementary Material 3

Search concepts, terms, and search strategies for each bibliographic database queried.

Table S1. Summary of search concepts and terms

Concepts	Natural language	Medical subject headings
**Rehabilitation**	functioning, convalescence, Telerehabilitation, restorative, rehabi*, reablement, re–ablement, enablement, "speech therapy", "self management" , “self-management", "self care" , "self-care", "rehabilitation medicine", recover*, "recovery of function", "physical therapy", "physical function", "physical exercise", "patient* recovery", "occupational therapy", "occupational functioning", "language therapy", "intrinsic capacit*", "instrumental ADLs", "instrumental activities of daily living", "healthy aging", "functional status", "Functional recovery", "functional health", "functional gain", "functional autonomy", "functional abilit*", "cognitive function*", "activities of daily living", "active ageing"	Convalescence, Rehabilitation Research, Rehabilitation Centers, Telerehabilitation, Speech Therapy, Self-Management, Self Efficacy, Self Care, Rehabilitation, Recovery of Function, Physical Therapy Modalities, Physical and Rehabilitation Medicine, Occupational Therapy, Language Therapy, International Classification of Functioning, Disability and Health, Independent Living, Healthy Aging, Activities of Daily Living.
**Ageing population**	frail, fragility, geria*, retir*, seni*, "old age", "physical decline*", "cognitive decline*", "community-dwelling older adults", "function* impairment*", "Functioning decline", ageing, aging, elder*, "older patients", "older adult*", "older person*", "older people"	Aged, 80 and over, Aged, Middle Aged, Frail Elderly, Geriatrics, Age Factors, Sarcopenia

**Services**	"health planning", "health program", "health service*", "patient care planning", "Community-based", "Community health service", telemonitoring, "transitional care", telehealth, Telerehabilitation, "Long-Term Care", "care model", "team approach", Telecare	Treatment Outcome, Telerehabilitation, Telemedicine, Senior Centers, Program Evaluation, Primary Health Care, Patient Care Team, Patient Care Management, Models, Organizational, Long-Term Care, Home Care Services, Home Care Services, Hospital-Based, Health Services for the Aged, Geriatric Assessment, Delivery of Health Care, Integrated, Comprehensive Health Care, Community Health Nursing, Transitional Care, Quality of Health Care, Program Development, Patient-Centered Care, Outpatient Clinics, Hospital, National Health Programs, Health Services, Health Services Needs and Demand, Health Planning, Community Health Services

Table S2. Academic Search Medline search strategy

#	Query	Results
**S124**	S43 AND S69 AND S122	17,285
**S123**	S43 AND S69 AND S122	47,440
**S122**	(S70 OR S71 OR S72 OR S73 OR S74 OR S75 OR S76 OR S77 OR S78 OR S79 OR S80 OR S81 OR S82 OR S83 OR S84 OR S85 OR S86 OR S87 OR S88 OR S89 OR S90 OR S91 OR S92 OR S93 OR S94 OR S95 OR S96 OR S97 OR S98 OR S99 OR S100 OR S101 OR S102 OR S103 OR S104 OR	1,459,100

	S105 OR S106 OR S107 OR S108 OR S109 OR S110 OR S111 OR S112 OR S113 OR S114 OR S115 OR S116 OR S117 OR S118 OR S119 OR S120 OR S121)	
**S121**	TI functioning OR AB functioning	203,555
**S120**	TI convalescence OR AB convalescence	5,210
**S119**	TI Telerehabilitation OR AB Telerehabilitation	1,136
**S118**	TI restorative OR AB restorative	23,889
**S117**	TI rehabi* OR AB rehabi*	187,961
**S116**	TI reablement OR AB reablement	122
**S115**	TI re–ablement OR AB re–ablement	19
**S114**	TI enablement OR AB enablement	649
**S113**	TI "speech therapy" OR AB "speech therapy"	2,659
**S112**	TI "self management" OR AB "self management" OR TI "self-management" OR AB "self-management"	22,395
**S111**	TI "self care" OR AB "self care" OR TI "self-care" OR AB "self-care"	21,067
**S110**	TI "rehabilitation medicine" OR AB "rehabilitation medicine"	1,936
**S109**	TI recover* OR AB recover*	742,299
**S108**	TI "recovery of function" OR AB "recovery of function"	2,500
**S107**	TI "physical therapy" OR AB "physical therapy"	20,119
**S106**	TI "physical function" OR AB "physical function"	16,488
**S105**	TI "physical exercise" OR AB "physical exercise"	17,758
**S104**	TI "patient* recovery" OR AB "patient* recovery"	3,652
**S103**	TI "occupational therapy" OR AB "occupational therapy"	10,884
**S102**	TI "occupational functioning" OR AB "occupational functioning"	1,159
**S101**	TI "language therapy" OR AB "language therapy"	1,396
**S100**	TI "intrinsic capacit*" OR AB "intrinsic capacit*"	865
**S99**	TI "instrumental ADLs" OR AB "instrumental ADLs"	203
**S98**	TI "instrumental activities of daily living" OR AB "instrumental activities of daily living"	4,595
**S97**	TI "healthy aging" OR AB "healthy aging"	5,560

**S96**	TI "functional status" OR AB "functional status"	27,844
**S95**	TI "Functional recovery" OR AB "Functional recovery"	23,888
**S94**	TI "functional health" OR AB "functional health"	2,277
**S93**	TI "functional gain" OR AB "functional gain"	621
**S92**	TI "functional autonomy" OR AB "functional autonomy"	488
**S91**	TI "functional abilit*" OR AB "functional abilit*"	7,554
**S90**	TI "cognitive function*" OR AB "cognitive function*"	72,253
**S89**	TI "activities of daily living" OR AB "activities of daily living"	30,175
**S88**	TI "active ageing" OR AB "active ageing"	290
**S87**	(MH "Convalescence")	3,899
**S86**	(MH "Rehabilitation Research")	232
**S85**	(MH "Rehabilitation Centers")	8,532
**S84**	(MH "Telerehabilitation")	754
**S83**	(MH "Speech Therapy")	6,701
**S82**	(MH "Self-Management")	4,424
**S81**	(MH "Self Efficacy")	23,098
**S80**	(MH "Self Care")	35,221
**S79**	(MH "Rehabilitation")	18,647
**S78**	(MH "Recovery of Function")	58,180
**S77**	(MH "Physical Therapy Modalities")	39,346
**S76**	(MH "Physical and Rehabilitation Medicine")	3,486
**S75**	(MH "Occupational Therapy")	14,210
**S74**	(MH "Language Therapy")	2,040
**S73**	(MH "International Classification of Functioning, Disability and Health")	775
**S72**	(MH "Independent Living")	9,537
**S71**	(MH "Healthy Aging")	1,721
**S70**	(MH "Activities of Daily Living")	70,033

**S69**	S44 OR S45 OR S46 OR S47 OR S48 OR S49 OR S50 OR S51 OR S52 OR S53 OR S54 OR S55 OR S56 OR S57 OR S58 OR S59 OR S60 OR S61 OR S62 OR S63 OR S64 OR S65 OR S66 OR S67 OR S68	5,821,140
**S68**	TI frail OR AB frail	14,773
**S67**	TI fragility OR AB fragility	18,027
**S66**	TI geria* OR AB geria*	55,508
**S65**	TI retir* OR AB retir*	24,285
**S64**	TI seni* OR AB seni*	65,198
**S63**	TI "old age" OR AB "old age"	27,502
**S62**	TI "physical decline*" OR AB "physical decline*"	441
**S61**	TI "cognitive decline*" OR AB "cognitive decline*"	27,657
**S60**	TI "community-dwelling older adults" OR AB "community-dwelling older adults"	7,180
**S59**	TI "function* impairment*" OR AB "function* impairment*"	25,251
**S58**	TI "Functioning decline" OR AB "Functioning decline"	44
**S57**	TI ageing OR AB ageing	47,107
**S56**	TI aging OR AB aging	203,996
**S55**	TI elder* OR AB elder*	282,288
**S54**	TI "older patients" OR AB "older patients"	44,253
**S53**	TI "older adult*" OR AB "older adult*"	99,963
**S52**	TI "older person*" OR AB "older person*"	12,707
**S51**	TI "older people" OR AB "older people"	33,134
**S50**	(MH "Aged, 80 and over")	1,003,407
**S49**	(MH "Aged")	3,339,004
**S48**	(MH "Middle Aged")	4,669,969
**S47**	(MH "Frail Elderly")	13,772
**S46**	(MM "Geriatrics")	27,118
**S45**	(MM "Age Factors")	6,297
**S44**	(MH "Sarcopenia")	7,326

**S43**	S1 OR S2 OR S3 OR S4 OR S5 OR S6 OR S7 OR S8 OR S9 OR S10 OR S11 OR S12 OR S13 OR S14 OR S15 OR S16 OR S17 OR S18 OR S19 OR S20 OR S21 OR S22 OR S23 OR S24 OR S25 OR S26 OR S27 OR S28 OR S29 OR S30 OR S31 OR S32 OR S33 OR S34 OR S35 OR S36 OR S37 OR S38 OR S39 OR S40 OR S41 OR S42	833,703
**S42**	TI "health planning" OR AB "health planning"	3,600
**S41**	TI "health program" OR AB "health program"	5,859
**S40**	TI "health service*" OR AB "health service*"	120,965
**S39**	TI "patient care planning" OR AB "patient care planning"	114
**S38**	TI "Community-based" OR AB "Community-based"	68,095
**S37**	TI "Community health service" OR AB "Community health service"	503
**S36**	TI telemonitoring OR AB telemonitoring	1,816
**S35**	TI "transitional care" OR AB "transitional care"	1,668
**S34**	TI telehealth OR AB telehealth	7,883
**S33**	TI Telerehabilitation OR AB Telerehabilitation	1,136
**S32**	TI "Long-Term Care" OR AB "Long-Term Care"	22,967
**S31**	TI program	165,422
**S30**	TI "care model" OR AB "care model"	6,323
**S29**	TI "team approach" OR AB "team approach"	6,898
**S28**	TI Telecare OR AB Telecare	710
**S27**	(MM "Treatment Outcome")	8,065
**S26**	(MH "Telerehabilitation")	754
**S25**	(MH "Telemedicine")	33,078
**S24**	(MH "Senior Centers")	104
**S23**	(MM "Program Evaluation")	11,451
**S22**	(MM "Primary Health Care")	54,906
**S21**	(MM "Patient Care Team")	28,993
**S20**	(MM "Patient Care Management")	3,018
**S19**	(MM "Models, Organizational")	6,482
**S18**	(MH "Long-Term Care")	27,588

**S17**	(MH "Home Care Services")	35,507
**S16**	(MM "Home Care Services, Hospital-Based")	1,579
**S15**	(MH "Health Services for the Aged")	18,137
**S14**	(MH "Geriatric Assessment")	31,005
**S13**	(MH "Delivery of Health Care, Integrated")	13,902
**S12**	(MH "Comprehensive Health Care")	6,747
**S11**	(MH "Community Health Nursing")	19,737
**S10**	(MH "Transitional Care")	1,111
**S9**	(MH "Quality of Health Care")	76,490
**S8**	(MH "Program Development")	30,183
**S7**	(MH "Patient-Centered Care")	21,980
**S6**	(MH "Outpatient Clinics, Hospital")	15,835
**S5**	(MH "National Health Programs")	33,146
**S4**	(MH "Health Services")	26,636
**S3**	(MH "Health Services Needs and Demand")	54,889
**S2**	(MH "Health Planning")	21,898
**S1**	(MH "Community Health Services")	32,760
**Limiters: Date of Publication: 20150101-20220531; English Language; Human**		

Table S3. Academic Search Medline search strategy

#	Query	Results
**#11**	#7 AND #8 AND #9	4,867
**#10**	#7 AND #8 AND #9	12,197
**#9**	#3 OR #6	1,626,662
**#8**	#2 OR #5	898,194
**#7**	#1 OR #4	917,320
**#6**	functioning:ti,kw OR convalescence:ti,kw OR telerehabilitation:ab,ti,kw OR restorative:ti,kw OR rehabi*:ab,ti,kw OR reablement:ab,ti,kw OR re–	1,469,268

	ablement:ab,ti,kw OR enablement:ab,ti,kw OR 'speech therapy':ab,ti,kw OR 'self management':ti,kw OR 'self-management':ti,kw OR 'self care':ti,kw OR 'self-care':ti,kw OR 'rehabilitation medicine':ab,ti,kw OR recover*:ab,ti,kw OR 'recovery of function':ab,ti,kw OR 'physical therapy':ab,ti,kw OR 'physical function':ti,kw OR 'physical exercise':ti,kw OR 'patient*recovery':ti,kw OR 'occupational therapy':ab,ti,kw OR 'occupational functioning':ab,ti,kw OR 'language therapy':ab,ti,kw OR 'intrinsic capacit*':ab,ti,kw OR 'instrumental adls':ab,ti,kw OR 'instrumental activities of daily living':ab,ti,kw OR 'healthy aging':ab,ti,kw OR 'functional status':ti,kw OR 'functional recovery':ab,ti,kw OR 'functional health':ab,ti,kw OR 'functional gain':ab,ti,kw OR 'functional autonomy':ab,ti,kw OR 'functional abilit*':ab,ti,kw OR 'cognitive function*':ti,kw OR 'activities of dailyliving':ab,ti,kw OR 'active ageing':ab,ti,kw	
**#5**	frail:ab,ti,kw OR fragility:ab,ti,kw OR geria*:ab,ti,kw OR retir*:ti,kw OR seni*:ab,ti,kw OR 'old age':ti,kw OR 'physical decline*':ab,ti,kw OR 'cognitive decline*':ab,ti,kw OR 'community-dwelling older adults':ab,ti,kw OR 'function* impairment*':ti,kw OR 'functioning decline':ti,kw OR ageing:ti,kw OR aging:ti,kw OR elder*:ab,ti,kw OR 'older patients':ti,kw OR 'older adult*':ti,kw OR 'older person*':ti,kw OR 'older people':ti,kw	860,667
**#4**	'health program':ab,ti,kw OR 'health service*':ab,ti,kw OR 'patient care planning':ab,ti,kw OR 'community-based':ab,ti,kw OR 'community health service':ab,ti,kw OR telemonitoring:ab,ti,kw OR 'transional care':ab,ti,kw OR telehealth:ab,ti,kw OR telerehabilitation:ab,ti,kw OR 'long-term care':ab,ti,kw OR program:ti OR 'care model':ab,ti,kw OR 'team approach':ab,ti,kw OR telecare:ab,ti,kw	479,293
**#3**	'convalescence'/mj OR 'rehabilitation research'/exp OR 'rehabilitation center'/exp OR 'telerehabilitation'/exp OR 'speech therapy'/exp OR 'self care'/mj OR 'rehabilitation'/exp/mj OR 'physiotherapy'/exp OR 'rehabilitation medicine'/exp OR 'occupational therapy'/exp OR 'language therapy'/exp OR 'international classification of functioning, disability and health'/exp OR 'independent living'/exp OR 'healthy aging'/exp OR 'daily life activity'/mj	329,501
**#2**	'very elderly'/exp/mj OR 'aged'/exp/mj OR 'middle aged'/exp/mj	108,048

	OR 'geriatrics'/exp/mj OR 'sarcopenia'/exp/mj	
**#1**	treatment outcome'/mj OR 'telerehabilitation'/mj OR 'telemedicine'/mj OR 'senior center'/mj OR 'program evaluation'/mj OR 'primary health care'/mj OR 'patient care'/mj OR 'long term care'/mj OR 'home care'/mj OR 'elderly care'/exp/mj OR 'geriatric assessment'/mj OR 'integrated health care system'/mj OR 'health care'/mj OR 'community health nursing'/mj OR 'transitional care'/mj OR 'health care quality'/mj OR 'program development'/exp/mj OR 'outpatient department'/mj OR 'health service'/mj OR 'health care planning'/mj OR 'community care'/mj	507,343
**Limiters: Date of Publication: 20150101-20220531; English Language; Human**		

Supplementary Material 4

Supplementary Table 1. Included studies' most important characteristics

First author, year	Country	Study design	Authors' conclusion	Target population	Health condition	Health condition area	Inclusion criterion in years	N° participants	Participants' mean age	Participants' sex predominance
**Diaz Baquero, A. A., 2022**	Spain	Intervention study	Effective	Single health condition	Cognitive impairment including dementia or aphasia	Neurological	_	62	74.73	Female
**Liang, C. C., 2022**	Taiwan	Observational Study	Effective	Fragility or functional decline	_	_	> 65	56	76.5	Female
**Porta, F. L., 2022**	Italy	Intervention study	Not effective	Fragility or functional decline	_	_	> 65	403	76.2	Female
**Yee, D. K. H., 2022**	China (Hong Kong)	Observational Study	Effective	Single health condition	Hip fracture or post hip arthroplasty	Musculoskeletal	> 65	401	84.2	Female
**Poulsen, L. K., 2022**	Denmark	Intervention study	No assessed	Single health condition	Hip fracture or post hip arthroplasty	Musculoskeletal	_	48	78	Female
**Stathi, A., 2022**	UK	Intervention study	Effective	Fragility or functional decline	_	_	> 65	777	77.6	Female
**Tekin, F., 2022**	Turkey	Intervention study	Effective	Fragility or functional decline	_	_	> 65	255	69	Balanced
**Hoedemakers, M., 2022**	Netherlands	Intervention study	No assessed	Fragility or functional decline	_	_	_	384	83.4	Female
**Ropke, A., 2022**	Denmark	Intervention study	No assessed	Single health condition	Hip fracture or post hip arthroplasty	Musculoskeletal	> 65	18	79.4	Female
**Bennell, K. L., 2022**	Australia	Intervention study	Effective	Single health condition	Osteoarthritis	Musculoskeletal	Other	414	65	Balanced
**Chong, E., 2022**	Singapore	Intervention study	Effective	Fragility or functional decline	_	_	> 65	140	79.7	Female
**Kam Yuet Wong, F., 2022**	China	Intervention study	Effective	Single health condition	Stroke	Neurological	>18	116	66.6	Male
**Love, 2022**	Australia	Observational Study	No assessed	Multimorbidity	_	_	> 65	92	81.1	Balanced
**Kalav, S., 2022**	Turkey	Intervention study	No assessed	Single health condition	Stroke	Neurological	>18	68	57.4	Male
**Ullrich, P., 2022**	Germany	Intervention study	Effective	Multimorbidity	_	_	> 65	118	82.3	Female
**Mora-Traverso, M., 2022**	Spain	Intervention study	Effective	Single health condition	Hip fracture or post hip arthroplasty	Musculoskeletal	> 65	64	78.22	Female

**Gustavson, A. M., 2022**	US	Intervention study	No assessed	Fragility or functional decline	_	_	_	28	78.5	Female
**Tao, G., 2022**	Canada	Intervention study	No assessed	Single health condition	Lower limb amputation	Musculoskeletal	> 50	71	65	Male
**Yang, L., 2022**	China	Intervention study	Effective	Single health condition	Cognitive impairment including dementia or aphasia	Neurological	> 65	126	85.36	Female
**Furuta, K., 2022**	Japan	Intervention study	Effective	Fragility or functional decline	_	_	< 85	25	76	Female
**Li, C. T., 2022**	China (Hong Kong)	Intervention study	No assessed	Single health condition	Hip fracture or post hip arthroplasty	Musculoskeletal	> 60	31	79	Female
**Tan, J. Y., 2022**	US	Descriptive study	No assessed	Single health condition	HIV	Communicable diseases	> 50	13	_	Male
**U. N. Yadav, 2021**	Nepal	Descriptive study	No assessed	Multimorbidity	_	_	_	_	_	_
**S. Tanaka, 2021**	Japan	Intervention study	Effective	Single health condition	COPD	Neurological	_	31	86.15	Balanced
**A. Sondell, 2021**	Sweden	Intervention study	Effective	Single health condition	COPD	Neurological	> 60	16	78.5	Female
**W. Rietkerk, 2021**	Netherlands	Intervention study	Effective	Fragility or functional decline	_	_	> 75	233	81.5	Female
**M. E. Ribbink, 2021**	Netherlands, Spain	Observational Study	No assessed	Multimorbidity	_	_	_	1038	83.85	Balanced
**R. Quigley, 2021**	Australia	Descriptive study	Effective	Fragility or functional decline	_	_	Other	26	87	Balanced
**C. L. Peiris, 2021**	Australia	Observational Study	Effective	Single health condition	Osteoarthritis	Musculoskeletal	> 65	230	70.5	Female
**M. Morri, 2021**	Italy	Observational Study	Effective	Single health condition	Hip fracture or post hip arthroplasty	Musculoskeletal	> 65	923	84.1	Female
**H. Kim, 2021**	South Korea	Intervention study	Effective	Fragility or functional decline	_	_	_	913	82.9	Female
**A. Flynn, 2021**	Australia	Intervention study	No assessed	Single health condition	Parkinson's disease	Neurological	_	40	72	Male
**R. De Luca, 2021**	Italy	Intervention study	Effective	Fragility or functional decline	_	_	> 65	60	77.4	Female
**S. Coskun, 2021**	Turkey	Intervention study	Effective	Single health condition	Coronary heart disease	Cardiovascular	> 60	66	68.13	Male
**Y. Baba, 2021**	Japan	Intervention study	Effective	Fragility or functional decline	_	_	> 65	78	83.65	Female

**Coletta, A. M., 2021**	United States of America	Observational Study	Effective	Single health condition	Breast cancer, prostate cancer, lymphoma	Cancer	_	239	62.1	Female
**Lamberti, N., 2021**	Italy	Observational Study	Effective	Single health condition	Peripheral artery disease	Cardiovascular	>18	83	72	Male
**Maniscalco, M., 2021**	Italy	Intervention study	Effective	Multimorbidity	Cardiovascular disease not specified, covid-19	Cardiovascular	_	95	63.4	Male
**Otero, P., 2021**	Spain	Intervention study	Effective	Fragility or functional decline	_	_	Other	25	54.9	Female
**Tseng, M. Y., 2021**	Taiwan	Intervention study	Not effective	Multimorbidity	Hip fracture or post hip arthroplasty, cognitive impairment including dementia or aphasia	Musculoskeletal, neurological	> 60	152	81.8	Female
**Kim, S., 2021**	Korea	Intervention study	Effective	Fragility or functional decline	_	_	> 65	224	75	Female
**Sun, F. C., 2021**	Taiwan	Intervention study	Effective	Fragility or functional decline	_	_	> 65	122	73.9	Female
**Farinha, C., 2021**	Portugal	Intervention study	Effective	Fragility or functional decline	_	_	> 65	102	71.4	Female
**Hayden, M. C., 2021**	Germany	Observational Study	Effective	Single health condition	Covid-19	Respiratory, communicable diseases	>18	108	55.6	Balanced
**Barker, K. L., 2021**	England	Intervention study	Not effective	Single health condition	Osteoarthritis	Musculoskeletal	Other	621	70.4	Balanced
**Meisingset, I., 2021**	Norway	Observational Study	Effective	Fragility or functional decline	_	_	> 65	603	84	Female
**Al Chikhanie, Y., 2021**	France	Observational Study	No assessed	Single health condition	COPD	Respiratory	_	190	66	Balanced
**Suikkanen, S., 2021**	Finland	Intervention study	Effective	Fragility or functional decline	_	_	> 65	300	82.2	Female
**Brouwers, R. W. M., 2021**	Netherlands	Intervention study	Effective	Single health condition	Coronary heart disease	Cardiovascular	_	300	60.7	Male
**Patel, N., 2021**	United States of America	Observational Study	Effective	Single health condition	Covid-19	Respiratory, communicable diseases	_	106	64	Male
**Yi, D., 2021**	Korea	Intervention study	Effective	Single health condition	Covid-19	Respiratory, communicable diseases	> 65	70	76.7	Female

**Chi, Y. C., 2021**	Taiwan	Intervention study	Effective	Fragility or functional decline	_	_	> 65	_	_	Female
**Gutierrez-Espinoza, H., 2021**	Chile	Observational Study	Effective	Single health condition	Complex regional pain syndrome type I, distal radius fracture	Musculoskeletal	> 60	72	52.8	Female
**Su, S. F., 2021**	Taiwan	Intervention study	Effective	Single health condition	Hip fracture or post hip arthroplasty	Musculoskeletal	> 60	104	77.7	Female
**Hosteng, K. R., 2021**	United States of America	Intervention study	Effective	Fragility or functional decline	_	_	> 65	68	81.2	Female
**Chiu, E. C., 2021**	Taiwan	Intervention study	Effective	Single health condition	Stroke	Neurological	Other	26	68	Male
**Martinez-Velilla, N., 2021**	Chile	Intervention study	Effective	Fragility or functional decline	_	_	_	297	87.3	Balanced
**Gutierrez-Lopez, L., 2021**	Mexico	Intervention study	Effective	Single health condition	Sarcopenia	Metabolic, musculoskeletal	< 70	60	68	Female
**Sen, E. I., 2021**	Turkey	Intervention study	Effective	Single health condition	Sarcopenia	Musculoskeletal	< 80	90	72.8	Female
**Sok, S., 2021**	Korea	Intervention study	Effective	Fragility or functional decline	_	_	> 65	65	73.7	Female
**Jungreitmayr, S., 2021**	Austria, Italy	Intervention study	Effective	Fragility or functional decline	_	_	< 85	72	75.5	Female
**Liang, C. K., 2021**	Taiwan	Intervention study	Effective	Fragility or functional decline	_	_	> 65	733	74	Female
**Oh, S. L., 2021**	South Korea	Intervention study	Effective	Single health condition	Osteoarthritis	Musculoskeletal	_	60	71.7	Female
**Pakrad, F., 2021**	Iran	Intervention study	Effective	Single health condition	Coronary heart disease	Cardiovascular	_	88	62.7	Female
**Siercke, M., 2021**	Denmark	Intervention study	Effective	Single health condition	Peripheral artery disease	Cardiovascular	>18	118	70.3	Balanced
**Tou, N. X., 2021**	Singapore	Intervention study	Effective	Fragility or functional decline	_	_	Other	57	71.7	Female
**Monteblanco Cavalcante, M., 2021**	Brazil	Intervention study	Effective	Fragility or functional decline	_	_	_	9	71.9	Balanced
**Borges-Machado, F., 2021**	Portugal	Intervention study	Effective	Single health condition	Cognitive impairment including dementia or aphasia	Neurological	> 60	43	78.2	Female
**Murphy, S. L., 2021**	United States of America	Intervention study	Not effective	Single health condition	Diffuse cutaneous systemic sclerosis	Autoimmune disease	>18	32	52	Female

**Bruce, J., 2021**	England	Intervention study	Effective	Single health condition	Breast cancer	Cancer	>18	392	58.1	Female
**Rosado, H., 2021**	Portugal	Intervention study	Effective	Fragility or functional decline	_	_	> 65	51	75.4	Male
**Tian, R., 2021**	China	Intervention study	Effective	Single health condition	Cognitive impairment including dementia or aphasia	Neurological	< 80	93	66.9	Female
**Noh, E. Y., 2021**	Korea	Observational Study	Effective	Fragility or functional decline	_	_	> 65	877	_	Female
**Aguilera-Godoy, A., 2021**	Chile	Observational Study	No assessed	Single health condition	Distal radius fracture	Musculoskeletal	> 60	36	68.5	Female
**Mentz, R. J., 2021**	United States of America	Observational Study	Effective	Single health condition	Heart failure	Cardiovascular	> 60	349	72.7	Balanced
**Pepera, G., 2021**	Greece	Intervention study	Effective	Fragility or functional decline	_	_	> 65	40	79.4	Female
**Rince, G., 2021**	France	Intervention study	Effective	Fragility or functional decline	_	_	> 70	483	83.1	Female
**Arena, S. K., 2021**	United States of America	Intervention study	Effective	Fragility or functional decline	_	_	> 65	110	76.9	Female
**Scherrenberg, M., 2021**	The Netherlands, Denmark, France, Spain, Switzerland	Intervention study	Effective	Single health condition	Cardiovascular disease not specified	Cardiovascular	_	162	72.8	Male
**Mao, H. F., 2021**	Taiwan	Intervention study	Effective	Fragility or functional decline	_	_	> 65	130	78.2	Female
**Rafiq, M. T., 2021**	Malaysia	Intervention study	Effective	Multimorbidity	_	_	Other	114	52.9	Balanced
**Bagkur, M., 2021**	Cyprus	Intervention study	Effective	Fragility or functional decline	_	_	< 90	23	72.4	Female
**Backer, H. C., 2021**	Germany	Intervention study	Effective	Single health condition	Osteoarthritis	Musculoskeletal	< 85	60	64.3	Balanced
**Katri Maria, T., 2021**	Finland	Intervention study	No assessed	Fragility or functional decline	_	_	> 60	117	79.8	Female
**Ozen, S., 2021**	Turkey	Intervention study	Effective	Single health condition	Stroke	Cardiovascular	< 85	38	65.9	Male
**Soukkio, P. K., 2021**	Finland	Intervention study	Not effective	Single health condition	Hip fracture or post hip arthroplasty	Musculoskeletal	> 60	121	81.5	Female
**Woo, J., 2021**	China (Hong Kong)	Descriptive study	No assessed	Fragility or functional decline	_	_	_	102	84	Female

**Lim, J. H., 2021**	Republic of Korea	Intervention study	Effective	Single health condition	Stroke	Neurological	_	17	70.11	Balanced
**Song, C. Y., 2021**	Taiwan	Intervention study	Effective	Fragility or functional decline	_	_	> 65	28	76	Female
**Z. Wu, 2020**	China	Intervention study	Effective	Single health condition	Coronary heart disease	Neurological	< 80	64	57.6	Male
**J. Uddin, 2020**	Bangladesh	Intervention study		Single health condition	Coronary heart disease	Cardiovascular	Other	142	54	Male
**K. A. Spencer, 2020**	United States of America	Intervention study	No assessed	Single health condition	Parkinson's disease	Neurological	> 50	3	62	Male
**Y. Song, 2020**	China	Intervention study	Effective	Single health condition	Coronary heart disease	Cardiovascular	Other	96	54.45	Male
**H. N. Smith, 2020**	Australia	Descriptive study	Effective	Fragility or functional decline	_	_	Other	110	80.4	Balanced
**J. Sedlock, 2020**	United States of America	Descriptive study	No assessed	Single health condition	Fracture not specified	Musculoskeletal	> 65	_	_	_
**J. Room, 2020**	United Kingdom	Descriptive study	No assessed	Single health condition	Osteoarthritis	Musculoskeletal	Other	635	_	_
**V. Provencher, 2020**	Australia	Intervention study	No assessed	Fragility or functional decline	_	_	> 70	400	80.5	Female
**M. Parsons, 2020**	New Zealand	Intervention study	Effective	Single health condition	Injuries	Musculoskeletal	> 65	403	80.8	Female
**A. Nakayama, 2020**	Japan	Observational Study	Effective	Single health condition	Heart failure	Cardiovascular	_	236	66	Balanced
**I. Nabutovsky, 2020**	Israel	Intervention study	Effective	Single health condition	Coronary heart disease	Cardiovascular	_	22	52.7	Male
**J. S. Myers, 2020**	United States of America	Intervention study	Effective	Single health condition	Breast cancer	Cancer	>18	61	55.6	Female
**J. Miller, 2020**	Canada	Intervention study	Effective	Multimorbidity	_	_	_	102	52.8	Female
**M. Markle-Reid, 2020**	Canada	Intervention study	Not effective	Single health condition	Stroke	Neurological	Other	30	71.6	Balanced
**J. Mann, 2020**	Australia	Descriptive study	No assessed	Fragility or functional decline	_	_	Other	_	_	_
**W.-Y. Li, 2020**	Taiwan	Intervention study	Effective	Single health condition	Chronic kidney disease	Urological	Other	49	51.2	Male
**M. Kraepelien, 2020**	Sweden	Intervention study	Effective	Single health condition	Parkinson's disease	Neurological	_	77	66	Female

**E. H. Koolen, 2020**	Netherlands	Intervention study	No assessed	Single health condition	COPD	Respiratory	_	402	63	Balanced
**M. V. Knudsen, 2020**	Denmark	Intervention study	Not effective	Single health condition	Coronary heart disease	Cardiovascular	_	77	58	Male
**O. Kamoen, 2020**	Belgium	Observational Study	Effective	Single health condition	Stroke	Neurological	>18	214	68.15	Male
**H. Hansen, 2020**	Denmark	Intervention study	Not effective	Single health condition	COPD	Respiratory	_	134	68.3	Balanced
**A. M. Gustavson, 2020**	United States of America	Intervention study	No assessed	Fragility or functional decline	_	_	_	103	77.65	Male
**A. W. Deng, 2020**	China	Intervention study	Effective	Single health condition	Stroke	Neurological	_	98	61.4	Male
**J. de Batlle, 2020**	Spain	Intervention study	Effective	Fragility or functional decline	_	_	Other	135	78	Balanced
**S. S. Conroy, 2020**	United States of America	Intervention study	Effective	Single health condition	Stroke	Neurological	>18	29	60.95	Balanced
**D. Hevey, 2020**	United Kingdom	Intervention study	Effective	Multimorbidity	_	_	_	273	_	Female
**D. S. Lipardo, 2020**	Philippines	Intervention study	Effective	Fragility or functional decline	_	_	> 60	92	69	Female
**K. Laver, 2020**	Australia	Intervention study	Effective	Single health condition	Cognitive impairment including dementia or aphasia	Neurological	_	63	79.9	Balanced
**A. K. C. Wong, 2019**	China (Hong Kong)	Intervention study	Effective	Fragility or functional decline	_	_	> 60	457	78	Female
**K. von Storch, 2019**	Germany	Intervention study	Effective	Single health condition	Diabetes	Metabolic	Other	115	58.9	Balanced
**M. Ranner, 2019**	Sweden	Descriptive study	Effective	Single health condition	Stroke	Neurological	_	10	68	Male
**B. B. Nilsson, 2019**	Norway	Observational Study	Effective	Single health condition	Cardiovascular disease not specified	Cardiovascular	_	273	56	Male
**R. Maddison, 2019**	New Zealand	Intervention study	Effective	Single health condition	Cardiovascular disease not specified	Cardiovascular	>18	162	61	Male
**Y. Ko, 2019**	South Korea	Intervention study	Effective	Single health condition	Hip fracture or post hip arthroplasty	Musculoskeletal	> 65	34	76.7	Female
**W. Ji, 2019**	South Korea	Intervention study	Effective	Single health condition	Lung cancer	Cancer	< 80	64	59.2	Male
**J. Hou, 2019**	China	Intervention study	Effective	Single health condition	Low back pain	Musculoskeletal	Other	168	50.2	Balanced

**R. Hwang N. R. Morris A. , 2019**	Australia	Intervention study	Effective	Single health condition	Heart failure	Cardiovascular	_	53	67	Male
**W. Choi, 2019**	South Korea	Intervention study	Effective	Fragility or functional decline	_	_	> 65	60	76.25	Female
**S. C. Cramer, 2019**	United States of America	Intervention study	Effective	Single health condition	Stroke	Neurological	>18	124	61	Male
**D. K. Y. Chan, 2019**	Australia	Observational Study	Effective	Fragility or functional decline	_	_	_	369	82.9	Female
**T. Lindhardt, 2019**	Denmark	Intervention study	Effective	Fragility or functional decline	_	_	> 65	330	75	Balanced
**C. D. S. Langoni, 2019**	Brazil	Intervention study	Effective	Fragility or functional decline	_	_	> 60	52	72.25	Female
**P. Bernocchi, 2019**	Italy	Intervention study	Effective	Fragility or functional decline	_	_	> 65	283	78.6	Balanced
**H. R. Dial, 2019**	United States of America	Observational Study	Effective	Single health condition	Cognitive impairment including dementia or aphasia	Neurological	_	31	66.3	Balanced
**S. Isernia, 2019**	Italy	Intervention study	Effective	Single health condition	Neurological disease not specified	Neurological	< 80	107	77.2	Balanced
**T. H. Nøst, 2018**	Norway	Intervention study	Not effective	Single health condition	Chronic pain	Pain	>18	121	52.7	Female
**T. H. Nøst, 2018**	Norway	Intervention study	Not effective	Single health condition	Chronic pain	Pain	>18	121	53	Female
**S. Mohammadi-Kalaveh, 2018**	Iran	Intervention study	Effective	Multimorbidity	_	_	_	30	55.8	Balanced
**V. M. Mendoza-Núñez, 2018**	Mexico	Intervention study	No assessed	Fragility or functional decline	_	_	_	64	66	Female
**K. S. McGilton, 2018**	Canada	Intervention study	Effective	Single health condition	Stroke	Neurological	_	35	70	Male
**M. Markle-Reid, 2018**	Canada	Intervention study	Effective	Multimorbidity	_	_	> 65	159	_	Balanced
**E. Lycholip, 2018**	Netherlands	Intervention study	Not effective	Single health condition	Heart failure	Cardiovascular	>18	118	68	Male
**L. Lachance, 2018**	United States of America	Observational Study	Effective	Single health condition	Diabetes	Metabolic	>18	161	60.35	_
**J. T. Kamwesiga, 2018**	Uganda	Intervention study	Effective	Single health condition	Stroke	Neurological	>18	28	59.85	Female

**M. Inzitari, 2018**	Spain	Intervention study	No assessed	Fragility or functional decline	_	_	Other	185	81.6	Female
**S. A. Hicks, 2018**	United States of America	Observational Study	Effective	Fragility or functional decline	_	_	_	285	79.45	Balanced
**M. He, 2018**	China	Intervention study	Effective	Single health condition	Stroke	Neurological	_	2935	57.8	Balanced
**M. C. V. Gonzaga, 2018**	United States of America	Intervention study	Effective	Single health condition	Heart failure	Cardiovascular	_	20	_	Female
**N. Godtfredsen, 2018**	Denmark	Observational Study	Effective	Single health condition	COPD	Respiratory	_	581	68.1	Balanced
**C. B. Franse, 2018**	United Kingdom, Greece, Croatia, the Netherlands and Spain.	Intervention study	Effective	Fragility or functional decline	_	_	> 75	1844	79.5	Balanced
**I. H. J. Everink, 2018**	Netherlands	Observational Study	Effective	Fragility or functional decline	_	_	> 65	149	80.15	Female
**D. M. Ehde, 2018**	United States of America	Intervention study	Effective	Single health condition	Multiple sclerosis	Neurological	>18	163	52.2	_
**C. Dye, 2018**	United States of America	Intervention study	Effective	Multimorbidity	_	_	> 60	53	_	_
**P. W. Duncan, 2018**	United States of America	Descriptive study	No assessed	Single health condition	Stroke	Neurological	_	871	_	Balanced
**A. Döbler, 2018**	Germany	Intervention study	Effective	Single health condition	Diabetes	Metabolic	< 70	199	52	Male
**S. G. Dean, 2018**	United Kingdom	Intervention study	No assessed	Single health condition	Stroke	Neurological	>18	45	70.5	Male
**C. K. Clevenger, 2018**	United States of America	Descriptive study	Effective	Single health condition	Cognitive impairment including dementia or aphasia	Neurological	_	119	78.6	Female
**L. Chen, 2018**	China	Intervention study	Effective	Single health condition	Stroke	Neurological	>18	144	65.3	Male
**A. Avila, 2018**	Belgium	Intervention study	Not effective	Single health condition	Coronary heart disease	Cardiovascular	< 75	90	60.7	Male
**O. Adogwa, 2018**	United States of America	Descriptive study	Effective	Single health condition	Degenerative scoliosis	Musculoskeletal	> 65	125	73.3	Balanced
**Y. Zhang, 2018**	China	Intervention study	Effective	Single health condition	Coronary heart disease	Cardiovascular	_	130	70.1	Male

**P. Zhang, 2018**	China	Intervention study	Effective	Single health condition	Coronary heart disease	Cardiovascular	_	240	65.9	Balanced
**P. J. Whitehead, 2018**	United Kingdom	Intervention study	Effective	Fragility or functional decline	_	_	> 65	60	77	Balanced
**S. Ward, 2018**	United Kingdom	Observational Study	Effective	Single health condition	COPD	Respiratory	_	123	69.6	Balanced
**J. Wang, 2018**	China	Intervention study	Effective	Single health condition	Hip fracture or post hip arthroplasty	Musculoskeletal	_	389	55.6	Male
**M. R. J. van Lieshout, 2018**	Netherlands	Intervention study	Not effective	Fragility or functional decline	_	_	> 65	281	74	Balanced
**B. Valdivieso, 2018**	Spain	Intervention study	Effective	Multimorbidity	_	_	>18	472	73.08	Balanced
**M. Tistad, 2018**	Sweden	Intervention study	No assessed	Single health condition	Stroke	Neurological	_	68	72.6	Male
**E. Taube, 2018**	Sweden	Intervention study	Effective	Fragility or functional decline	_	_	> 65	153	81.5	Female
**J. L. Roberts, 2018**	United Kingdom	Intervention study	No assessed	Single health condition	Hip fracture or post hip arthroplasty	Musculoskeletal	> 65	61	_	_
**R. L. Reed, 2018**	Australia	Intervention study	Effective	Multimorbidity	_	_	> 60	254	_	Balanced
**D. Martel, 2018**	Canada	Intervention study	Effective	Fragility or functional decline	_	_	> 65	44	73.5	Female
**A. I. I. King, 2018**	New Zealand	Descriptive study	Effective	Fragility or functional decline	_	_	> 75	5	_	_
**K. Finlayson, 2018**	Australia	Intervention study	Effective	Fragility or functional decline	_	_	> 65	222	77.6	Female
**M. Melin, 2018**	Sweden	Intervention study	Effective	Single health condition	Heart failure	Cardiovascular	>18	82	75	Male
**R. L. Burton, 2018**	Canada	Intervention study	No assessed	Single health condition	Neurological disease not specified	Neurological	_	6	71.8	Female
**J. Hong, 2018**	Republic of Korea	Intervention study	Effective	Fragility or functional decline	_	_	> 65	23	79.8	Female
**M. Renda, 2018**	United States of America	Intervention study	Effective	Single health condition	Neurological disease not specified	Neurological	>18	4	59.7	Balanced
**K. D. Allen, 2018**	United States of America	Intervention study	Not effective	Single health condition	Osteoarthritis	Musculoskeletal	_	350	65.3	Female
**A. O'Moore K, 2018**	Australia	Intervention study	Effective	Single health condition	Osteoarthritis	Musculoskeletal	> 50	69	62	Balanced

**C. Anderson-Hanley, 2018**	United States of America	Intervention study	Effective	Fragility or functional decline	_	_	_	14	78.1	Female
**P. Bernocchi, 2018**	Italy	Intervention study	Effective	Single health condition	COPD, heart failure	Cardiovascular, respiratory	_	112	70	Male
**L. Zhang, 2017**	China	Intervention study	Effective	Single health condition	Coronary heart disease	Cardiovascular	< 75	126	63.1	Male
**F. A. Vega-Ramírez, 2017**	Spain	Observational Study	Effective	Fragility or functional decline	_	_	_	473	83	Balanced
**R. Uittenbroek, 2017**	Netherlands	Intervention study	Effective	Fragility or functional decline	_	_	> 75	1456	80.75	Balanced
**S. Tanaka, 2017**	Japan	Intervention study	Effective	Single health condition	Cognitive impairment including dementia or aphasia	Neurological	_	60	85.4	Female
**X. Ru, 2017**	China	Intervention study	Effective	Single health condition	Stroke	Neurological	_	964	68.95	Balanced
**S. Palmcrantz, 2017**	Sweden	Intervention study	No assessed	Single health condition	Stroke	Neurological	_	15	66	Balanced
**A.-F. Leclerc, 2017**	Belgium	Intervention study	Effective	Single health condition	Breast cancer	Cancer	_	209	53.35	Female
**P. Kitzman, 2017**	United States of America	Intervention study	Effective	Single health condition	Stroke	Neurological	_	30	65	Balanced
**I. H. J. Everink, 2017**	Netherlands	Intervention study	No assessed	Fragility or functional decline	_	_	> 65	189	81	Female
**M. S. Holstege, 2017**	Netherlands	Observational Study	Effective	Fragility or functional decline	_	_	_	743	80	Female
**K. M. Hjelle, 2017**	Norway	Descriptive study	No assessed	Fragility or functional decline	_	_	_	8	_	Balanced
**A. de Vos, 2017**	Netherlands	Descriptive study	No assessed	Fragility or functional decline	_	_	_	_	_	_
**N. Cecins, 2017**	Australia	Observational Study	Effective	Single health condition	COPD	Respiratory	_	251	72	Balanced
**N. Bleijenberg, 2017**	Netherlands Switzerland	Intervention study	Effective	Fragility or functional decline	_	_	Other	1791	85.05	Female
**R. N. Barker, 2017**	Australia	Observational Study	Effective	Fragility or functional decline	_	_	Other	206	61	Balanced
**J. J. Kraal, 2017**	Netherlands	Intervention study	Effective	Single health condition	Cardiovascular disease not specified	Cardiovascular	_	90	59.1	Male

**K. Brueggen, 2017**	Germany	Intervention study	Effective	Single health condition	Cognitive impairment including dementia or aphasia	Neurological	_	16	70	_
**J. Chen, 2017**	China	Intervention study	Effective	Single health condition	Stroke	Neurological	< 85	54	66.3	Male
**M. A. Mas, 2017**	Spain	Intervention study	Effective	Fragility or functional decline	_	_	_	849	83.4	Female
**S. Santana, 2017**	Portugal	Intervention study	Not effective	Single health condition	Stroke	Neurological	< 85	190	62	Balanced
**T. Ueda, 2017**	Japan	Intervention study	Effective	Fragility or functional decline	_	_	> 65	60	75.85	Female
**J. Hong, 2017**	Korea	Intervention study	Effective	Fragility or functional decline	_	_	> 65	23	81.85	Balanced
**R. Hwang J. Bruning N. R. Morris A. , 2017**	Australia	Intervention study	Effective	Single health condition	Heart failure	Cardiovascular	>18	53	67	Balanced
**A.-B. Zakrisson, 2016**	Sweden	Intervention study	Not effective	Single health condition	COPD	Respiratory	_	103	67.7	Balanced
**K. Young-Mee, 2016**	Korea	Intervention study	Effective	Fragility or functional decline	_	_	> 65	20	_	_
**A. Wolf, 2016**	Sweden	Intervention study	Effective	Single health condition	Cardiovascular disease not specified	Cardiovascular	Other	199	60	Male
**S. N. W. Vorrink, 2016**	Netherlands	Intervention study	Not effective	Single health condition	COPD	Respiratory	Other	157	62.5	Balanced
**M. van den Berg, 2016**	Australia	Intervention study	No assessed	Single health condition	Stroke	Neurological	_	63	67.3	Male
**M. Y. Tseng, 2016**	China	Intervention study	Effective	Single health condition	Hip fracture or post hip arthroplasty	Musculoskeletal	> 60	153	77.99	Female
**M.-Y. Tseng, 2016**	China	Intervention study	Effective	Single health condition	Hip fracture or post hip arthroplasty	Musculoskeletal	> 60	281	76.36	Female
**F. J. Tarazona-Santabalbina, 2016**	Spain	Intervention study	Effective	Fragility or functional decline	_	_	> 70	100	80	Balanced
**M. Smaerup, 2016**	Denmark	Intervention study	Not effective	Single health condition	Vestibular dysfunction	Sensory impairments	> 65	57	77.6	Balanced
**S. Shinkai, 2016**	Japan	Observational Study	Effective	Fragility or functional decline	_	_	> 65	_	_	_
**F. G. H. Ruikes, 2016**	Netherlands	Intervention study	Not effective	Fragility or functional decline	_	_	> 70	536	81.8	Female

**D. Rosen, 2016**	United States of America	Intervention study	Effective	Single health condition	Heart failure	Cardiovascular	Other	45	60	Female
**F. C. Pannill, 2016**	Netherlands	Intervention study	Not effective	Fragility or functional decline	_	_	> 65	674	80	Balanced
**M. J. Meunier, 2016**	United States of America	Descriptive study	Effective	Fragility or functional decline	_	_	Other	34	79	Balanced
**R. J. McNamara, 2016**	Australia	Intervention study	Effective	Single health condition	COPD, cardiovascular disease not specified	Cardiovascular, respiratory	_	32	72	Male
**A. McCluskey, 2016**	Australia	Intervention study	Not effective	Single health condition	Stroke	Neurological	>18	263	67	Balanced
**M. À. Mas, 2016**	Spain	Observational Study	Effective	Fragility or functional decline	_	_	> 65	270	83.5	Female
**D. L. Marsden, 2016**	Australia	Intervention study	Effective	Single health condition	Stroke	Neurological	>18	20	58.2	Female
**R. López-Liria, 2016**	Spain	Observational Study	Effective	Single health condition	Stroke	Neurological	_	145	69.25	Balanced
**W. M. Looman, 2016**	Netherlands	Intervention study	Not effective	Fragility or functional decline	_	_	_	184	82	Female
**W. M. Looman, 2016**	Netherlands	Intervention study	Effective	Fragility or functional decline	_	_	> 75	377	82.05	Female
**C. Littlewood, 2016**	United Kingdom	Intervention study	No assessed	Single health condition	Rotator cuff tendinopathy	Musculoskeletal	>18	86	54.7	Balanced
**Y.-Y. Leung, 2016**	China (Hong Kong)	Intervention study	Effective	Single health condition	Inflammatory arthritis	Musculoskeletal	>18	99	51.9	Female
**G. Leung, 2016**	Canada	Intervention study	Effective	Fragility or functional decline	_	_	> 60	104	81.6	Female
**D. S. Kushner, 2016**	United States of America	Intervention study	Effective	Single health condition	Stroke	Neurological	> 75	58	82.9	Balanced
**A. Kono, 2016**	Japan	Intervention study	Effective	Fragility or functional decline	_	_	> 65	360	79.2	Female
**E. Kjerstad, 2016**	Norway	Intervention study	Effective	Fragility or functional decline	_	_	>18	46	79	Female
**A. Karlsson, 2016**	Sweden	Intervention study	Effective	Single health condition	Hip fracture or post hip arthroplasty	Musculoskeletal	> 70	205	82.9	Female
**F. Jones, 2016**	United Kingdom	Intervention study	Effective	Single health condition	Stroke	Neurological	_	78	65.25	Balanced
**K. Huson, 2016**	Canada	Intervention study	Effective	Fragility or functional decline	_	_	> 70	100	82.7	Balanced

**C. Graven, 2016**	Australia	Intervention study	Effective	Single health condition	Stroke	Neurological	_	110	69.9	Balanced
**K.-J. Franke, 2016**	Germany	Intervention study	Effective	Single health condition	COPD	Respiratory	_	44	63.3	Balanced
**H. L. Cameron-Tucker, 2016**	Australia	Intervention study	Not effective	Single health condition	COPD	Respiratory	>18	65	69	Balanced
**S. Calugi, 2016**	Italy	Intervention study	Effective	Single health condition	Stroke	Respiratory	>18	229	70.95	Male
**B. M. Buurman, 2016**	Netherlands	Intervention study	Not effective	Fragility or functional decline	_	_	> 65	674	79.85	Balanced
**J. Blom, 2016**	Netherlands	Intervention study	Effective	Fragility or functional decline	_	_	> 75	358	80	Balanced
**N. Bleijenberg, 2016**	Netherlands	Intervention study	Effective	Fragility or functional decline	_	_	> 60	3092	74	Balanced
**S. Knecht, 2016**	Germany	Observational Study	Effective	Single health condition	Stroke	Neurological	_	2294	_	_
**P. De Vriendt, 2016**	Belgium	Intervention study	Effective	Fragility or functional decline	_	_	> 65	168	80.4	Female
**R. S. Rasmussen, 2016**	Denmark	Intervention study	Effective	Single health condition	Stroke	Neurological	>18	71	78.5	Balanced
**G. Lewin, 2016**	Australia	Descriptive study	Effective	Fragility or functional decline	_	_	_	58	76.8	Female
**A. M. Meyer, 2016**	United States of America	Intervention study	Effective	Single health condition	Cognitive impairment including dementia or aphasia	Neurological	Other	17	67.6	Balanced
**A. Y. Zhang, 2015**	United States of America	Intervention study	Effective	Single health condition	Prostate cancer	Cancer	_	244	65.3	Male
**F. K. Y. Wong, 2015**	China	Intervention study	Effective	Single health condition	Stroke	Neurological	_	108	69.5	Female
**A. van Dijk-de Vries, 2015**	Netherlands	Intervention study	Not effective	Single health condition	Diabetes	Metabolic	_	264	64.5	Balanced
**S. van der Weegen, 2015**	Netherlands	Intervention study	Effective	Single health condition	COPD	Respiratory	< 70	199	57.8	Balanced
**H. P. A. van der Aa, 2015**	Netherlands and Belgium	Intervention study	Effective	Single health condition	Vision impairment	Sensory impairments	> 50	265	73.6	Female
**H. Umapathy, 2015**	Australia	Intervention study	Not effective	Single health condition	Osteoarthritis	Musculoskeletal	_	195	60.7	Female
**A. E. Scharlach, 2015**	United States of America	Observational Study	Effective	Fragility or functional decline	_	_	> 60	96	76	Female

**A. C. Pighills, 2015**	Australia	Intervention study	Not effective	Fragility or functional decline	_	_	> 65	153	75.6	Balanced
**J. D. Piette, 2015**	United States of America	Intervention study	Effective	Single health condition	Heart failure	Cardiovascular	_	331	67.85	Male
**R. Pérez-Cuevas, 2015**	Mexico	Intervention study	Effective	Fragility or functional decline	_	_	> 65	239	77	Female
**S. M. Mosleh, 2015**	Scotland	Observational Study	Effective	Single health condition	Cardiovascular disease not specified	Cardiovascular	_	305	61.1	Male
**R. J. Mays, 2015**	United States of America	Intervention study	Not effective	Single health condition	Peripheral artery disease	Cardiovascular	Other	20	65.35	Female
**R. Maddison, 2015**	New Zealand	Intervention study	Not effective	Single health condition	Cardiovascular disease not specified	Cardiovascular	_	171	60.2	Male
**P. Lou, 2015**	China	Intervention study	Effective	Single health condition	COPD	Respiratory	_	8217	61.5	Balanced
**S. A. Lear, 2015**	Canada	Intervention study	Effective	Single health condition	Cardiovascular disease not specified	Cardiovascular	_	78	60.05	Female
**D. S. Kushner, 2015**	United States of America	Intervention study	Effective	Fragility or functional decline	_	_	> 75	524	83.15	Female
**L. Kidd, 2015**	United Kingdom	Intervention study	Effective	Single health condition	Stroke	Neurological	_	26	64	Balanced
**J. Garvey, 2015**	Ireland	Intervention study	Effective	Multimorbidity	_	_	>18	50	66.25	Female
**A. Forster, 2015**	United Kingdom	Intervention study	Not effective	Single health condition	Stroke	Neurological	_	800	71.7	Balanced
**M. P. Foley, 2015**	United States of America	Intervention study	Effective	Single health condition	Cancer not specified	Cancer	>18	59	59	Female
**C. Ekelund, 2015**	Sweden	Intervention study	Effective	Fragility or functional decline	_	_	> 65	158	_	Balanced
**J. Edgren, 2015**	Finland	Intervention study	Effective	Single health condition	Hip fracture or post hip arthroplasty	Musculoskeletal	> 60	81	80	Female
**N. Cuperus, 2015**	Netherlands	Intervention study	Not effective	Single health condition	Osteoarthritis	Musculoskeletal	_	147	58.5	Female
**P. Coventry, 2015**	United Kingdom	Intervention study	Effective	Multimorbidity	_	_	_	387	58.5	Male
**R. A. Clark, 2015**	Australia	Intervention study	Effective	Single health condition	Heart failure	Cardiovascular	>18	5	61.6	_
**A. K. Chang, 2015**	Korea	Intervention study	Effective	Single health condition	Stroke	Neurological	> 60	38	67.95	Balanced

**D. B. Bekelman, 2015**	United States of America	Intervention study	Not effective	Single health condition	Heart failure	Cardiovascular	_	384	67.6	Male
**I. . Frederix D. Hansen K. Coninx P. , 2015**	Belgium	Intervention study	Effective	Single health condition	Cardiovascular disease not specified	Cardiovascular	_	140	61	Balanced
**S. McKenna, 2015**	Australia	Intervention study	No assessed	Single health condition	Stroke	Neurological	_	24	64.7	Balanced
**J. D. , 2015**	India	Intervention study	Effective	Single health condition	Stroke	Neurological	>18	104	60	Balanced
**E. Piotrowicz, 2015**	Poland	Intervention study	Effective	Single health condition	Heart failure	Cardiovascular	_	131	56.4	Male
**H. Tunt, 2015**	Norway	Intervention study	Effective	Fragility or functional decline	_	_	>18	61	79	Female
**N. S. Tielemans, 2015**	Netherlands	Intervention study	Not effective	Single health condition	Stroke	Neurological	>18	113	57	Balanced
**A. Winkel, 2015**	Denmark	Intervention study	Effective	Fragility or functional decline	_	_	> 65	91	79.6	Female
**T. Taule L. I. St, 2015**	Norway	Intervention study	Not effective	Single health condition	Stroke	Neurological	_	154	73	Balanced
**D. N. Kiosses, 2015**	United States of America	Intervention study	Effective	Multimorbidity	_	_	_	74	67.6	Female
**S. F. Metzelthin, 2015**	Nethelerlands	Intervention study	Not effective	Fragility or functional decline	_	_	> 70	346	77.2	_

Supplementary Material 5

Supplementary Table 2. Rehabilitation programs' most important characteristics

First author, year	Level of care	Mode of service delivery	Interventions for rehabilitation	Providers	Health workers	Multidisciplinary rehabilitation
**Diaz Baquero, A. A., 2022**	Specialized health care	Inpatient, outpatient	Cognitive training	Health workers	Psychologist, therapist not specified	No
**Liang, C. C., 2022**	Primary health care	Community	Social care and support, education and advice on self-care, home visits, therapeutic exercises, therapeutic recreation	Peers and volunteers	_	No
**Porta, F. L., 2022**	Specialized health care	Outpatient, home	Therapeutic exercises, home visits, assessment of risk for falls, assessment of person-centred goals and priorities for health care, education and advice on self-care, multicomponent care and or rehabilitation programme, assessment of polypharmacy	Health workers	Physical therapist, geriatrician, PRM physicians, other physicians	Yes
**Yee, D. K. H., 2022**	Specialized health care	Inpatient	Discharge planning, rehabilitation coordination and management, multicomponent care and or rehabilitation programme	Health workers	Other physicians, geriatrician, PRM physicians	Yes
**Poulsen, L. K., 2022**	Specialized health care	Inpatient, community, home	Rehabilitation coordination and management, multicomponent care and or rehabilitation programme, home visits, therapeutic exercise, follow up visits, assessment of person-centred goals and priorities for health care, education and advice on self-care, discharge planning, occupational therapy	Health workers	Physical therapist, occupational therapist	No
**Stathi, A., 2022**	Primary health care	Community	Therapeutic exercise, therapeutic recreation, education and advice on self-care, education to influence lifestyle behaviours, behavioural interventions	Health workers	Exercise professionals	No
**Tekin, F., 2022**	Primary health care	Home	Therapeutic exercise, assessment of risk for falls, assessment of functioning	Health workers	Physical therapist	No
**Hoedemakers, M., 2022**	Multiple levels of care	Home, community, outpatient	Comprehensive geriatric assessment, case management, assessment of person-centred goals and priorities for health care, assessment of functioning, multicomponent care and or rehabilitation programme, therapeutic exercise, occupational therapy	Health workers, informal caregivers and family	Nurse, social workers, physical therapist, occupational therapist, geriatricians	Yes
**Ropke, A., 2022**	Multiple levels of care	Inpatient, home, community	Assessment of functioning, follow up visits, therapeutic exercise, assessment of person-centred goals and priorities for health care, occupational therapy	Health workers	Occupational therapist, physical therapist	No
**Bennell, K. L., 2022**	Specialized health care	Home	Counselling for weight management, assessment of person-centred goals and priorities for health care, therapeutic exercise, education about nutrition, behavioural interventions	Health workers	Physical therapist, dieticians	No
**Chong, E., 2022**	Specialized health care	Inpatient, home	Comprehensive geriatric assessment, discharge planning, education and advice on self-care, follow-up visits, caregiver's education and training	Health workers	General practitioner, physical therapist, pharmacist, nurse	Yes
**Kam Yuet Wong, F., 2022**	Multiple levels of care	Inpatient, home	Case management, discharge planning, follow up visits, home visits, health status monitoring, rehabilitation coordination and management, education and advice on self-care, caregiver's education and training, assessment of functioning, assessment of person-centred goals and priorities for health care, problem solving skills training, training for activities of daily living, therapeutic exercises	Health workers	Physical therapist, occupational therapist, speech and language therapists, neurologist, PRM physicians	Yes
**Love, 2022**	Specialized health care	Home	Follow up visits, home visits, training for activities of daily living, therapeutic exercises, assessment of person-centred goals and priorities for health care, occupational therapy, rehabilitation coordination and management, environmental	Health workers	Other physicians, physical therapist, occupational therapist, social workers,	Yes

			adaptations, provision and training in the use of assistive products, social care and support		dieticians, speech and language therapist	
**Kalav, S., 2022**	Specialized health care	Inpatient, home, outpatient	Discharge planning, education on self-care, follow up visits, health status monitoring,	Health workers, the patient	Nurse, multidisciplinary team not specified	Yes
**Ullrich, P., 2022**	Primary health care	Home	Education and advice on self-care, education about physical activity and exercise, motivational interventions, therapeutic exercise, training for activities of daily living, assessment of environment, assessment of functioning, home visits	Health workers	Exercise professionals	No
**Mora-Traverso, M., 2022**	Specialized health care	Telerehabilitation	Multicomponent care and or rehabilitation programme, education on self-care care, caregivers' education and training, occupational therapy, therapeutic exercise	Health workers	Physical therapist, occupational therapist	No
**Gustavson, A. M., 2022**	Multiple levels of care	Eldercare institution, home	Therapeutic exercise, education about physical activity and exercise, assessment of person-centred goals and priorities for health care, functioning monitoring	Health workers	Physical therapist, physical therapist assistants	No
**Tao, G., 2022**	Specialized health care	Outpatient, telerehabilitation	Therapeutic exercise, functioning assessment, peer support or peer support group, motivational interventions	Health workers, peers and volunteers	Trainers not specified	No
**Yang, L., 2022**	Specialized health care	Eldercare institution	Education and advice on self-care, cognitive training, caregivers' education and training, assessment of person-centred goals and priorities for health care, multicomponent care and or rehabilitation programme, coordination of care and management of care processes, training for activities of daily living, therapeutic exercise, therapeutic recreation	Health workers, informal caregivers and family	Neurologist, nurse, therapist not specified, social workers	Yes
**Furuta, K., 2022**	Primary health care	Community	Education and advice on self-care, assessment of person-centred goals and priorities for health care, therapeutic exercise, education about physical activity and exercise, education to influence lifestyle behaviours	Health workers	Occupational therapist, gerontologists	No
**Li, C. T., 2022**	Specialized health care	Home, telerehabilitation, outpatient	Training for activities of daily living, therapeutic exercise, occupational therapy, home based assistance for people with functional limitations in activities of daily living, follow up visits, functioning monitoring, health status monitoring, assessment of person-centred goals and priorities for health care	Health workers	Occupational therapist, physicians not specified	No
**Tan, J. Y., 2022**	Specialized health care	Outpatient	Health status monitoring, multicomponent care and or rehabilitation programme, therapeutic exercise, case management, training for activities of daily living	Health workers	Geriatrician, other physicians, pharmacist, social workers	Yes
**U. N. Yadav, 2021**	Multiple levels of care	Outpatient	Education and counselling in self-management, motivational activities, case management	The patient	_	_
**S. Tanaka, 2021**	Specialized health care	Eldercare institution	Cognitive rehabilitation, therapeutic exercise	Health workers	Physical therapist, occupational therapist	No
**A. Sondell, 2021**	Specialized health care	Outpatient, home	Comprehensive assessment of functioning, multicomponent rehabilitation programme, activities of daily living skills training, provision and training in the use of assistive products, social care and support plan, psychological interventions	Health workers	Occupational therapist, nurse, psychologist, social workers	Yes
**W. Rietkerk, 2021**	Specialized health care	Outpatient	Comprehensive geriatric assessment, assessment of person-centred goals and priorities for health care	Health workers	General practitioner, nurse, social workers, geriatrician	Yes
**M. E. Ribbink, 2021**	Specialized health care	Inpatient	Comprehensive geriatric assessment, assessment of person-centred goals and priorities for health care, multicomponent rehabilitation programme, environmental modifications, rehabilitation coordination and management, discharge planning	Health workers	Nurse, physical therapist, social workers, occupational therapist, other	Yes
**R. Quigley, 2021**	Multiple levels of care	Outpatient, inpatient	Comprehensive geriatric assessment, case management, rehabilitation coordination and management	Health workers	Geriatrician, nurse, general practitioner, nurse	Yes

**C. L. Peiris, 2021**	Specialized health care	Home, outpatient	Comprehensive assessment of functioning, therapeutic exercise, education and counselling on physical activity, education and counselling on weight loss, education and counselling on nutrition	Health workers	Physical therapist, geriatrician, nurse, dieticians	Yes
**M. Morri, 2021**	Multiple levels of care	Home, outpatient, inpatient	Comprehensive assessment of functioning, multicomponent rehabilitation programme	Health workers, informal caregivers and family	Physical therapist, nurse	No
**H. Kim, 2021**	Primary health care	Eldercare institution	Assessment of frailty, comprehensive assessment of functioning, case management, assessment of person-centred goals and priorities for health care, caregivers' education and training	Health workers	Nurse	No
**A. Flynn, 2021**	Multiple levels of care	Outpatient, telerehabilitation	Therapeutic exercise, education and counselling in self-management, follow-up	Health workers	Physical therapist	No
**R. De Luca, 2021**	Specialized health care	Telerehabilitation	Assessment of nutritional status, assessment of emotional functions, assessment of cognitive functions, case management, health status monitoring, social care and support plan, psychological interventions	Health workers	Nurse, social workers, psychologist, dieticians	Yes
**S. Coskun, 2021**	Multiple levels of care	Outpatient, inpatient	Assessment of health status, assessment of person-centred goals and priorities for health care, case management, multicomponent rehabilitation programme, discharge planning, follow-up	Health workers, informal caregivers and family	Nurse, other physicians, physical therapist, dieticians	Yes
**Y. Baba, 2021**	Primary health care	Eldercare institution	Therapeutic exercise, occupational therapy	Health workers	Other	No
**Coletta, A. M., 2021**	Specialized health care	Outpatient, telerehabilitation	Therapeutic exercise, assessment of person-centred goals and priorities for health care, assessment of exercise capacity	Health workers	PRM physicians, exercise professionals	No
**Lamberti, N., 2021**	Specialized health care	Outpatient, telerehabilitation	Assessment of exercise capacity, therapeutic exercise	_	_	_
**Maniscalco, M., 2021**	Specialized health care	Outpatient	Education about nutrition, therapeutic exercise, psychological interventions	Health workers	Multidisciplinary team not specified	Yes
**Otero, P., 2021**	Primary health care	Telerehabilitation	Cognitive training, psychological interventions, education to influence lifestyle behaviours, therapeutic recreation	The patient	_	_
**Tseng, M. Y., 2021**	Multiple levels of care	Inpatient, home	Comprehensive geriatric assessment, discharge planning, follow-up visits, caregivers' education and training, home visits, therapeutic exercise, assessment of person-centred goals and priorities for health care	Health workers	Nurse	No
**Kim, S., 2021**	Primary health care	Community	Therapeutic exercise	Health workers	Exercise professionals, community health worker	No
**Sun, F. C., 2021**	Primary health care	Community	Therapeutic exercise, music therapy	Health workers	Physical therapist, music therapist	No
**Farinha, C., 2021**	Primary health care	Community	Therapeutic exercise	Health workers	Exercise professionals	No
**Hayden, M. C., 2021**	Specialized health care	Inpatient	Therapeutic exercise, assessment of person-centred goals and priorities for health care, occupational therapy, health status monitoring, psychological interventions, education and advice on self-care	Health workers	Other physicians, social workers, psychologist, occupational therapist	Yes
**Barker, K. L., 2021**	Primary health care	Home	Assessment of functioning, assessment of person-centred goals and priorities for health care, therapeutic exercise, provision and training in the use of assistive products	Health workers	Physical therapist, physical therapist assistants	No

**Meisingset, I., 2021**	Primary health care	Home, eldercare institution	Therapeutic exercise, home visits, follow up visits, occupational therapy, assessment of functioning, assessment of person-centred goals and priorities for health care, social care and support	Health workers	Physical therapist, occupational therapist, home care personnel	Yes
**Al Chikhanie, Y., 2021**	Specialized health care	Outpatient	Therapeutic exercise, education and advice on self-care, psychological interventions, education about physical activity and exercise	_	_	_
**Suikkanen, S., 2021**	Primary health care	Home	Therapeutic exercise, education about nutrition, education about physical activity and exercise	Health workers	Physical therapist	No
**Brouwers, R. W. M., 2021**	Specialized health care	Outpatient, telerehabilitation	Functioning monitoring, follow up visits, therapeutic exercise	Health workers	Physical therapist	No
**Patel, N., 2021**	Specialized health care	Inpatient	Multicomponent care and or rehabilitation programme, speech therapy, therapeutic exercise, swallowing therapy, occupational therapy, training for activities of daily living, cognitive training, psychological interventions, rehabilitation coordination and management, provision and training in the use of assistive products, pharmacological therapy	Health workers	Occupational therapist, speech and language therapists, other physicians, psychologist	Yes
**Yi, D., 2021**	Primary health care	Home, telerehabilitation	Therapeutic exercise, assessment of risk for falls, environmental adaptations, home visits, follow up visits	Health workers	Physical therapist	No
**Chi, Y. C., 2021**	Specialized health care	Telerehabilitation	Education and advice on self-care, education about physical activity and exercise	Health workers	Physicians not specified, social workers, community workers, exercise professionals	Yes
**Gutierrez-Espinoza, H., 2021**	Specialized health care	Outpatient	Thermal modalities, therapeutic exercises	Health workers	Physical therapist	No
**Su, S. F., 2021**	Specialized health care	Inpatient, outpatient	Assessment of emotional functions, education and advice on self-care, motivational interventions, therapeutic exercise, discharge planning, follow up visits	Health workers, the patient	Nurse, other physicians	No
**Hosteng, K. R., 2021**	Primary health care	Eldercare institution	Education about physical activity and exercise, motivational interventions, functioning monitoring, education to influence lifestyle behaviours	The patient	_	_
**Chiu, E. C., 2021**	Specialized health care	Home	Assessment of person-centred goals and priorities for health care, occupational therapy, training in activities of daily living	Health workers	Occupational therapist	No
**Martinez-Velilla, N., 2021**	Specialized health care	Inpatient	Discharge planning, therapeutic exercise, education about physical activity and exercise, training on activities of daily living, assessment of person-centred goals and priorities for health care	Health workers, the patient	Physical therapist	No
**Gutierrez-Lopez, L., 2021**	Specialized health care	Eldercare institution	Assessment of health status, assessment of functioning, assessment of exercise capacity, therapeutic exercise	_	_	_
**Sen, E. I., 2021**	Primary health care	Home	Therapeutic exercise	_	_	_
**Sok, S., 2021**	Primary health care	Community	Motivational interventions, therapeutic exercise, cognitive training	Health workers	Nurse	No
**Jungreitmayr, S., 2021**	Primary health care	Telerehabilitation	Therapeutic exercise	The patient	_	_
**Liang, C. K., 2021**	Primary health care	Community	Therapeutic exercise, education about nutrition, cognitive training, education and advice on self-care	_	_	_

**Oh, S. L., 2021**	Primary health care	Home, outpatient	Therapeutic exercises, education and advice on self-care, education to influence lifestyle behaviours, education about nutrition, counselling for weight management	Health workers, the patient	Exercise professionals, physicians not specified, dieticians, nurse	Yes
**Pakrad, F., 2021**	Specialized health care	Telerehabilitation, outpatient	Therapeutic exercise, education and advice on self-care, education to influence lifestyle behaviours, follow up visits, health status monitoring, assessment of person-centred goals and priorities for health care	Health workers, the patient	_	_
**Siercke, M., 2021**	Specialized health care	Community	Therapeutic exercises, education and advice on self-care, education to influence lifestyle behaviours, motivational interventions, follow up visits, functioning monitoring, education about nutrition, caregivers' education and training, assessment of person-centred goals and priorities for health care	Health workers	Physical therapist, nurse, dieticians	Yes
**Tou, N. X., 2021**	Primary health care	Community	Therapeutic exercise	Health workers	Exercise professionals	No
**Monteblanco Cavalcante, M., 2021**	Specialized health care	Eldercare institution	Therapeutic exercise, cognitive training	Health workers	Physical therapist	No
**Borges-Machado, F., 2021**	Primary health care	Outpatient	Therapeutic exercise	Health workers	Exercise professionals	No
**Murphy, S. L., 2021**	Specialized health care	Outpatient, telerehabilitation	Thermal modalities, manual therapy, therapeutic exercises, occupational therapy	Health workers	Occupational therapist	No
**Bruce, J., 2021**	Specialized health care	Outpatient	Education about physical activity and exercise, therapeutic exercise, education and advice on self-care	Health workers	Physical therapist	No
**Rosado, H., 2021**	Specialized health care	Outpatient	Therapeutic exercise, cognitive training	Health workers	Rehabilitation scientist	No
**Tian, R., 2021**	Specialized health care	Outpatient	Social care and support, cognitive training, training for activities of daily living, emotional support, language therapy, education and advice on self-care	Health workers	Physicians not specified, nurses	No
**Noh, E. Y., 2021**	Multiple levels of care	Home, community, outpatient	Home based assistance for people with functional limitations in activities of daily living, case management, emotional support, cognitive training, education and advice on self-care, medication review dose adjustment cessation and or modification, environmental adaptations, therapeutic recreation, assessment of person-centred goals and priorities for health care	Health workers, peers and volunteers	Social workers, nurse, physicians not specified	Yes
**Aguilera-Godoy, A., 2021**	Specialized health care	Outpatient	Therapeutic exercise, thermal modalities, manual therapy, provision and training in the use of assistive products	Health workers	Physical therapist	No
**Mentz, R. J., 2021**	Specialized health care	Inpatient, outpatient, telerehabilitation	Assessment of person-centred goals and priorities for health care, discharge planning, therapeutic exercises, assessment of environment, follow up visits	Health workers	_	_
**Pepera, G., 2021**	Primary health care	Eldercare institution	Therapeutic exercise	Health workers	Physical therapist	No
**Rince, G., 2021**	Specialized health care	Outpatient	Therapeutic exercise, assessment of risk for falls, training for activities of daily living, assessment of person-centred goals and priorities for health care	Health workers	Physical therapist	No
**Arena, S. K., 2021**	Primary health care	Community, telerehabilitation	Therapeutic exercise, assessment of risk for falls, motivational interventions, education to influence lifestyle behaviours, environmental adaptations, assessment of environment, functioning monitoring	Health workers	Physical therapist	No
**Scherrenberg, M., 2021**	Specialized health care	Outpatient, telerehabilitation	Motivational interventions, therapeutic exercise, functioning monitoring, education about physical activity and exercise, follow up visits	The patient	_	_

**Mao, H. F., 2021**	Primary health care	Community	Therapeutic exercise, cognitive training, training in activities of daily living, motivational interventions, therapeutic recreation, assessment of person-centred goals and priorities for health care	Health workers	Occupational therapist	No
**Rafiq, M. T., 2021**	Specialized health care	Outpatient	Therapeutic exercise, motivational interventions, follow up visits	_	_	_
**Bagkur, M., 2021**	Primary health care	Telerehabilitation	Therapeutic exercise	Health workers	Physical therapist	No
**Backer, H. C., 2021**	Specialized health care	Outpatient, telerehabilitation	Therapeutic exercise, manual therapy, functioning monitoring	Health workers, the patient	Physical therapist	No
**Katri Maria, T., 2021**	Specialized health care	Telerehabilitation, home	Multicomponent care and or rehabilitation programme, therapeutic exercise, home visits, follow up visits, behavioural interventions, assessment of person-centred goals and priorities for health care	Health workers	Physical therapist	No
**Ozen, S., 2021**	Specialized health care	Outpatient	Therapeutic exercise, therapeutic recreation, training for activities of daily living, occupational therapy	Health workers	Physical therapist, occupational therapist	No
**Soukkio, P. K., 2021**	Specialized health care	Home	Therapeutic exercise, training for activities of daily living, assessment of person-centred goals and priorities for health care, education about physical activity and exercise, education about nutrition	Health workers	Physical therapist, occupational therapist	No
**Woo, J., 2021**	Primary health care	Outpatient, community	Therapeutic exercise, assessment of risk for falls, training for activities of daily living, assessment of person-centred goals and priorities for health care, cognitive training, social care and support plan	Health workers	Nurse, physical therapist, dieticians, optometrist	Yes
**Lim, J. H., 2021**	Specialized health care	Home	Therapeutic exercise, home visits	Health workers	Physical therapist	No
**Song, C. Y., 2021**	Specialized health care	Outpatient	Therapeutic exercise, cognitive training, education and advice on self-care, assessment of person-centred goals and priorities for health care	Health workers	Physical therapist, occupational therapist	No
**Z. Wu, 2020**	Specialized health care	Telerehabilitation	Multicomponent rehabilitation programme, assessment of person-centred goals and priorities for health care, caregivers' education and training	Health workers, informal caregivers and family	Other physicians, nurse, physical therapist	Yes
**J. Uddin, 2020**	Primary health care	Outpatient, home	Therapeutic exercise, education and counselling on self-management, health status monitoring	Health workers	Physical therapist	No
**K. A. Spencer, 2020**	Specialized health care	Outpatient	Assessment of person-centred goals and priorities for health care, assessment of cognitive functions, provision and training in the use of assistive products	_	_	_
**Y. Song, 2020**	Specialized health care	Telerehabilitation	Assessment of exercise capacity, therapeutic exercise	Health workers	Other physicians	No
**H. N. Smith, 2020**	Multiple levels of care	Eldercare institution, home	Discharge planning, case management, multicomponent rehabilitation programme, provision and training in the use of assistive products, environmental modifications, education and counselling on self-management	Health workers	Nurse, social workers, psychologist, speech and language therapist, dieticians	Yes
**J. Sedlock, 2020**	Specialized health care	Inpatient	Multicomponent rehabilitation programme, discharge planning, therapeutic exercise, occupational therapy	Health workers	Nurse, physical therapist, occupational therapist, dieticians	Yes
**J. Room, 2020**	Specialized health care	Home	Multicomponent rehabilitation programme, assessment of environment, provision and training in the use of assistive products	Health workers	Occupational therapist, physical therapist	No
**V. Provencher, 2020**	Multiple levels of care	Inpatient, home	Comprehensive assessment of functioning, assessment of person-centred goals and priorities for health care, discharge planning, assessment of environment, health status monitoring, provision of assistive products	Health workers	Occupational therapist	No

**M. Parsons, 2020**	Specialized health care	Inpatient	Comprehensive assessment of functioning, assessment of person-centred goals and priorities for health care, multicomponent rehabilitation programme, rehabilitation coordination and management, case management	Health workers	Nurse, physical therapist, occupational therapist, other	Yes
**A. Nakayama, 2020**	Specialized health care	Telerehabilitation	Education and counselling in self-management, therapeutic exercise, health status monitoring	Health workers	Other physicians, nurse	No
**I. Nabutovsky, 2020**	Specialized health care	Telerehabilitation	Assessment of health status, assessment of nutritional status, assessment of exercise capacity, assessment of emotional functions, multicomponent rehabilitation programme, therapeutic exercise, education and counselling on self-management, health status monitoring, psychological interventions	Health workers	Nurse, psychologist, dieticians	Yes
**J. S. Myers, 2020**	Specialized health care	Home, community	Assessment of cognitive functions, cognitive rehabilitation	Health workers	PRM physicians, psychologist	No
**J. Miller, 2020**	Primary health care	Community	Education and counselling in self-management, behavioural interventions	Health workers, the patient	Physical therapist	No
**M. Markle-Reid, 2020**	Multiple levels of care	Outpatient, home	Assessment of person-centred goals and priorities for health care, case management, multicomponent rehabilitation programme, follow-up, problem solving skills training, caregivers' education and training, education and counselling in self-management	Health workers	Occupational therapist, physical therapist, speech and language therapists, social workers	Yes
**J. Mann, 2020**	Multiple levels of care	Outpatient	Comprehensive geriatric assessment, case management	Health workers	Other physicians, nurse, occupational therapist, general practitioner	Yes
**W.-Y. Li, 2020**	Specialized health care	Outpatient	Health status monitoring, education and counselling on nutrition, education and counselling on physical activity	The patient	_	_
**M. Kraepelien, 2020**	Specialized health care	Telerehabilitation	Psychological interventions	Health workers	Psychologist	No
**E. H. Koolen, 2020**	Multiple levels of care	Outpatient, inpatient	Assessment of health status, comprehensive assessment of functioning, assessment of person-centred goals and priorities for health care, behavioural interventions, education and counselling in self-management, health status monitoring, case management, pharmacological therapy, optimization of pharmacological therapy, motivational activities	Health workers	Physical therapist, occupational therapist, psychologist, nurse, social workers	Yes
**M. V. Knudsen, 2020**	Specialized health care	Telerehabilitation	Therapeutic exercise, education and counselling on nutrition, education and counselling in self-management, social care and support plan	Health workers	Physical therapist, nurse, dieticians	Yes
**O. Kamoen, 2020**	Specialized health care	Telerehabilitation, outpatient	Education and counselling in self-management, health status monitoring, follow-up	Health workers	Nurse, other physicians	No
**H. Hansen, 2020**	Specialized health care	Telerehabilitation	Therapeutic exercise, education and counselling in self-management	_	_	_
**A. M. Gustavson, 2020**	Specialized health care	Inpatient	Therapeutic exercise, activities of daily living skills training, rehabilitation coordination and management	Health workers	Occupational therapist, physical therapist	No
**A. W. Deng, 2020**	Multiple levels of care	Inpatient, home	Pharmacological therapy, multicomponent rehabilitation programme, education and counselling in self-management, follow-up, case management, health status monitoring	Health workers	PRM physicians, nurse, general practitioner, other physicians	Yes
**J. de Batlle, 2020**	Multiple levels of care	Telerehabilitation	Assessment of health status, assessment of person-centred goals and priorities for health care, health status monitoring, case management education and counselling in self-management	Health workers, the patient	_	No

**S. S. Conroy, 2020**	Specialized health care	Telerehabilitation	Therapeutic exercise	Health workers	Occupational therapist	No
**D. Hevey, 2020**	Primary health care	Community	Peer support or peer support group, education and counselling in self-management, assessment of person-centred goals and priorities for health care, behavioural interventions	Peers and volunteers, the patient	_	No
**D. S. Lipardo, 2020**	Specialized health care	Community	Therapeutic exercise, cognitive training	Health workers	Physical therapist, occupational therapist	No
**K. Laver, 2020**	Specialized health care	Telerehabilitation	Comprehensive assessment of functioning, assessment of environment, caregivers' education and training, assessment of family and caregivers" needs, knowledge, and skills, activities of daily living skills training	Health workers	Occupational therapist, nurse	No
**A. K. C. Wong, 2019**	Primary health care	Community	Comprehensive assessment of functioning, assessment of person-centred goals and priorities for health care, education and counselling in self-management, social care and support plan, case management, assessment of environment, follow-up	Health workers, the patient	Nurse, community health worker, social workers	Yes
**K. von Storch, 2019**	Primary health care	Telerehabilitation	Assessment of person-centred goals and priorities for health care, health status monitoring, education and counselling in self-management, behavioural interventions, motivational activities	The patient	_	_
**M. Ranner, 2019**	Specialized health care	Outpatient	Comprehensive assessment of functioning, assessment of person-centred goals and priorities for health care, activities of daily living skills training, problem solving skills training	Health workers	Occupational therapist	No
**B. B. Nilsson, 2019**	Primary health care	Outpatient	Therapeutic exercise, education and counselling in self-management, motivational activities	Health workers	Physical therapist, other physicians, psychologist	Yes
**R. Maddison, 2019**	Specialized health care	Telerehabilitation	Behavioural interventions, education and counselling on physical activity or exercise, therapeutic exercise, motivational activities	Health workers	Community health worker	No
**Y. Ko, 2019**	Multiple levels of care	Outpatient, inpatient	Assessment of emotional functions, assessment of health status, assessment of fall risk, assessment of frailty, emotional support, activities of daily living skills training, therapeutic exercise, education and counselling in self-management, discharge planning, motivational activities	Health workers	Nurse	No
**W. Ji, 2019**	Specialized health care	Telerehabilitation	Assessment of person-centred goals and priorities for health care, therapeutic exercise, health status monitoring	Health workers	Other physicians, nurse	No
**J. Hou, 2019**	Specialized health care	Telerehabilitation	Pharmacological therapy, therapeutic exercise, education and counselling on physical activity or exercise, follow-up	Health workers	Other physicians	No
**R. Hwang N. R. Morris A. , 2019**	Specialized health care	Telerehabilitation	Education and counselling in self-management, therapeutic exercise, health status monitoring	Health workers	Physical therapist, nurse	No
**W. Choi, 2019**	Specialized health care	Telerehabilitation	Therapeutic exercise	Health workers	Physical therapist	No
**S. C. Cramer, 2019**	Specialized health care	Telerehabilitation	Education and counselling in self-management, activities of daily living skills training, assessment of person-centred goals and priorities for health care, therapeutic exercise, follow- up	Health workers	Physical therapist, occupational therapist	No
**D. K. Y. Chan, 2019**	Specialized health care	Eldercare institution	Occupational therapy, physical therapy, social care and support plan	Health workers	Physical therapist, occupational therapist, geriatrician	Yes
**T. Lindhardt, 2019**	Multiple levels of care	Inpatient, outpatient	Education and counselling in self-management, behavioural interventions, motivational activities, follow-up	Health workers	Nurse	No

**C. D. S. Langoni, 2019**	Primary health care	Community	Therapeutic exercise, motivational activities	_	_	_
**P. Bernocchi, 2019**	Specialized health care	Telerehabilitation	Comprehensive assessment of functioning, education and counselling in self-management, assessment of environment, assessment of fall risk, therapeutic exercise, follow-up, health status monitoring, functioning monitoring, caregivers' education and training	Health workers, informal caregivers and family	Physical therapist, nurse	No
**H. R. Dial, 2019**	Specialized health care	Telerehabilitation	Speech therapy and language therapy	The patient	_	_
**S. Isernia, 2019**	Multiple levels of care	Outpatient, telerehabilitation	Cognitive training, therapeutic exercise, assessment of person-centred goals and priorities for health care, follow-up, motivational activities	Health workers	Physical therapist, psychologist	No
**T. H. Nøst, 2018**	Primary health care	Community	Education and counselling in self-management, education and counselling on physical activity or exercise, psychological interventions, behavioural interventions	Health workers, the patient	Physical therapist	No
**T. H. Nøst, 2018**	Primary health care	Community	Education and counselling in self-management, psychological interventions, behavioural interventions, therapeutic exercise, peer support group	Health workers, the patient	Physical therapist	No
**S. Mohammadi-Kalaveh, 2018**	Specialized health care	Outpatient	Therapeutic exercise, psychological interventions, education and counselling on nutrition, education and counselling in self-management	Health workers	Nurse, physical therapist, psychologist, dieticians	Yes
**V. M. Mendoza-Núñez, 2018**	Primary health care	Community	Education and counselling in self-management	Health workers	Geriatrician	No
**K. S. McGilton, 2018**	Specialized health care	Inpatient	Assessment of person-centred goals and priorities for health care, provision and training in the use of assistive products, language therapy	Health workers	Nurse	No
**M. Markle-Reid, 2018**	Primary health care	Community	Assessment of person-centred goals and priorities for health care, education and counselling on nutrition, education and counselling in self-management, follow-up, peer support or peer support group, case management, motivational activities	Health workers, peers and volunteers, the patient	Nurse, dieticians, other	Yes
**E. Lycholip, 2018**	Primary health care	Telerehabilitation	Assessment of health status, health status monitoring, education and counselling in self-management	Health workers	Nurse	No
**L. Lachance, 2018**	Primary health care	Community	Peer support or peer support group, education and counselling in self-management, social care and support plan, case management, caregivers' education and training	Health workers	Community health worker	No
**J. T. Kamwesiga, 2018**	Primary health care	Telerehabilitation	Assessment of person-centred goals and priorities for health care, occupational therapy, activities of daily living skills training, caregivers' education and training	Health workers, informal caregivers and family	Occupational therapist	No
**M. Inzitari, 2018**	Primary health care	Community	Assessment of frailty, assessment of health status, comprehensive assessment of functioning, optimization of pharmacological therapy, assessment of nutritional status, therapeutic exercise, education and counselling on physical activity or exercise, education and counselling in self-management, education and counselling on nutrition	Health workers	Nurse, physical therapist, social workers, general practitioner	Yes
**S. A. Hicks, 2018**	Specialized health care	Telerehabilitation	Education and counselling in self-management, health status monitoring	Health workers	Nurse	No
**M. He, 2018**	Multiple levels of care	Inpatient, outpatient	Case management, discharge planning, assessment of health status, physical therapy	Health workers	General practitioner, nurse, physical therapist	Yes
**M. C. V. Gonzaga, 2018**	Specialized health care	Inpatient	Education and counselling in self-management	The patient	_	_

**N. Godtfredsen, 2018**	Primary health care	Community	Assessment of health status, therapeutic exercise, education and counselling in self-management, education and counselling on nutrition, social care and support plan, psychological interventions, education and counselling on physical activity or exercise	Health workers	Nurse, physical therapist, general practitioner, dieticians	Yes
**C. B. Franse, 2018**	Multiple levels of care	Community	Assessment of health status, comprehensive assessment of functioning, assessment of person-centred goals and priorities for health care, assessment of fall risk, assessment of emotional functions, assessment of frailty, case management, follow-up, optimization of pharmacological therapy, therapeutic exercise, social care and support plan	Health workers	Other physicians, nurse	No
**I. H. J. Everink, 2018**	Multiple levels of care	Inpatient	Case management, discharge planning, comprehensive assessment of functioning, multicomponent rehabilitation program, caregivers' education and training	Health workers, informal caregivers and family	Nurse, general practitioner, physical therapist, occupational therapist	Yes
**D. M. Ehde, 2018**	Primary health care	Telerehabilitation, community	Follow-up, education and counselling in self-management, behavioural interventions, education and counselling on nutrition	Health workers, the patient	Therapist not specified	No
**C. Dye, 2018**	Primary health care	Community	Education and counselling in self-management, health status monitoring, assessment of health status, comprehensive assessment of functioning, assessment of person-centred goals and priorities for health care, assessment of fall risk, follow-up, education and counselling on nutrition, education and counselling on physical activity or exercise, social care and support plan, case management, caregivers' education and training	Peers and volunteers, the patient	_	No
**P. W. Duncan, 2018**	Multiple levels of care	Inpatient, outpatient	Assessment of person-centred goals and priorities for health care	Health workers, informal caregivers and family	General practitioner, physical therapist, occupational therapist, speech and language therapist	Yes
**A. Döbler, 2018**	Specialized health care	Telerehabilitation	Assessment of person-centred goals and priorities for health care, behavioural interventions, education and counselling in self-management, education and counselling on physical activity or exercise, education and counselling on nutrition, social care and support plan, assessment of emotional functions, caregivers' education and training, motivational activities	Health workers	Other	No
**S. G. Dean, 2018**	Primary health care	Community, home	Assessment of person-centred goals and priorities for health care, therapeutic exercise, education and counselling in self-management, education and counselling on physical activity or exercise, follow-ups visits	Health workers	Community health worker	No
**C. K. Clevenger, 2018**	Primary health care	Eldercare institution	Assessment of person-centred goals and priorities for health care, social care and support plan, caregivers' education and training	Health workers, informal caregivers and family	Geriatrician, nurse, social workers	Yes
**L. Chen, 2018**	Specialized health care	Inpatient	Assessment of person-centred goals and priorities for health care, comprehensive assessment of functioning, follow-up, assessment of health status, education and counselling in self-management, problem solving skills training, peer support or peer support group, discharge planning	Health workers, informal caregivers and family, the patient	Nurse	No
**A. Avila, 2018**	Specialized health care	Outpatient, home	Health status monitoring, therapeutic exercise, education and counselling on physical activity or exercise, follow-up	Health workers	Physical therapist	No
**O. Adogwa, 2018**	Specialized health care	Inpatient	Assessment of health status, comprehensive assessment of functioning, multicomponent rehabilitation programme	Health workers	Geriatrician	No
**Y. Zhang, 2018**	Multiple levels of care	Community, home	Pharmacological therapy, therapeutic exercise, health status monitoring	Health workers, informal caregivers and family	General practitioner	No

**P. Zhang, 2018**	Multiple levels of care	Outpatient, inpatient	Discharge planning, education and counselling in self-management, education and counselling on healthy lifestyle behaviours, comprehensive assessment of functioning, follow-up, case management	Health workers	Nurse	No
**P. J. Whitehead, 2018**	Primary health care	Home	Assessment of environment, environmental modifications	Health workers	Occupational therapist	No
**S. Ward, 2018**	Specialized health care	Telerehabilitation	Education and counselling in self-management	Health workers	Occupational therapist, nurse, psychologist, other, dieticians	Yes
**J. Wang, 2018**	Specialized health care	Telerehabilitation	Therapeutic exercise, follow-up, functioning monitoring, health status monitoring, education and counselling in self-management	Health workers	Nurse	No
**M. R. J. van Lieshout, 2018**	Primary health care	Community	Social skills training, therapeutic exercise, education and counselling on self-management, problem-solving skills training, cognitive training, optimization of pharmacological therapy, education and counselling on nutrition	Health workers	Physical therapist, nurse, dieticians	Yes
**B. Valdivieso, 2018**	Primary health care	Telerehabilitation	Health status monitoring, follow-up, education and counselling in self-management, caregivers' education and training, social care and support plan	Health workers	Nurse	No
**M. Tistad, 2018**	Multiple levels of care	Inpatient, home	Assessment of person-centred goals and priorities for health care, multicomponent rehabilitation programme, activities of daily living skills training	Health workers	Occupational therapist	No
**E. Taube, 2018**	Primary health care	Home	Assessment of health status, comprehensive assessment of functioning, assessment of person-centred goals and priorities for health care, assessment of polypharmacy, assessment of emotional functions, assessment of fall risk, social care and support plan, emotional support, therapeutic exercise, social skills training, case management, follow-up, health status monitoring, functioning monitoring	Health workers	Physical therapist, nurse	No
**J. L. Roberts, 2018**	Multiple levels of care	Community	Assessment of person-centred goals and priorities for health care, occupational therapy, physical therapy, education and counselling in self-management, education and counselling on physical activity or exercise	Health workers	Physical therapist, occupational therapist, other	Yes
**R. L. Reed, 2018**	Specialized health care	Outpatient	Assessment of person-centred goals and priorities for health care, comprehensive assessment of functioning, psychological interventions, behavioural interventions	Health workers, the patient	_	No
**D. Martel, 2018**	Primary health care	Outpatient, home	Therapeutic exercise, follow-up	Health workers	Physical therapist	No
**A. I. I. King, 2018**	Primary health care	Community	Comprehensive assessment of functioning, comprehensive geriatric assessment, functioning monitoring, assessment of polypharmacy, case management, follow-up	Health workers, peers and volunteers	General practitioner, nurse	No
**K. Finlayson, 2018**	Multiple levels of care	Telerehabilitation, outpatient	Therapeutic exercise, follow-up	Health workers	Nurse	No
**M. Melin, 2018**	Specialized health care	Telerehabilitation	Education and counselling in self-management, education and counselling on, healthy lifestyle behaviours, health status monitoring	Health workers, the patient	Nurse	No
**R. L. Burton, 2018**	Specialized health care	Telerehabilitation	Assessment of person-centred goals and priorities for health care, cognitive rehabilitation, psychological interventions, assessment of cognitive functions, assessment of emotional functions	Health workers	Psychologist	No
**J. Hong, 2018**	Specialized health care	Telerehabilitation	Therapeutic exercise	Health workers	Psychologist	No
**M. Renda, 2018**	Specialized health care	Telerehabilitation	Comprehensive assessment of functioning, assessment of environment, environmental modifications, provision and training in the use of assistive products	Health workers	Occupational therapist	No

**K. D. Allen, 2018**	Specialized health care	Telerehabilitation	Comprehensive assessment of functioning, therapeutic exercise, motivational activities, functioning monitoring, health status monitoring, manual therapy, provision and training in the use of assistive products	Health workers	Physical therapist	No
**A. O'Moore K, 2018**	Specialized health care	Telerehabilitation	Education and counselling in self-management, psychological interventions	Health workers	Psychologist	No
**C. Anderson-Hanley, 2018**	Specialized health care	Telerehabilitation	Therapeutic exercise, cognitive training	_	_	_
**P. Bernocchi, 2018**	Specialized health care	Telerehabilitation	Therapeutic exercise, education and counselling in self-management	Health workers	Physical therapist, nurse	No
**L. Zhang, 2017**	Primary health care	Home, community	Comprehensive assessment of functioning, assessment of emotional functions, follow-up, therapeutic exercise, education and counselling on healthy lifestyle behaviours, peer support or peer support group, caregivers' education and training	Health workers	Nurse, dieticians, psychologist, other physicians	Yes
**F. A. Vega-Ramírez, 2017**	Primary health care	Home	Therapeutic exercise, activities of daily living skills training, assessment of person-centred goals and priorities for health care, assessment of fall risk, social care and support plan, caregivers' education and training	Health workers, informal caregivers and family	PRM physicians, physical therapist, occupational therapist	Yes
**R. Uittenbroek, 2017**	Primary health care	Community	Social care and support plan, assessment of person-centred goals and priorities for health care, follow-up, health status monitoring, functioning monitoring, education and counselling in self-management, rehabilitation coordination and management	Health workers	Geriatrician, nurse, social workers, general practitioner	Yes
**S. Tanaka, 2017**	Specialized health care	Eldercare institution	Cognitive rehabilitation, therapeutic exercise	Health workers	_	_
**X. Ru, 2017**	Primary health care	Community, home	Comprehensive assessment of functioning, multicomponent rehabilitation programme, education and counselling in self-management, caregivers' education and training	Health workers	Community health worker	No
**S. Palmcrantz, 2017**	Specialized health care	Telerehabilitation	Comprehensive assessment of functioning, therapeutic exercise	Health workers	Physical therapist	No
**A.-F. Leclerc, 2017**	Specialized health care	Outpatient	Therapeutic exercise, education and counselling in self-management, psychological interventions	Health workers	Physical therapist, psychologist	No
**P. Kitzman, 2017**	Primary health care	Community	Case management, discharge planning, follow-up, assessment of environment, environmental modifications, social care and support plan, education and counselling in self-management, caregivers' education and training	Health workers	Community health worker	No
**I. H. J. Everink, 2017**	Multiple levels of care	Inpatient	Rehabilitation coordination and management, case management, discharge planning, comprehensive assessment of functioning, assessment of person-centred goals and priorities for health care, multicomponent rehabilitation programme	Health workers, informal caregivers and family	Nurse, general practitioner, physical therapist, occupational therapist	Yes
**M. S. Holstege, 2017**	Specialized health care	Eldercare institution	Assessment of person-centred goals and priorities for health care, case management, multicomponent rehabilitation programme, caregivers' education and training	Health workers	Geriatrician, physical therapist, nurse	Yes
**K. M. Hjelle, 2017**	Multiple levels of care	Home	Rehabilitation coordination and management, follow-up, assessment of person-centred goals and priorities for health care, assessment of environment, environmental modifications, provision and training in the use of assistive products, activities of daily living skills training, therapeutic exercise, education and counselling in self-management	Health workers	Physical therapist, occupational therapist, other	Yes
**A. de Vos, 2017**	Specialized health care	Inpatient	Comprehensive assessment of functioning, caregivers' education and training, follow-up, case management, multidisciplinary geriatric care	Health workers	Other physicians, physical therapist, occupational therapist, social workers	Yes

**N. Cecins, 2017**	Primary health care	Community	Therapeutic exercise, pharmacological therapy, education and counselling in self-management, follow-up	Health workers	Physical therapist	No
**N. Bleijenberg, 2017**	Primary health care	Home	Case management, follow-up, functioning monitoring, health status monitoring, assessment of person-centred goals and priorities for health care, comprehensive geriatric assessment, assessment of family and caregivers'' needs, knowledge, and skills, multidisciplinary geriatric care, education and counselling in self-management, caregivers' education and training, management of urinary incontinence	Health workers	Nurse	No
**R. N. Barker, 2017**	Primary health care	Outpatient	Assessment of person-centred goals and priorities for health care, rehabilitation coordination and management, multicomponent rehabilitation programme, education and counselling in self-management, follow-up	Health workers	Speech and language therapists, occupational therapist, nurse, social workers, dieticians	Yes
**J. J. Kraal, 2017**	Multiple levels of care	Home	Therapeutic exercise, assessment of person-centred goals and priorities for health care, health status monitoring, follow-up, education and counselling on physical activity or exercise	Health workers	Physical therapist	No
**K. Brueggen, 2017**	Specialized health care	Outpatient	Cognitive rehabilitation, activities of daily living skills training, psychological interventions, assessment of person-centred goals and priorities for health care, comprehensive assessment of functioning, provision and training in the use of assistive products, caregivers' education and training	Health workers	Occupational therapist, psychologist	No
**J. Chen, 2017**	Specialized health care	Telerehabilitation	Therapeutic exercise, activities of daily living skills training, electromyography-triggered neuromuscular stimulation, assessment of person-centred goals and priorities for health care, caregivers' education and training	Health workers, informal caregivers and family	Occupational therapist, physical therapist	No
**M. A. Mas, 2017**	Multiple levels of care	Inpatient, home	Multicomponent rehabilitation programme	Health workers	Physical therapist, occupational therapist, PRM physicians, geriatrician	Yes
**S. Santana, 2017**	Multiple levels of care	Home	Discharge planning, provision and training in the use of assistive products, environmental modifications, assessment of environment, education and counselling in self-management, caregivers' education and training, activities of daily living skills training, case management, comprehensive functioning assessment, rehabilitation coordination and management	Health workers	Physical therapist, occupational therapist, psychologist	Yes
**T. Ueda, 2017**	Specialized health care	Home	Education and counselling in self-management, environmental modifications, assessment of environment, provision and training in the use of assistive products, assessment of fall risk	Health workers	Physical therapist	No
**J. Hong, 2017**	Specialized health care	Telerehabilitation	Therapeutic exercise	The patient	_	_
**R. Hwang J. Bruning N. R. Morris A. , 2017**	Specialized health care	Telerehabilitation	Therapeutic exercise, education and counselling in self-management	Health workers	Occupational therapist, nurse, social workers, dieticians	Yes
**A.-B. Zakrisson, 2016**	Primary health care	Outpatient	Education and counselling in self-management, therapeutic exercise, education and counselling on healthy lifestyle behaviours, caregivers' education and training	Health workers	Physical therapist, occupational therapist, nurse, social workers	Yes
**K. Young-Mee, 2016**	Primary health care	Community	Therapeutic exercise, psychological interventions, emotional support, therapeutic recreation, motivational activities	Health workers	Other, social workers	No
**A. Wolf, 2016**	Multiple levels of care	Telerehabilitation	Person centred care, education and counselling in self-management, health status monitoring, functioning monitoring	Health workers, the patient	Other physicians, nurse	No

**S. N. W. Vorrink, 2016**	Primary health care	Telerehabilitation	Assessment of person-centred goals and priorities for health care, therapeutic exercise, health status monitoring, functioning monitoring	Health workers	Physical therapist	No
**M. van den Berg, 2016**	Multiple levels of care	Inpatient, home	Multicomponent rehabilitation programme, rehabilitation coordination and management, discharge planning, follow-up, health status monitoring, functioning monitoring, caregivers' education and training, therapeutic exercise, motivational activities	Health workers, informal caregivers and family	Physical therapist	No
**M. Y. Tseng, 2016**	Multiple levels of care	Inpatient, home	Multicomponent rehabilitation programme, rehabilitation coordination and management, therapeutic exercise, comprehensive assessment of functioning, comprehensive geriatric assessment, assessment of family and caregivers'' needs, knowledge, and skills, assessment of person-centred goals and priorities for health care, discharge planning, follow-up, education and counselling in self-management, caregivers' education and training, social care and support plan	Health workers	Geriatrician, nurse, physical therapist, PRM physicians	Yes
**M.-Y. Tseng, 2016**	Multiple levels of care	Inpatient, home	Assessment of fall risk, assessment of polypharmacy, comprehensive assessment of functioning, comprehensive geriatric assessment, assessment of emotional functions, discharge planning, follow-up, multicomponent rehabilitation programme, therapeutic exercise, pharmacological therapy, psychological interventions, education and counselling on nutrition, social care and support plan, environmental modifications, assessment of environment	Health workers	Nurse, geriatrician	No
**F. J. Tarazona-Santabalbina, 2016**	Primary health care	Outpatient	Therapeutic exercise, health status monitoring	Health workers	Physical therapist, nurse	No
**M. Smaerup, 2016**	Specialized health care	Telerehabilitation	Functioning monitoring, therapeutic exercise	Health workers	Physical therapist	No
**S. Shinkai, 2016**	Primary health care	Community	Comprehensive geriatric assessment, functioning monitoring, education and counselling on healthy lifestyle behaviours, education and counselling on physical activity or exercise, therapeutic exercise, social care and support plan, education and counselling on nutrition	Health workers	General practitioner, nurse	No
**F. G. H. Ruikes, 2016**	Primary health care	Outpatient	Assessment of polypharmacy, rehabilitation coordination and management, case management, functioning monitoring, follow-up, health status monitoring, assessment of person-centred goals and priorities for health care, social care and support plan	Health workers	Geriatrician, nurse, general practitioner	Yes
**D. Rosen, 2016**	Primary health care	Telerehabilitation	Case management, education and counselling on nutrition, functioning monitoring, follow-up, health status monitoring, assessment of person-centred goals and priorities for health care, education and counselling in self-management, behavioural interventions, education and counselling on healthy lifestyle behaviours	Health workers, the patient	Social workers	No
**F. C. Pannill, 2016**	Multiple levels of care	Inpatient, eldercare, home	Comprehensive geriatric assessment, follow-up, discharge planning	Health workers	Nurse, general practitioner	No
**M. J. Meunier, 2016**	Multiple levels of care	Inpatient, outpatient	Assessment of person-centred goals and priorities for health care, multicomponent rehabilitation programme, pharmacological therapy, rehabilitation coordination and management, social care and support plan	Health workers	Occupational therapist, general practitioner, nurse, social workers, dieticians	Yes
**R. J. McNamara, 2016**	Primary health care	Community	Therapeutic exercise	Health workers	Physical therapist	No
**A. McCluskey, 2016**	Specialized health care	Inpatient	Therapeutic exercise, therapeutic recreation	Health workers	Physical therapist, occupational therapist, other	Yes

**M. À. Mas, 2016**	Primary health care	Home	Comprehensive geriatric assessment, assessment of person-centred goals and priorities for health care, rehabilitation coordination and management, activities of daily living skills training, therapeutic exercise, environmental modifications, assessment of fall risk	Health workers	Physical therapist, occupational therapist, other physicians, nurse	Yes
**D. L. Marsden, 2016**	Primary health care	Community	Comprehensive assessment of functioning, therapeutic exercise, assessment of person-centred goals and priorities for health care, follow-up, education and counselling on physical activity or exercise	Health workers	Physical therapist	No
**R. López-Liria, 2016**	Specialized health care	Home	Multicomponent rehabilitation programme, rehabilitation coordination and management, assessment of person-centred goals and priorities for health care	Health workers	Physical therapist, occupational therapist, PRM physicians	Yes
**W. M. Looman, 2016**	Multiple levels of care	Outpatient	Comprehensive assessment of functioning, functioning monitoring, rehabilitation coordination and management, multicomponent care, assessment of person-centred goals and priorities for health care	Health workers	Other physicians, nurse	No
**W. M. Looman, 2016**	Primary health care	Outpatient	Multicomponent rehabilitation programme, comprehensive assessment of functioning, assessment of cognitive functions, assessment of emotional functions, assessment of person-centred goals and priorities for health care, rehabilitation coordination and management, case management, assessment of fall risk, assessment of polypharmacy, management of urinary and bowel incontinence	Health workers	Physical therapist, general practitioner, nurse, other	Yes
**C. Littlewood, 2016**	Primary health care	Outpatient	Physical therapy, follow-up, education and counselling on physical activity or exercise	Health workers, the patient	Physical therapist	No
**Y.-Y. Leung, 2016**	Primary health care	Community	Education and counselling in self-management, peer support or peer support group, assessment of person-centred goals and priorities for health care, therapeutic exercise	Health workers, the patient	Nurse, community health worker	No
**G. Leung, 2016**	Specialized health care	Inpatient	Rehabilitation coordination and management, discharge management, physical therapy, occupational therapy, language and speech therapy, social care and support plan, therapeutic recreation	Health workers	Occupational therapist, nurse, speech and language therapists, general practitioner, dieticians	Yes
**D. S. Kushner, 2016**	Specialized health care	Inpatient	Discharge planning, comprehensive assessment of functioning, assessment of health status, assessment of environment, case management, rehabilitation coordination and management, multicomponent rehabilitation programme	Health workers	PRM physicians, other physicians, physical therapist	Yes
**A. Kono, 2016**	Primary health care	Home	Comprehensive assessment of functioning, functioning monitoring, assessment of cognitive functions, assessment of person-centred goals and priorities for health care, rehabilitation coordination and management	Health workers	Nurse, social workers, other	Yes
**E. Kjerstad, 2016**	Primary health care	Home	Comprehensive assessment of functioning, assessment of person-centred goals and priorities for health care, caregivers' education and training, education and counselling in self-management, activities of daily living skills training, provision and training in the use of assistive products, environmental modifications, social care and support plan, occupational therapy, physical therapy	Health workers	Physical therapist, occupational therapist	No
**A. Karlsson, 2016**	Specialized health care	Home	Assessment of health status, nutritional assessment, rehabilitation coordination and management, case management, comprehensive assessment of functioning, assessment of person-centred goals and priorities for health care, activities of daily living skills training, therapeutic exercise, assessment of fall risk, assessment of polypharmacy, wound care, environmental modifications, assessment of environment, provision and training in the use of assistive products, education and counselling in self-management	Health workers	Occupational therapist, nurse, social workers, dieticians	Yes

**F. Jones, 2016**	Primary health care	Community	Multicomponent rehabilitation programme, assessment of person-centred goals and priorities for health care, education and counselling in self-management	Health workers, the patient	Occupational therapist, physical therapist, speech and language therapists	Yes
**K. Huson, 2016**	Specialized health care	Inpatient	Cognitive rehabilitation, provision and training in the use of assistive products, therapeutic exercise, assessment of health status, assessment of nutritional status	Health workers, peers and volunteers	_	No
**C. Graven, 2016**	Multiple levels of care	Outpatient, home	Multicomponent rehabilitation programme, assessment of person-centred goals and priorities for health care, case management, functioning monitoring, assessment of fall risk, education and counselling in self-management	Health workers	Physical therapist	No
**K.-J. Franke, 2016**	Specialized health care	Telerehabilitation	Therapeutic exercise, functioning monitoring, motivational activities, follow-up	_	_	_
**H. L. Cameron-Tucker, 2016**	Primary health care	Telerehabilitation	Assessment of person-centred goals and priorities for health care, therapeutic exercise, education and counselling on healthy lifestyle behaviours, education and counselling in self-management, follow-up	Health workers	Nurse	No
**S. Calugi, 2016**	Primary health care	Community	Caregivers' education and training, education and counselling in self-management, education and counselling on healthy lifestyle behaviours, therapeutic exercise	Health workers	Other physicians, physical therapist	No
**B. M. Buurman, 2016**	Multiple levels of care	Inpatient, outpatient	Comprehensive geriatric assessment, assessment of environment, discharge planning, assessment of person-centred goals and priorities for health care	Health workers	Nurse	No
**J. Blom, 2016**	Multiple levels of care	Outpatient	Assessment of person-centred goals and priorities for health care, case management, assessment of cognitive functions, comprehensive assessment of functioning, follow-up, multicomponent rehabilitation programme	Health workers	General practitioner, nurse	No
**N. Bleijenberg, 2016**	Primary health care	Outpatient	Assessment of frailty, assessment of polypharmacy, comprehensive geriatric assessment, functioning monitoring, case management, follow-up, multicomponent rehabilitation programme, management of urinary incontinence	Health workers	General practitioner, nurse	No
**S. Knecht, 2016**	Specialized health care	Inpatient	Speech and language therapy, physical therapy, occupational therapy, sports-therapy, cognitive training, assessment of person-centred goals and priorities for health care, comprehensive assessment of functioning	Health workers	Occupational therapist, nurse, speech and language therapists, psychologist	Yes
**P. De Vriendt, 2016**	Primary health care	Community	Assessment of person-centred goals and priorities for health care, activities of daily living skills training, provision and training in the use of assistive products, caregivers' education and training	Health workers	Occupational therapist	No
**R. S. Rasmussen, 2016**	Multiple levels of care	Inpatient, home	Assessment of person-centred goals and priorities for health care, comprehensive assessment of functioning, activities of daily living skills training, multicomponent rehabilitation programme, assessment of cognitive functions, environmental modifications, assessment of environment, provision and training in the use of assistive products, social care and support plan, caregivers' education and training, discharge planning, rehabilitation coordination and management	Health workers	Physical therapist, occupational therapist, other physicians, nurse	Yes
**G. Lewin, 2016**	Primary health care	Home	Comprehensive assessment of functioning, therapeutic exercise, education and counselling in self-management, assessment of person-centred goals and priorities for health care, activities of daily living skills training, provision and training in the use of assistive products, multicomponent rehabilitation programme, rehabilitation coordination and management, pharmacological therapy, education and counselling on nutrition, management of urinary and bowel incontinence	Informal caregivers and family	_	No
**A. M. Meyer, 2016**	Specialized health care	Telerehabilitation, outpatient	Assessment of cognitive functions, language therapy	The patient	_	_

**A. Y. Zhang, 2015**	Specialized health care	Telerehabilitation, outpatient	Peer support or peer support group, therapeutic exercise, follow-up, education and counselling in self-management	Health workers, peers and volunteers	Nurse, psychologist	No
**F. K. Y. Wong, 2015**	Multiple levels of care	Inpatient, outpatient	Therapeutic exercise, discharge planning, follow-up, case management, comprehensive assessment of functioning, multicomponent rehabilitation programme, assessment of environment, caregivers' education and training, behavioural interventions, education and counselling on healthy lifestyle behaviours, education and counselling in self-management	Health workers	Nurse	No
**A. van Dijk-de Vries, 2015**	Primary health care	Outpatient	Assessment of person-centred goals and priorities for health care, comprehensive assessment of functioning, assessment of emotional functions, emotional support, education and counselling in self-management, problem solving skills training	Health workers, the patient	Nurse	No
**S. van der Weegen, 2015**	Primary health care	Telerehabilitation	Education and counselling in self-management, motivational activities, education and counselling on physical activity or exercise, functioning monitoring, assessment of person-centred goals and priorities for health care	Health workers	Nurse	No
**H. P. A. van der Aa, 2015**	Specialized health care	Outpatient	Low vision rehabilitation, assessment of emotional functions, problem solving skills training, psychological interventions	Health workers	Occupational therapist, social workers, psychologist	Yes
**H. Umapathy, 2015**	Specialized health care	Telerehabilitation	Assessment of emotional functions, assessment of health status, comprehensive assessment of functioning, education and counselling in self-management, education and counselling on healthy lifestyle behaviours, health status monitoring, functioning monitoring	The patient	_	_
**A. E. Scharlach, 2015**	Primary health care	Home	Comprehensive assessment of functioning, case management, assessment of environment, assistance in activities of daily living	Peers and volunteers	_	No
**A. C. Pighills, 2015**	Primary health care	Community	Comprehensive assessment of functioning, rehabilitation coordination and management, multicomponent rehabilitation programme	Health workers	Physical therapist, occupational therapist, other physicians	Yes
**J. D. Piette, 2015**	Multiple levels of care	Telerehabilitation	Education and counselling in self-management, caregivers' education and training, functioning monitoring	Health workers, informal caregivers and family	Other physicians, nurse	No
**R. Pérez-Cuevas, 2015**	Specialized health care	Outpatient	Rehabilitation coordination and management, comprehensive geriatric assessment, physical therapy, occupational therapy, psychological interventions, social skills training, education and counselling in self-management	Health workers	Geriatrician, psychologist, social workers, occupational therapist, physical therapist	Yes
**S. M. Mosleh, 2015**	Primary health care	Outpatient	Therapeutic exercise, education and counselling in self-management	Health workers	Physical therapist, nurse, psychologist, dieticians	Yes
**R. J. Mays, 2015**	Primary health care	Community	Therapeutic exercise, education and counselling in self-management, assessment of environment, functioning monitoring	_	_	_
**R. Maddison, 2015**	Specialized health care	Telerehabilitation	Therapeutic exercise, education and counselling in self-management, functioning monitoring, behavioural interventions	The patient	_	_
**P. Lou, 2015**	Primary health care	Community	Rehabilitation coordination and management, comprehensive assessment of functioning, follow-up, education and counselling in self-management, education and counselling on healthy lifestyle behaviours	Health workers	General practitioner, nurse, PRM physicians, dieticians	Yes
**S. A. Lear, 2015**	Specialized health care	Telerehabilitation	Therapeutic exercise, health status monitoring, education and counselling in self-management, education and counselling on nutrition	Health workers	Nurse, dieticians	Yes
**D. S. Kushner, 2015**	Specialized health care	Inpatient	Rehabilitation coordination and management, case management, discharge planning, assessment of person-centred goals and priorities for health care,	Health workers	Other physicians, physical therapist, speech and	Yes

			comprehensive assessment of functioning, multicomponent rehabilitation programme		language therapists, occupational therapist, nurse	
**L. Kidd, 2015**	Primary health care	Outpatient	Motivational activities, assessment of person-centred goals and priorities for health care, education and counselling in self-management	Health workers, the patient	Nurse	No
**J. Garvey, 2015**	Primary health care	Community	Education and counselling in self-management, assessment of person-centred goals and priorities for health care, education and counselling on healthy lifestyle behaviours, peer support or peer support group	Health workers, the patient	Occupational therapist	No
**A. Forster, 2015**	Primary health care	Community	Comprehensive assessment of functioning, multicomponent rehabilitation programme	Health workers	_	_
**M. P. Foley, 2015**	Primary health care	Community	Therapeutic exercise, comprehensive assessment of functioning, assessment of person-centred goals and priorities for health care	Health workers	Physical therapist	No
**C. Ekelund, 2015**	Multiple levels of care	Inpatient, outpatient	Assessment of person-centred goals and priorities for health care, multicomponent rehabilitation programme, rehabilitation coordination and management, assessment of frailty, comprehensive geriatric assessment, follow-up, discharge planning	Health workers	Occupational therapist, PRM physicians, nurse, social workers	Yes
**J. Edgren, 2015**	Specialized health care	Home	Assessment of person-centred goals and priorities for health care, therapeutic exercise, education and counselling on physical activity or exercise, provision and training in the use of assistive products, education and counselling in self-management, environmental modifications, assessment of environment, activities of daily living skills training, functioning monitoring, follow-up, assessment of fall risk, assessment of polypharmacy	Health workers	Physical therapist	No
**N. Cuperus, 2015**	Specialized health care	Telerehabilitation, outpatient	Comprehensive assessment of functioning, assessment of person-centred goals and priorities for health care, motivational activities, therapeutic exercise, activities of daily living skills training, multicomponent rehabilitation programme, education and counselling in self-management, education and counselling on physical activity or exercise, education and counselling on nutrition, functioning monitoring, follow-up	Health workers	Physical therapist, occupational therapist, nurse, dieticians	Yes
**P. Coventry, 2015**	Multiple levels of care	Outpatient	Behavioural interventions, optimization of pharmacological therapy, psychological interventions, assessment of emotional functions, education and counselling in self-management, education and counselling on healthy lifestyle behaviours, rehabilitation coordination and management	Health workers	Other physicians, nurse, psychologist	Yes
**R. A. Clark, 2015**	Primary health care	Community	Education and counselling in self-management	Health workers, the patient	_	_
**A. K. Chang, 2015**	Specialized health care	Eldercare institution	Social care and support plan, therapeutic recreation, caregivers' education and training, assistance in activities of daily living, assessment of person-centred goals and priorities for health care, therapeutic exercise, activities of daily living skills training	Health workers	Physical therapist, nurse	No
**D. B. Bekelman, 2015**	Multiple levels of care	Telerehabilitation	Health status monitoring, functioning monitoring, assessment of emotional functions, rehabilitation coordination and management, education and counselling in self-management, psychological interventions	Health workers	General practitioner, nurse	No
**I. . Frederix D. Hansen K. Coninx P. , 2015**	Specialized health care	Telerehabilitation	Education and counselling on healthy lifestyle behavioural interventions, psychological interventions, assessment of emotional functions, assessment of person-centred goals and priorities for health care, education and counselling on nutrition, follow-up, health status monitoring, assessment of health status	Health workers	Psychologist, dieticians	No
**S. McKenna, 2015**	Specialized health care	Community	Assessment of person-centred goals and priorities for health care, discharge planning, multicomponent rehabilitation programme, social care and support plan, behavioural interventions, education and counselling in self-management	The patient	_	No

**J. D. , 2015**	Multiple levels of care	Inpatient	Caregivers' education and training	Health workers	Physical therapist	No
**E. Piotrowicz, 2015**	Specialized health care	Telerehabilitation	Psychological interventions, assessment of person-centred goals and priorities for health care, therapeutic exercise, health status monitoring	Health workers	Physical therapist, nurse, psychologist	Yes
**H. Tunt, 2015**	Specialized health care	Outpatient	Comprehensive assessment of functioning, assessment of person-centred goals and priorities for health care, activities of daily living skills training, environmental modifications, assessment of environment, therapeutic exercise, rehabilitation coordination and management, education and counselling in self-management	Health workers	Speech and language therapists, physical therapist, occupational therapist, nurse	Yes
**N. S. Tielemans, 2015**	Specialized health care	Outpatient	Psychological interventions, education and counselling in self-management	Health workers, the patient	Psychologist, occupational therapist	No
**A. Winkel, 2015**	Primary health care	Home	Case management, follow-up, assessment of person-centred goals and priorities for health care, activities of daily living skills training, assessment of environment, environmental modifications, caregivers' education and training	Health workers	Occupational therapist	No
**T. Taule L. I. St, 2015**	Multiple levels of care	Inpatient, outpatient, home	Case management, discharge planning, physical therapy, occupational therapy, multicomponent rehabilitation programme, assessment of person-centred goals and priorities for health care, therapeutic exercise, activities of daily living skills training, cognitive training	Health workers	Physical therapist, occupational therapist	No
**D. N. Kiosses, 2015**	Specialized health care	Home	Psychological interventions, assessment of emotional functions, assessment of person-centred goals and priorities for health care, problem solving skills training, environmental modifications	Health workers	Social workers, psychologist, other	Yes
**S. F. Metzelthin, 2015**	Primary health care	Home	Assessment of frailty, multicomponent rehabilitation programme, assessment of person-centred goals and priorities for health care, rehabilitation coordination and management	Health workers	Physical therapist, occupational therapist, general practitioner, nurse	Yes
